# Algorithmic Identification of Murzymes and Murburn Mechanisms Based on Structural, Theoretical, Experimental, and Generic Features

**DOI:** 10.1155/bmri/2577941

**Published:** 2026-03-10

**Authors:** Kelath Murali Manoj, Vinith Rejathalal, Amrit Subramanian, Pathange Omkareshwara Rao, P. Unnikrishnan, K. P. Soman

**Affiliations:** ^1^ Amrita School of Artificial Intelligence, Coimbatore, Amrita Vishwa Vidyapeetham, Ettimadai, Tamil Nadu, India, amrita.edu; ^2^ Satyamjayatu: The Science & Ethics Foundation, Palakkad, Kerala, India

**Keywords:** algorithmic parsing of enzymes and mechanisms, heme proteins, murburn concept, murzymes

## Abstract

Murburn concept is a decade‐old theorization for explaining several cellular redox‐metabolic and electro/mechanophysiological outcomes. It stems from “mured burning”, connoting delocalized stochastic electron‐transfer processes, involving diffusible reactive species (DRS). The thermodynamic–kinetic–mechanistic (TKM) aspects of several cellular activities are seamlessly woven with the idea of murburn, also recently consolidated with quantitative models. Therefore, it is now opportune to algorithmically delineate the various features of protein/metabolic systems that generate, modulate/stabilize, or utilize DRS. Herein, we meta‐analyzed three simple/single heme‐protein systems: (1) extracellular heme‐haloperoxidase, (2) membrane‐bound cyclooxygenase, and (3) soluble hemoglobin; and also two complex/multiprotein systems (incorporating heme‐enzymes): (4) the hepatocyte xenobiotic metabolism proteins on endoplasmic reticulum, and (5) the mitochondrial oxidative phosphorylation machinery. We first tabulated an eight‐point comparison of the classical mechanistic models in literature with the murburn model for these five systems and also performed an internal consistency check for the classical models. Thereafter, we compared eight distinct postulates of both models for these systems and employed an Ockham′s razor‐based algorithm for the preferred mechanistic model. Further, based on 20 structural, theoretical, and experimental parameters, we employed decision‐tree/random‐forest (AI‐ML) methods to delineate murzymes (murburn systems) with total accuracy. Furthermore, we developed a simple web‐based portal for mechanistic parsing of murzymes from classical enzymes. Using the classification thus derived, we developed an LLM‐SVM–based model to demarcate murzymes (with ~84% accuracy), parameterizing only text‐descriptors in PDB (RCSB) files. Herein, we also provide a brief projection of how these novel mechanistic and algorithmic analyses impact research in redox/TKM‐enzymology.

## 1. Introduction

Derived from “mured burning”, murburn concept encompasses a set of factual correlations and deductions that establishes the necessity of diffusible reactive species (DRS: a collective term for radicals and other reactive intermediates) in physiology. Although molecules and unbound ions are the constitutive components of life, their thermodynamically spontaneous but kinetically controlled interactions with energizing radiations and in situ generated DRS are inevitable. We labeled this overlooked stochastic aspect of cellular functioning as “murburn” [1–3]. It can be contrasted with the rather elaborately orchestrated and deterministic molecular schemes of life, such as those governed by the long‐term acting, affinity‐binding based, sequential/serial logic of central dogma (DNA‐replication, RNA‐transcription, and protein‐translation) and the affinity‐binding–based interactomes of proteins thereof. That is: the classically perceived biological processes are contingent upon a high‐affinity “transition state” intermediate, as found in the established pair paradigms ensuring biological selectivity/specificity, like: catalytic enzyme‐substrate (ES), electron donor‐acceptor, signal receptor‐ligand, and immunological antibody‐antigen. Murburn reactions and their controls (and physical outcomes thereafter) are exercised at shorter timescales, in a rather unrestricted “stochastic realm” wherein freely diffusing/permeating agencies such as reactive radicals and energizing radiations drive the process. In such schemes, the outcomes result from favorable collisions and electron‐relays and are not obligatorily dependent on “deterministic topological affinity‐fits” among the interacting components [4]. Whereas the classical redox enzyme mechanisms involving radicals (e.g., ribonucleotide reductase or cyclooxygenase) employ a protein‐confined moiety (cobalamin‐derived or tyrosyl radical, respectively), murburn concept invokes DRS.

### 1.1. Update on Murburn Concept

Within the contexts of cellular function, metabolism can be defined as one (or more) reaction(s) catalyzed by (an array of) enzyme(s) to give specific product(s) of interest. Physiology can be understood as an aggregated outcome of such chemical/metabolic reactions, in conjunction with several other accompanying physical (electrical, mechanical, etc.) phenomena that might include radiating or falling‐dominos type phase changes and cascades, thereby altering macroscopic properties of the locus/cell. For an efficient governance of cellular function, particularly the instantaneous agenda of PCHEMS (powering, coherence, homeostasis, electro‐mechanical activities, and sensing‐response, etc.; the acute physicochemical features that qualify life), it is imperative that a murburn‐like viable/tangible and seamless logic must exist for connecting and coordinating the molecular to macroscopic transition (metabolic and physiological) of outcomes [1–3, 5, 6]. In this regard, we theorized the concept of “chemico‐electromagnetic matrix” (CEM), a maze of interacting molecules, ions, radicals, radiations within the cell, under the context of murburn concept [6]. In our recent communications, we had demonstrated murburn concept′s ECS (effective charge separation)‐based (a process that generates DRS) working of cells as simple chemical engines (or SCEs) to be the primordial “unintelligent evolutionary” logic [1, 6], which went on to lay down the foundations of biological intelligence (BI) seen today. We had also focused on the structure‐distribution‐architecture of proteins and organelles in several key systems [7] and thermodynamic–kinetic–mechanistic [2, 3, 8] aspects in related publications elsewhere. As per murburn concept, the ECS‐based generation of diffusible reactive (oxygen) species [DR(O)S] becomes obligatory for enabling most fundamental physiology, explaining the acute need for oxygen in sustaining life. The “ECS‐DRS‐SCE‐PCHEMS‐CEM” postulates of murburn explained the relevance of hitherto unrecognized and stochastic molecule‐unbound ion‐radical/radiation interactive equilibriums in routine cellular physiology. Some publications by researchers in recent times also deem DR(O)S in a more balanced or positive perspective, which could be seen as an indirect support of murburn concept [9, 10]. Several publications of late have also extensively discussed the murburn models of cellular metabolism and physiology, considering it in favorable light [11–14]. In fact, in line with our advocacy, a China‐centric international group′s publication [15] cited one and a half dozen publications on murburn concept from our group, exalting murburn concept as a pivotal founding principle of life (also listing ”murburn concept” as a keyword for their review article)!

### 1.2. Conflicts With Classical Perceptions

The classical ideas of enzyme function are advocated in prevailing textbooks [16–19]. In the classical perspective, Fischer′s “lock and key” (proposed at the end of the 19th century) mechanism accounts for selectivity/specificity and Koshland′s “induced fit” (proposed in the mid‐20th century) supposedly explained substrate diversity seen in some enzymes. Contemporary mechanisms have simulated protein dynamics and ensemble models to reason enzyme promiscuity (for reasoning how the same active site could bind and catalyze diverse substrate conversions) without requiring a separate, overarching theory. It is quite difficult owing to experimentally observed contraindications and also counter‐intuitive to accept the interpretations advocated by contemporary researchers that: (i) a small molecule like ubiquinone or small proteins like cytochrome *c*/*b* ^5^ could move deterministically as elements of “electron transport chains” [20–24], or that (ii) even hundreds of larger (and topologically diverse!) proteins like cytochrome P450 (CYP) could receive (or donate) electrons (from a unique reductase, CPR) purely based on topological features/facets that identify and specifically bind to their various known redox partners, to deterministically traffic electrons through an intra/interprotein long‐range tunneling mechanism [25–27]. However, the longstanding classical purview of cellular physiology deems the mandate prescribed by the central dogma (a gene produces a protein with a definite active site, which binds to a definite molecule/ion to regulate its turnover!) to be adequate for carrying out all cellular functions harmoniously. Although DRS like NO were well‐documented in literature and some such species were considered to have signaling roles in physiology [28–30], the vast amount of literature and the overwhelming perception deemed DRS as waste/toxic products that led to oxidative stress, disease states, and aging [31–34]. The classical perceptions also deem cellular membranes (with embedded proteins) to be “intelligent and proactive borders” that pump out ions, enabling trans‐membrane chemo‐electrical gradients that support diverse functions [16–19].

### 1.3. Delineating the Agenda of This Work

Since the last 2 decades, our group′s works pointed out anomalous observations, which suggested that some enzyme‐catalyzed reactions occur outside the active site (countering the “induced fit” interpretations) and the classical view also appeared superfluous in many cases, particularly substrate‐inhibition [35–37]. We have also grounded the ECS‐DRS murburn electron transfers (ETs) and murzyme catalysis in mathematical foundations, to demarcate it with respect to the Michaelis–Menten theorization for hyperbolic rate exhibited with respect to substrate concentration [38]. Yet, the majority of the research and teaching community have not noted the developments and continue to stick to an “authority‐enforced” consensus. Therefore, to address the apparent dichotomy in the classical and murburn perspectives, in our current interdisciplinary meta‐analysis, we consider protein structure and reaction mechanism with an algorithmic angle. Of the multiple redox heme‐protein functional scenarios (selected from diverse realms/contexts), we focus primarily on the probabilistic considerations in explaining reaction mechanisms. That is: how do we make an objective and unbiased decision for the determination/selection of a/the more viable/tangible explanation among the alternative options (classical versus murburn) available? OR, how can we algorithmically employ the yardsticks driving Ockham’s razor (principle of parsimony)? We also allude briefly to accompanying thermodynamic, kinetic, structural and other mechanistic considerations. With the current undertaking, we also provide for the first commoner‐accessible user‐interface for parsing proteins into the classical (working only via binding at the active site) versus murzyme (a protein that recruits DRS) classes.

### 1.4. Explicit Disclaimers

At the outset, we do not dispute the evidence or arguments in literature supporting the “Lock Key or Induced Fit” classical active‐site mechanisms. In fact, the controls employed in this study (e.g., replication–transcription–translation enzyme machinery, digestive enzymes like lipase‐protease‐carbohydrases, etc.) are classical examples of such mechanisms. Also, we do not claim any exclusivity of DROS over BROS (Bound Reactive Oxygen Species, like Compound I in Peroxidases or P450s). We merely consider the DROS‐based murburn theorization to be a larger set of events that is probabilistically preferred and which could also afford scopes for the operational relevance of BROS. That is: we state that there are overwhelming cases of DROS relevance in heme‐enzyme catalysis and the few cases of BROS relevance can be considered as a scenario where DROS exists in the bound state (thus giving BROS)! Herein, using the data available in literature and simple analytical measures, we peruse an algorithmic logic to support the murburn mechanistic thesis that DROS can also give physiologically relevant catalytic outcomes (many of which were erstwhile attributed to BROS!), and not all cases of DROS‐based outcomes are “undesired” or wasteful/toxic. The thermodynamic and kinetic necessity/argument and mathematical justifications for this premise have already been established via recent communications [6, 38].

## 2. Methodology

This work is a meta‐analysis and algorithm‐centered research piece, built extensively on earlier published data and information available from latest databases/reviews/textbooks.

### 2.1. System

**Figure 1:**
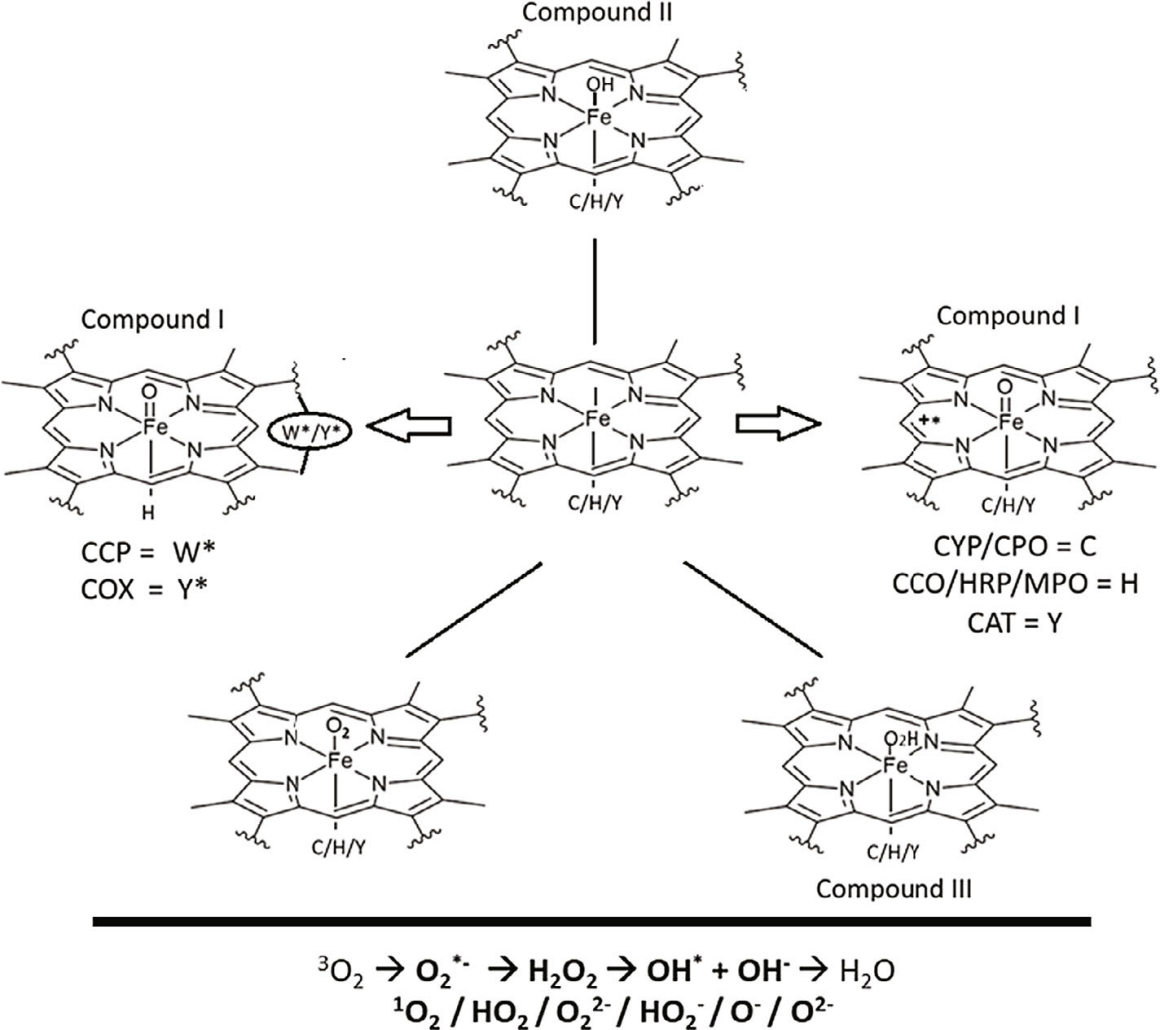
BROS (top panel) versus DROS (bottom panel). Please refer to the text for the details of acronyms of enzymes. The proximal ligand to the heme (C, cysteine; H. histidine; and Y, tyrosine), the distal pocket ambiance (e.g., hydrophilic in peroxidase and hydrophobic in P450s), the surface topographies/residues and the modalities of stabilization of reactive species vary in these heme‐enzyme systems.

Some heme‐containing proteins/enzymes are quintessential and ubiquitous features of living cells, right from early archaea to later mammals. We choose some of the salient and versatile redox‐active protein representatives like: cytochrome *c* peroxidase (CCP), cyclooxygenase (COX), cytochrome P450 (CYP), chloroperoxidase (CPO), cytochrome *c* oxidase (CCP), horseradish peroxidase (HRP), myeloperoxidase (MPO), catalase (CAT), etc. as the model systems for our study. Some known classical and selective/specific non‐redox enzyme systems, particularly the ones belonging to the core of central dogma, “the replication‐transcription‐translation machinery” are taken as controls. The hemoproteins contain a redox‐sensitive cofactor, a central Fe atom (*d*‐electrons) coordinated to a planar organic porphyrin possessing concatenated single‐double bonds (a *pi*‐electron cloud), making it colored (owing to high Soret band absorption). It is interesting to note that some such heme‐enzymes have been known to work at practically diffusion‐limited kinetics, approaching the limits of catalytic efficiency (e.g., catalase). The top panel of Figure 1 shows the schematic structures of various heme‐bound reactive oxygen species (BROS), like Compounds I, II, III, and so on of well‐known heme‐enzymes, as determined by sophisticated spectroscopic methods. The central Fe atom could be penta‐(with the distal site free) or hexa‐ligated (with cysteine, histidine, or tyrosine as the proximal ligand) and the Fe could be in formal oxidation states of *I* *I* ^+^ (as in hemoglobin Hb, and myoglobin, Mb) or even as high as *V* ^+^ (as in Compound I, wherein the fifth valency is accounted with a radical stabilized on the porphyrin or amino acid residue of the holoenzyme). The bottom panel of Figure 1 shows the representations of various DROS that could be formed/present in physiological realms (with or without polyvalent Fe species) during the cumulative one‐electron reductive steps of oxygen to water (within the window of approximately −400 to +800 mV). We use the DROS term to collectively represent superoxide, peroxide, hydroxyl radical, singlet oxygen, and all other such species that are hitherto documented or potentially generated (protonated or not, contingent upon milieu conditions) within the physiological milieu. As the 2e‐deficient electrophilic Compound I species is perceived to be the primary catalytic species in a wide range of heme‐proteins (with thiolate or histidylate or tyrosylate proximal amino‐acyl ligation) like peroxidases, P450s, oxygenases, and oxidases, we compare the mechanistic schemes involving this protagonist with the DROS in simple and complex metabolic systems.

### 2.2. Mechanistic Parsing Logic

The work is divided into three parts, with the latter ones being progressively developed and logically contingent upon the prior ones.

In the first part, we undertook two modular agendas: (i) an “Ockham′s razor” logic‐based algorithmic preference/selection of the favorable mechanistic postulates (i.e., of the two models, BROS vs. DROS mechanisms), and (ii) determining internal consistency within the BROS proposals (as the DROS proposal was “nonlimited” and with broad spectrum relevance, it was not subjected to this measure).

#### 2.2.1. Ockham′s Razor‐Based Preference Between Two Mechanistic Models for a Given Enzyme/System

Ockham′s razor implies that of all the competing models explaining a physical phenomenon, the simplest one is more likely to be closer to the truth than the others. Simplicity is therefore a talismanic benchmark that inspires confidence in the veracity of a scientific theory. In the context of cellular reaction logistics, we propose the following as the postulates‐based criteria against which a proposal can be evaluated for its simplicity. We considered six aspects of the two competing proposals:

Conformational changes: It is quite clearly the case that a model that uses a number of complex conformation adjustments is more complex than the one which does not.

Serial/parallel course of reactions: Since the electrochemical events take place in a medium populated by diverse substrates, a serial mechanism entails an assumption of unlikely sophistry at the molecular level for the preferential selection of a specific pathway. Parallel mechanism, on the other hand, views the processes as outcomes spontaneously determined by local environment. It is thus a more natural explanation. This view considers the system as an ensemble of concurrently occurring reaction paths.

Number of mechanistic steps: The number of intermediates and reaction steps involved in each pathway is a simple and obvious indicator of complexity.

Irreducible complexity: A process that (a priori) solicits/requires multiple components pre‐arranged in specific modalities for a rather simple outcome, and one which is rather unlikely to evolve but can only be created or fashioned by an intelligent operator is labeled “irreducibly complex”. This is a term originally introduced by Michael Behe.

Molecularity of key intermediates: Although multi‐molecular complexations are common in biological systems, there are ascertainable rationales for such complexations, as the affinities between the complexing agents are well established. When diverse molecules with very little biological connectivity are supposed to come together to form multimolecular complexes (which are deemed obligatory for the mechanistic pathways), such proposal can be deemed lesser favorable. Also, from a statistical perspective, a bimolecular intermediate is more likely to form than, for example, a tetra‐ or penta‐molecular transition state.

Index of probability: In the overall schemes, if there are many associative and dissociative processes, each one of these associations and dissociations can be given a probability score (e.g., the associated state has 1/2 and the dissociated state as 1/2; discounting the affinities of the interacting species). Now, when the schema is taken as a whole, it can give us a fair quantitative picture of the probabilistic window of a given proposal, knowing that serial processes would be a multiplied outcome of the individual probability score (with lower scores being unlikely routes).

Long‐range ET: When interprotein ETs are solicited in a mechanistic scheme, the Marcus outer sphere mechanism becomes unviable over distances of ~1 nm.

ES complex: The requirement for diverse molecules to form high affinity ES‐complex at a given deep‐seated active site is rather unlikely.

We coded a simple Python program that takes two models as inputs and systematically compares them on the above aspects. Each model is assigned a simplicity score based on its values for each of the parametric inputs. The model features are taken as binary for simplicity. This screening is for one enzyme or enzyme‐system at a time. Each model is designed as a class with the following attributes discussed above, and as logically summarized in Table 1.

**Table 1:** Postulates/features of a mechanistic model.

***No.***	***Attribute***	***Value range***	***Remarks***
1	Shape change	Binary {0,1}	Conformational changes needed
2	Serial	Binary {0,1}	Serial/sequential or parallel/unordered steps
3	Number of steps	1 to as many	Greater the number, lesser it is favored
4	Complexity	Binary {0,1}	Irreducibly complexity is disfavored
5	Molecularity	1 to as many	Molecules forming intermediates‐high/low
6	Probability	Event/outcomes	Association/dissociation events; 1/2 each
7	Long‐range ET	Binary {0,1}	Long‐distance outer sphere ET is unlikely
8	E‐S complex	Binary {0,1}	Diverse substrates unlikely to bind at active site

Simplicity score associated with a model is incremented based on the values of each of these features. The final score reflects the overall nature of the model. The models are finally compared on the basis of their scores, where higher score indicates greater admissibility.

#### 2.2.2. Internal Consistency Within the Classical Proposal and Features for a Given Enzyme/System

A theory is scientifically inadmissible in spite of clearing the simplicity test, if it fails to form a logically cogent/coherent whole. A fundamental requirement in this regard is that the theory should not have internally conflicting proposals. For example, in cytochrome P450 mediated outcomes, the BROS proposal invokes a Type I bound xenobiotic at the heme distal active site pocket to explain for the CYPs′ change in redox potential. However, it also seeks a freely rotating and unbound substrate at other instances to account for the high values obtained in internal kinetic isotope effects (KIEs) experiments [39]. Such options are mutually exclusive and cannot be admitted, disregarding the prevailing subjective consensus among recalcitrant experts and their followers! We have developed a simple algorithmic test for internal consistency of some such aspects for the classical models. (As murburn model accommodates all possible interactions, it is a larger set of events and therefore, it is not tested for such violations!) Table 2 lists a set of causes of inconsistency within the classical Michaelis–Menten paradigm. In the first four instances of Table 2, the second assertion (#) is incompatible with the first (^a^). In the last four cases, the first assertion itself is incompatible with the active‐site theorization for enzyme function.

**Table 2:** Internal consistency statements/premises for active‐site TS models.

***No.***	***Assertion 1*** ^***a***^	***Assertion 2#***
1	Active site access limited	No selectivity of substrates
2	Active site access limited	No specificity of products
3	Selectivity of substrates	Non‐integral stoichiometry
4	Substrate bound at active site	High int. KIE for substrate rxn.
5	Catalytic rate (*k*_cat_) > 109 M^−1^ s^−1^	
6	Substrate larger than active site volume	
7	Zeroth order kinetics at micromolar S	
8	K_M_ < K_d_	

*Note:* For explanation of the contents (^a^ and #), please refer to the citing text inline.

The above set of cross‐checks was coded into a simple program that takes a list of assertions and flags any possible mutual logical incompatibility in its constituent assertions and premises. This screening is for one enzyme or enzyme‐system at a time.

The positives and negatives for murzymes algorithmically classified with the logic above (the classical enzymes must not show any internal inconsistency and the murzymes must win over the classical postulates in the comparative screening) were taken as controls in Part 2. For training, we used 20 different structural, theoretical and experimental features of 10 enzymes or multiprotein systems of murzymes and classical enzymes. The “decision tree or random forest” logic derived from the same was used to sort a set of unknown/unclassified proteins/systems into murburn or classical systems. Based on this part′s results, the overall trends of each of the 20 features (and eight postulates) were tabulated.

Since the training data were of a very limited size and insufficient to cover the full scope of the biological interconnections, the identified consensus of parameters′ trends with discriminative potential (from decision tree and its ensemble version, random forest) was taken thereafter, each with its saliency weight proportional to that obtained by the classifiers. This list of features was then used to develop a rule‐based classification logic, to be used as the demarcating logic at the user‐friendly web portal. The resulting procedure can be summarized as follows: Each of the classes is assigned a count initialized to 0. The affinity of a test molecule to the classes is reflected in the magnitude of the respective count at the end of the procedure. The magnitude of the counts is incremented proportional to the degree of saliency derived from the two classifiers. For instance, the presence of the cofactor heme is identified as a salient indicator of murzyme (and its absence, therefore, is deemed classical). Hence, the count of the respective class is incremented by 1 depending on its presence or absence. The same logic was repeated across all the features with magnitude‐increments proportional to their assigned saliency weight. Classification is ultimately based on the relative magnitude of the count values. All algorithms are presented in GitHub and a simple web‐based portal for parsing enzymes is made available.

At a more practical level, the lack of a comprehensive and searchable database of murzymes poses a significant hurdle. Researchers need a reliable and efficient way to access and analyze protein structures. The current work addresses this issue by developing a user‐friendly website with an integrated vector database for optimized search performance, thereby facilitating easy access and analysis. This study looks into murburn concept from ML perspective by investigating the presence of learnable discriminatory features in the structural descriptions of the proteins. Formulated as a problem of classification into murzymes and classical enzymes, the study would be a validation of murburn concept. It also lays the foundations for a prospective tool for analysis of this class of proteins, which can aid in the discovery/recognition of hitherto unknown murzymes. Therefore, in the third part, hundreds of proteins were labeled and trained as murzymes or classical enzymes, with respect to the attributes described in the earlier methods, as derived from the Protein Data Bank (PDB) file descriptions. The logic thereby was used to parse unknown random proteins from the RSC protein databank. By employing a support vector machine (SVM) model [40], we aim to accurately classify the new “test” molecules by vectorizing their attributes using a transformers‐based embedding model, and using these vectors as inputs for our classification algorithm. Our goal is to improve the precision and recall of these classifications, which in turn enhances the reliability of subsequent biological analyses and applications. Our study addresses the gaps in AI‐ML analyses of protein‐attributes by creating a potentially searchable database and employing state‐of‐the‐art machine learning techniques to analyze the data. This approach is also aimed at bridging the gap between classical and logical cellular biology. We deployed the dataset using a vector database, QDrant [41], which efficiently handles high‐dimensional vector representations. This database supports fast and accurate similarity searches, enhancing the usability of our website for researchers. Our deployment strategy ensures that the data are both accessible and useful for various analytical tasks. In developing the application, we also focused on creating a user‐friendly interface that delivers quick and relevant results. This development process ensures that our tool meets the practical needs of the research community.

### 2.3. Data Acquisition, Preparation, Processing, Model Training, and Deployment

Meta‐analysis of five heme‐containing enzymes/reaction systems was performed, and it was taken as a source of murzyme function, based on the earlier research publications (data and references from [38]). The enzymes salient of central dogma (particularly, the ones belonging to DNA replication, DNA transcription into mRNA, and mRNA translation into protein) were taken as examples of classical enzymes. Such data was used for further work.

*Part 1:* In the first module (for choosing the most favorable mechanistic model), only eight postulates were compared, as shown in Table 1. In the second internal consistency module for assessing classical enzymes (classical enzymes), four mutually exclusive features and four inadmissible features were considered.

*Part 2:* Several (20) features of a given protein/reaction system were provided as training datasets: 10 numbers of murzymes and classical enzymes (classical enzymes) each.

*Part 3:* The dataset for this section was sourced from the Research Collaboratory for Structural Bioinformatics (RCSB) PDB and consisted of 513 protein structures. One hundred forty‐five of these were annotated manually as murzymes and the rest as classical enzymes (and this was based on the findings of Parts 1 and 2). RCSB (PDB) is a comprehensive repository of experimentally determined three‐dimensional protein structures. The PDB files contain information about the protein structures and their associated functions.

Data cleaning: The initial step involved cleaning the PDB files to remove irrelevant or redundant information. This process included removing unnecessary annotations and standardizing the format of the data. There were a total of 513 datapoints, out of which 145 were murzymes and 368 were classical enzymes.

Feature extraction: Relevant textual features such as Compound Names and Key‐Words were extracted from the cleaned PDB files. The features were selected based on their potential bearing on the functional capacity of the proteins to act as murzymes.

Vectorization: The extracted features were then vectorized using a transformers‐based embedding model, BAAI/bge‐small‐en installed from the Hugging‐Face platform.

Data balancing and splitting: Imbalance between the two classes in the data was addressed through over‐ and under‐sampling techniques. The data were randomly split into training and test sets according to a 4:1 ratio.

We selected the relevant experimental, theoretical, and structural features based on available information in literature. Therefore, there is no further scope for preprocessing and feature extraction in Parts 1 and 2. In the context of this work, the logic is to algorithmically verify the murburn postulates (which are well established with forthright interpretations from prior data and theorizations), and not to finetune accuracy based on feature selection. Other aspects that are derived from the basic premises described above are dealt within the sections that follow.

## 3. Results and Discussion

At the outset, once again we state clearly that we do not downplay affinity‐based complexations or ligand‐binding induced formation changes in any/all physiological or in vitro scenarios/contexts, as there are several such instances! For example, even in peroxidases and P450 realms, CYP2D6 mediated enantioselective conversion of bufuralol is definitely a classical active site reaction, where high concentrations of the favorably oriented substrate molecule (with topographically accessible moiety) can approach the Compound I BROS formed at the distal heme pocket. However, we have repeatedly stressed that to use such rare instances (or the results obtained with high‐mM levels of substrates and high‐micromolar levels of proteins, as in crystallographic or synthetic or spectroscopic sample preparations) as salient examples of heme enzyme catalysis is erroneous [39, 42]; as physiological conditions are quite distinct/different. Herein, we highlight our claim (established through decades of systematic explorations) that DROS‐functionalism provides a more thermodynamically, kinetically, and mechanistically viable basis for explaining several/most in situ/in vitro metabolic reactions and physiological outcomes featuring heme enzymes. Under this light, this work was designed to demonstrate the murburn postulate that DRS serve as the quintessential stochastic foundation of instantaneous cellular functions (operating in conjunction with the long‐term regulations exerted by the deterministic actions of genes, by virtue of a specifically designed active site). The hallmark of the murburn paradigm is that it is essentially minimalistic: (a) does not solicit high‐fidelity complexations (protein–protein or protein‐ligand) or sophisticated conformation‐linked affinity/activity alterations, and yet, (b) explains promiscuity of partnering and diversities of proteins/substrates/ligands across systems, idiosyncrasies, atypical kinetics, unusual dose responses, variable stoichiometries, fluctuations of electrical activity, and so on. The salient differences between active‐site catalysis and murzyme catalysis are delineated in Table 3.

**Table 3:** Criteria‐based mechanistic distinctions between classical enzymes and murzymes.

	**Criteria**	**Active-site catalysis (MM)**	**DRS-catalysis (Murburn)**
1	*Key operative*	Physical adsorption or coordination by topological complementation	Electro‐statics/dynamics or ECS‐DRS (effective charge separation and diffusible reactive species)

2	*Locus*	Active site	Delocalized

3	*Intermediation*	High‐affinity transition state complex	Transient collisions and interactive equilibriums (discrete affinity‐bindings not disallowed!)

4	*Selectivity/specificity*	Uniqueness	Diversity

5	*Stoichiometry (reactants: products)*	Integral, definitive	Nonintegral, variable

6	*Molecularity*	Bi‐ to Multi‐molecular	Bimolecular (primarily)

7	*Steps*	Serial‐Sequential	Parallel‐unordered

8	*Pathway (routing)*	Unique	Multiple

9	*Protein-level structural change*	Lock‐key/Induced‐fit	Explainable as is

10	*Thermodynamics*	Endergonic/Exergonic	Exergonic

11	*Temporal frame*	Long‐term	Short‐term

12	*Structure-architecture*	Sophisticated arrangements/dictates	Randomized distribution/mandates

13	*Control/fate*	Deterministic	Stochastic

14	*Modulation*	Allosteric binding	Reactant/catalyst

15	*Probability/evolvability/parsimony*	Complex	Simple

This is the first systematic work of its kind (with the agenda of identifying and classifying murzymes and murburn mechanism) and the results are presented briefly in the main text (and elaborated in the Supporting Information file (available here)) and can be checked out directly at the pertinent website for any protein/metabolic system.

### 3.1. Selected Murburn Systems

First, we performed a meta‐analysis of various components of the five diverse chosen heme enzyme systems, comparing them in various criteria. The results are compiled below under eight minimal heads, making the distinction between (a) classical and (b) murburn models.

**Figure 2:**
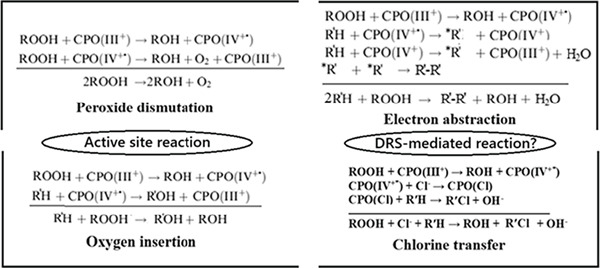
Classical mechanistic cycles of heme‐haloperoxidases: The hydroperoxide dismutations and oxygen insertion reactions may occur at the active site via Compound I, but we are quite assured that the electron abstraction and halide transfer reactions are mediated by DRS, quite unlike the classical mechanism advocates assume. Peroxide consumption in the overall reaction is not just via active site reactions, but it can be facilitated by multiple outside the active site “murburn” reactions, involving other DRS and intermediates produced in situ.

1. *The soluble enzyme system of heme*‐*haloperoxidases* (also catalase; simple enzymes, one active site): Figure 2 and Table 4 show the similarities and differences between the classical and murburn perspectives.

2. *Synthesis of pain-hormones by membrane-embedded cyclooxygenase* (with two active sites?): Box 1 is an elaborate representation of the step‐wise mass‐charge balanced steps of the classical COX reaction (both COX1 and COX2). These isozymes, from diverse mammalian sources, act on various unsaturated fatty acids, to give diverse pain‐hormones like prostaglandins, prostacyclins, and leukotrienes [42]. Table 5 shows the mechanistic comparison.

3. *Endoplasmic reticular drug/xenobiotic metabolism (in liver) by tri-protein system*: Hundreds of CYP isozymes present at high concentrations, a unique diflavoenzyme CYP‐reductase, or CPR at significantly lower concentrations, and copious amounts of cytochrome *b*_5_ constitute membrane embedded multiprotein system that metabolize the bevy of drugs/xenobiotics that enter the body [39]. Table 6 makes comparisons between the two schools of thought, regarding the working mechanism of this system.

4. *Mitochondrial oxidative phosphorylation* (chemical energy currency production or ATP synthesis in all cells) by multiprotein complexes working in tandem: Hundreds of immobilized proteins, assembled into five major complexes, in conjunction with some small molecules (like coenzyme Q) and mobile proteins (like cytochrome C), synergize to make ATP. Table 7 shows the comparison of the two schools of thought.

5. *O_2_ transport and ATP synthesis mediated by hemoglobin in erythrocytes*: The classical theorization has no explanation for Hb‐mediated ATP synthesis and sees it only as an agent for gas transport. Table 8 shows a listing of features of the two schools of hemoglobin function. Only murburn concept explains for the 3‐4 months′ viability of RBC sans nucleus and mitochondria.

**Box 1: The elaborate mechanistic orchestration of COX enzyme, as per the classical perception.**



**Table 4:** Comparison of heme‐haloperoxidase function under two models.

***No.***	***Criteria***	**(a) Lock and key**	**(b) Murburn**
*1*	*Overview*	Heme enzyme	
*2*	*Catalysts/aids*	CPO/catalase/HRP	
*3*	*Reactants*	H_2_O_2_ ± RH ± X^-^	H_2_O_2_ ± O_2_ ± RH ± X^-^
*4*	*Products*	H_2_O(+O_2_) R ^∗^^+^/ROH/RX	H_2_O(+O_2_) R ^∗^^+^/ROH/RX/ROX/HXO
*5*	*Molecularity of the transient species that forms products*	2 ((Comp. I + RH))	2 ((BROS + RH)) 2 ((DROS + RH))
*6*	*Minimal steps in the overall process*	Diffusion, binding and serial reaction of three reactants at active site.	Parallel collisions among DROS/BROS and two reactants in milieu.
*7*	*Substrate/partner affinity and diversity and reaction outcomes*	Diverse peroxides, diverse (in)organics, various halide anions, and diverse products	
*8*	*Relative probability index*	1/8	> 1/8 (multiloci, more modes)

**Table 5:** Comparison of cyclooxygenase mechanism under two purviews.

***No.***	***Criteria***	**(a) Janus**	**(b) Murburn**
*1*	*Overview*	Heme enzyme system (+ Tyr)	
*2*	*Catalysts/aids*	COX, lipid membrane	
*3*	*Reactants*	H_2_O_2_ + AH + AA + O_2_	H_2_O_2_/^1^O_2_/^3^O_2_/O_2_∗^−^/OH∗ + AA
*4*	*Products*	H_2_O + A∗ + AOOAO_2_	H_2_O + A(O_2_)_n_A
*5*	*Molecularity of the transient species that forms products*	3 ((COX + FA+3O_2_))	2 ((FA + DROS))
*6*	*Minimal steps in the overall process*	> 15 serial‐sequential steps	< 5 parallel steps
*7*	*Substrate/partner affinity and diversity and reaction outcomes*	Diverse fatty acids, diverse products	
*8*	*Relative probability index*	1/576	> 1/16

**Table 6:** Comparing the classical and murzyme models of drug metabolism by CYPs.

***No.***	***Criteria***	**(a) P450cam**	**(b) Murburn**
*1*	*Overview*	Heme‐flavin enzyme system	
*2*	*Catalysts/aids*	CYP, CPR, Cyt. *b*_5_, Lipid membrane	
*3*	*Reactants*	RH + O_2_ + NADPH	RH + O_2_ + NADPH
*4*	*Products*	ROH + NADP^+^ + H_2_O	ROH + NADP^+^ + OH^-^
*5*	*Molecularity of the transient species that forms products*	~5 ((Membrane‐[RH‐CYP‐O_2_]‐[CPR‐NADPH]))	2 ((RH‐DROS))
*6*	*Minimal steps in the overall process*	Five sequential and serial binding steps ((5))	Four unordered and parallel interactions
*7*	*Substrate/partner affinity and diversity and reaction outcomes*	Diverse CYPs, diverse DROS, diverse RH, and diverse products	
*8*	*Relative probability index*	<<1/160 ( = 1/5 × 1/32)	> 1/16

**Table 7:** Comparison with the Mitchellian and murburn model of oxidative phosphorylation.

***No.***	***Criteria***	**(a) ETC-CRAS**	**(b) Murburn**
*1*	*Overview*	Heme‐flavin‐FeS enzyme system	
*2*	*Catalysts/aids*	Complexes I‐V, CoQ, Cyt. *c*, Lipid membrane	
*3*	*Reactants*	(i) NADH + O_2_ (ii) 2.5 ADPOH +2.5 POH	NADH + H^+^ + O_2_ + >3 ADPOH + >3 POH
*4*	*Products*	(i) NAD^+^ + H_2_O (ii) 2.5 ATP+2.5 H_2_O	NAD^+^ + H_2_O_2_ + >3 ATP + >3 H_2_O
*5*	*Molecularity of the transient catalytic species*	Innumerable non‐existent protons for recycle +4 stationary multimeric complexes with numerous redox centers +2 mobile e‐carriers +1 multi‐molecular rotary protein +3 phases (two controlled aqueous phases separated by a lipid phase)	Redox protein(s) transiently interacting with diverse components, capable of ECS at lipid interphase
*6*	*Minimal process*	Overall, dozens of serial/sequential binding steps; sometimes even at one protein complex (e.g., Q‐cycle at Complex III)	Unordered and parallel interactions at each protein
*7*	*Substrate/partner affinity and diversity and reaction outcomes*	Diverse e‐donating/e‐accepting reactions in proton‐limited microenvironment	
*8*	*Relative probability index*	Infinitesimal!	Significant and much higher!

**Table 8:** Hemoglobin′s functioning in classical and murburn paradigms.

***No.***	***Criteria***	**(a) Classical O**_**2**_ **transport**	**(b) Murburn**
*1*	*Overview*	Tetrameric hemeprotein binding O_2_	
*2*	*Catalysts/aids/modulators*	Bis‐phosphoglycerate	
*3*	*Reactants*	Nil	ADP + Pi
*4*	*Products*	Nil	ATP
*5*	*Molecularity of the transient catalytic species*	Fe(II)‐O_2_	Fe(II)‐O_2_ or Fe(III)‐O_2_^∗^^-^
*6*	*Minimal process*	O_2_ binding and detachment	O_2_^∗^^-^/O_2_ and ADP binding
*7*	*Substrate/partner affinity and diversity and reaction outcomes*	Not relevant	Could potentially mediate reactions!
*8*	*Relative probability index*	Little scope for consideration of roles other than oxygen‐transport	Experimentally supported theory for RBC viability

### 3.2. Positive Controls for High‐Affinity Transition‐State–Mediated Enzyme Systems

Classical genetic or metabolic reactions often involve the formation of multiprotein complexes to carry out the catalysis efficiently, and they involve both serial and parallel processes. The salient examples (DNA replication, RNA transcription, protein translation, DNA repair, proteasome machinery. NRPS, amylase, lipase ligase, and aldolase), and so on, all of which have high‐fidelity processes, with very specific substrate and products, formed in predictable stoichiometry. The details of these systems are given in Supporting Information.

### 3.3. Algorithmic Analyses of Various Enzymes/Systems

In Part 1, the simple algorithm consistently favored the murburn postulates over the active‐site–based proposals and the latter was demonstrated to have internal conflicts in several redox enzyme systems. Also, the mutual exclusiveness of certain postulates or/and experimental observations clearly rule out the relevance of classical active‐site binding enzyme mechanism in several heme‐enzyme reactions. As per the demarcation exercise of Part 1 above, a total of 37 enzymes were parsed into the two known classes of murzymes (Numbering 22) and classical enzymes (Numbering 15). In Part 2, When 17 (unused for training; five classical enzymes, and 12 murzymes) proteins/systems were given for parsing, all of them were identified correctly by both decision tree and random forest AI‐ML methods, in spite of the low amount of training data. To evaluate the predictive significance of various biochemical feature categories in distinguishing murburn‐type and classical‐type enzymatic systems, decision tree classifiers were trained using category‐wise features from the dataset. Each model was evaluated using 20 randomized train‐test splits (60% training and 40% testing), and average classification accuracy was recorded. The experimental features yielded maximal achievable classification performance, indicating that features like substrate specificity and non‐integral stoichiometry alone are sufficient and highly discriminative to distinguish between murburn and classical systems. Theoretical features also demonstrated strong discriminative power, with average accuracy = 1. Structural features were also predictive, giving average accuracy: of 0.9667 across splits. Average classification accuracy, precision and recall for each category of features is presented in Table 9. The Supporting Information file also gives information gain of each feature. Various structural, theoretical, and experimental features of an enzyme/system were found to impact the classification, with the structural component of heme′s presence, theoretical aspect of DRS involvement and experimental components of variable/nonintegral stoichiometry and nonspecificity/selectivity being important indicators deciding favorably for murburn model. The trends for 20 (+8 postulate) features explored in this study are listed in Table 10. These trends were further used as “rules” for affording a flexible parsing algorithm (used at the website), which could predict the functional class (murzyme or nonmurzyme) with various levels of confidence, depending upon the type and extent of data entered at the user interface.

**Table 9:** Average performance using different feature categories for the Part 2 decision tree classification.

**Category**	**Accuracy**	**Precision (Classical)**	**Precision (Murzyme)**	**Recall (Classical)**	**Recall (Murzyme)**	**F1 score (Classical)**	**F1 score (Murzyme)**
Structural	0.97	0.91	1.0000	1.0000	0.95	0.95	0.97
Theoretical	1.0000	1.0000	1.0000	1.0000	1.0000	1.0000	1.0000
Experimental	1.0000	1.0000	1.0000	1.0000	1.0000	1.0000	1.0000

**Table 10:** Trends for classifying proteins/systems into murzyme/murburn category, as ascertained from the first two parts of the current study.

**No.**	**Criteria**	**Feature**	**Trends**	
			**Classical**	**Murburn**
**1**	**Structural**	*Heme?*	No	Yes
**2**		*Flavin?*	Usually no	Some yes
**3**		*FeS?*	Rare	Some yes
**4**		*Constrained access to active site*	No	Yes
**5**		*Substrate larger than active site*	No	Some yes

**6**	**Theoretical**	*Overall redox reaction?*	No	Yes
**7**		*Exergonic?*	Mixed	Yes
**8**		*Oxygen needed?*	Usually no	Usually yes
**9**		*DRS involvement?*	Usually no	Yes
**10**		*Reversible?*	Yes	Need not be

**11**	**Experimental**	*Selectivity for substrates?*	Yes	No
**12**		*Specificity of products?*	Yes	No
**13**		*Diversity of modulators?*	No	Yes
**14**		*Non-integral stoichiometry?*	No	Yes
**15**		*Variable stoichiometry?*	No	Yes
**16**		*Unusual values? (* *K*_*M*_ < *K*_*d*_ *; high Int. KIE)*	No	Yes
**17**		*Catalytic rate exceeding diffusion limits?*	No	Yes
**18**		*Atypical substrate dependence?*	No	Yes
**19**		*Bulk-phase (ionic/dielectric) dependency?*	No	Yes
**20**		*Atypical temperature dependence?*	No	Yes

**21**	**Postulates**	*Enzyme shape-change needed?*	Sometimes	No
22		*Multisubstrate Serial/Sequential?*	Yes	No
23		*Complex requirements?*	Sometimes	No
24		*Molecularity of key Intermediates?*	Sometimes high	Low
25		*Number of mechanistic steps?*	Sometimes high	Moderate
26		*Probability index?*	Low	Moderate
27		*Long distance OS-ET?*	Sometimes	No
28		*High affinity E + S complex?*	Yes	Usually no

We envisaged that such structural/experimental/theoretical and postulate details may not be available in real‐world scenarios, and it should be facile to categorize a protein based on the attributes given to it in the PDB description itself. This was undertaken in Part 3, whose results follow. The performance evaluation of the machine‐learning models, SVM, random forest and logistic regression, trained on a dataset of PDB files to classify proteins as either murzymes or classical enzymes. The dataset was split into 80% training and 20% testing sets. To address the class imbalance observed in the initial dataset, an oversampling technique was applied during the training phase. After class balancing, the performance of both models significantly improved. The SVM model achieved an accuracy of 84.3% (Table 11), exhibiting a substantial increase compared to its performance on the imbalanced dataset. The key findings are:

**Table 11:** Prediction results of Part 3 AI‐ML exercises to identify murzymes from PDB annotations.

**Metric**	**Logistic regression**	**SVM**	**Random forest**
Precision (Murzymes)	0.55	0.81	0.48
Recall (Murzymes)	0.79	0.85	1.00
F1‐score (Murzymes)	0.65	0.83	0.65
Support (Murzymes)	63	63	63
Precision (Classical)	0.68	0.77	0.00
Recall (Classical)	0.41	0.79	0.00
F1‐score (Classical)	0.51	0.78	0.00
Support (Classical)	69	69	69
Accuracy	0.59	0.84	0.48

Class balancing: The results demonstrate the significant impact of class balancing on model performance. Addressing class imbalance improves the model′s ability to correctly identify murzyme proteins, highlighting the importance of using appropriate data preprocessing techniques for imbalanced datasets.

SVM model Pprformance: The SVM model exhibited superior performance compared with logistic regression after class balancing. This indicates that SVM is a more suitable algorithm for classifying murzyme proteins in this specific dataset.

### 3.4. Summary of the Work and Prevailing/Future Prospects

**Figure 3:**
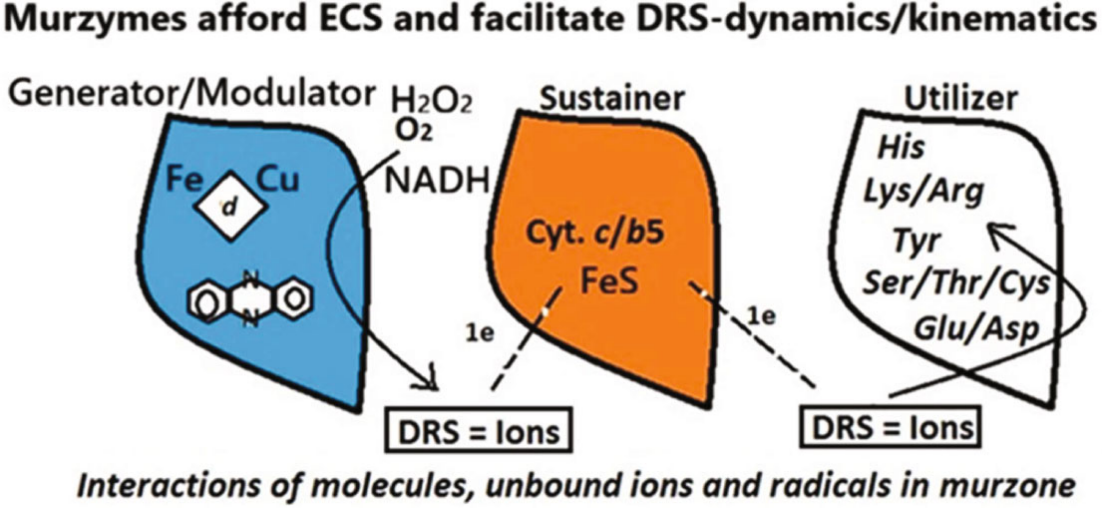
The various types of murzymes and their mechanistic contexts are shown.

A. From the procedures detailed in Methodology section and the meta‐analysis of reaction mechanism first detailed in Results (using comparative scales such as relative probability index), we identified the main features of murzymes and murburn systems in Part 1. In Part 2, the dataset used for classification comprised 39 enzyme samples, of which 22 were categorized as murzymes and 17 as classical enzymes. A total of 20 features were used for analysis, grouped into three categories: experimental, structural, and theoretical. Detailed descriptions of all data samples and feature definitions are provided in the Supporting Information. (This also gives insight on an “information gain” for each feature, which tells about the relevance/ability of the feature to demarcate into the two classes, on its own merit. Higher information gain means the feature strongly contributes to classification.) The data selected were salient systems that are well‐studied and for which decades of experimental, theoretical, and structural aspects were known. The classification gave maximal achievable results, as seen in Table 9. The PDB files were parsed to extract semantic features that were encoded using the transformer model, BAAI/bge‐small‐en into 384 dimensional vectors. The population imbalance in the dataset was mitigated through random oversampling. The transformer embeddings were served as inputs to SVMs that produced an accuracy of 84.3%, a precision of 0.79, and a recall of 0.82 (Table 11). In order to ensure reliability of the results, an 80:20 train‐test split was performed multiple times and the average was computed for the metrics. The website featuring the identification of murzyme/murburn systems employs a simple code (which uses the insights derived from AI‐ML logic) and affords predictability based on detailed or simple inputs. That is, it affords screening on the basis of listing known mechanistic (structural/experimental features) or even PDB text attributes.
B. DRS are well‐reported in biological literature. DROS/DRNS (diffusible reactive oxygen/nitrogen species), like superoxide anion, hydroxyl radical, hydrogen peroxide molecule, nitric oxide radical, and peroxynitrite anion are examples of DRS. The vast majority of cellular/tissue level DRS‐generating activities (except exceptional cases like NO‐signaling) are currently deemed as toxic/wasteful processes, leading to “oxidative stress.” This is because of the long‐standing perception that only enzyme′s active‐site bound reactive oxygen species (BROS, e.g., Compound I) would be expected to afford deterministic selectivity/specificity, in contrast with the potentially mobile (and hence, chaotic!) DROS. Over the past couple of decades, our work leading to murburn concept has challenged this misperception and established DRS as an obligatory/unavoidable mainstay of cellular physiology. Murburn concept advocates that some proteins serve as murzymes, recruiting DRS (Figure 3). Herein, we presented a meta‐analysis of “BROS versus DROS” proposals in the following heme‐protein–mediated processes across diverse cellular loci/realms: (1) peroxidase mediated e‐abstractions and chlorinations in aqueous peroxisomal or extracellular milieu, (2) synthesis of pain hormones by cyclooxygenase at membranes of specific cells, (3) metabolism of diverse xenobiotics by cytochrome P450s or CYPs at the endoplasmic reticulum of hepatocytes, (4) oxidative phosphorylation by mitochondrial and prokaryotic membrane complexes in aerobic cells, and (5) oxygen‐transport coupled ATP‐synthesis by hemoglobin in erythrocytes. To arbiter the BROS‐DROS dispute, we availed Ockham′s razor and internal consistency checks as yardsticks from mechanistic postulates and protein/experimental features, respectively. We also presented AI‐ML based (decision tree, random forest and SVM) differentiation/parsing protocols for murzymes in two scenarios: (i) with the input of known structural/experimental details of the protein, and (ii) text attributes extracted by “LLM‐based” methods from the PDB files of random proteins from RSC databank. For all these modules, we have enabled a globally perusable user‐interface over a freely accessible website and also deposited the Python codes at GitHub.

C. Traditionally, enzymes have been classified into seven classes within the broad mechanistic perspective of the electron‐group transfer and covalent/acid‐base/nucleophilic catalysis (the murzyme mechanistic interpretation is given in braces):
  i. Oxidoreductases (direct redox modulators or DRM),
  ii. Transferases (group transfer and field modulators or GTFM),
  iii. Hydrolases (covalent reconfigurator or CR1),
  iv. Lyases (yet another type of covalent reconfiguration or CR2),
  v. Isomerases (structural regulator or SR),
  vi. Ligases (CR + GTFM), and
  vii. Translocases (diffusion facilitators or DF).

In the physiological context, the murburn perspective would solicit other types of functionalisms also, such as:

a. energy transduction catalysts (or ETC, say converting ionic/redox or light energy into useful work, like Complex V/Na,K‐ATPase or photosystems)
b. coherence modulators (or CM, which bring together the chemoelectromagnetic matrix or CEM framework like rhodopsin in photoreceptors or signaling cascade protein NO‐synthase)

The classical enzymes could also be functionally parsed into types of lytic (peptidases, esterases, glycosidases, and lyases), group‐transfering (methylase and kinase), synthetic (ligases and polymerases), and structural (isomerases, topoisomerases, and chaperones) roles. The criteria for parsing murburn catalysts can also be along: redox murzymes (cytochrome‐*C* and plastocyanin), energetic murzymes (Complex V and Na^+^/K^+^‐ATPase), reactive murzymes (P450s and peroxidases), and field murzymes (photoreceptors and cyclooxygenase). The classifications above reflect energetic modality (redox, field, or covalent coupling), spatial domain (active site, interfacial, or bulk), stochastic mechanism (radical, ionic, or electronic), and functional outcome (chemical, energetic, or informational coherence).

**Figure 4:**
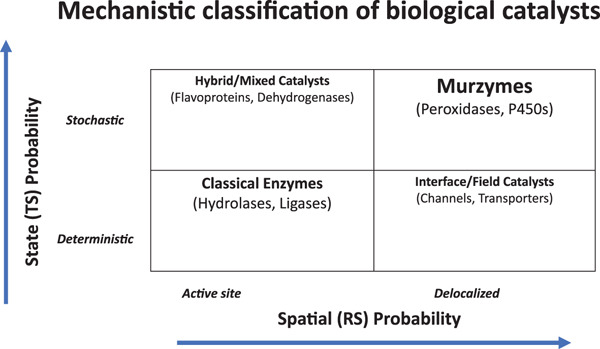
Mechanistic classification of biological catalysts. (TS: transition state, RS: reaction site. Please see text for details) The probability score is lower at the intersection of the axes and increases outward.

**Figure 5:**
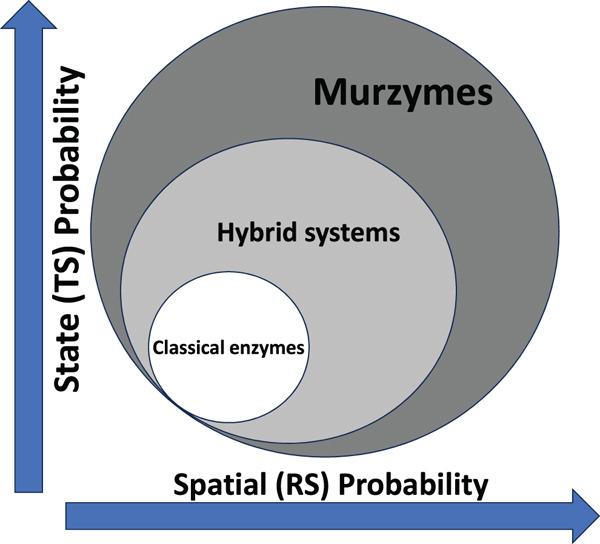
Murzymes are a larger superset of enzymes that also include the classical/canonical enzymes and other intermediary systems (TS: transition state, RS: reaction site). Please see text for details.

Such organization terminologies would obviate the usage of redundant terms like electron transport chains, proton/ion pumps, rotary motors, and so on, which have little grounding with respect to thermodynamic feasibility, kinetic viability, evolution, or architectural/structural realities. In pedagogy, the “apparent dichotomy” of geometry‐bound and field‐spread catalysis can be easily demarcated, with the interpretation that classical enzymes came around later, with refinement and selection over time. (See Figures 4 and 5 for an insight in this regard.) In the integrative continuum within the cellular ambiance, the ideas of murburn, coacervation, and CEM add new whorls/richness to both understanding and adaptation of life mechanisms (in contrast with the merely linear or circular arrays of metabolic networks in classical “dilute solution” biochemistry).

It must be stressed that a murzyme is not a new subclass of enzyme but a broader mechanistic superset encompassing both classical deterministic catalysis and stochastic redox‐field chemistry (Figure 5). Classical enzymes are deterministic specializations within this spectrum. Murzymes represent the foundational energetic continuum that powers and regulates all biochemical catalysis, from which the canonical classical zymes evolved. Theoretically, all seven functions of classical enzymes can also be achieved by murzymes. However, the most salient ones are just cited in Figure 4 to better grasp a typical case scenario. Although the murburn mechanism was classified as generator, modulator, sustainer and utilizer in Figure 3, they can also be seen under the following mechanistic contexts of classification: (1) Mode of energy coupling (e.g., direct redox vs. DRS‐mediated); (2) type of diffusible mediator (e.g., radical, ion, and field); (3) spatial domain of catalysis (allo‐site bound, interface‐mediated, or bulk stochastic); and (4) nature of chemical transformation (e.g., oxidative activation, group rearrangement, and bond recombination).

Perceptional overlap/confusion is avoided if we define the primary catalytic driver as the distinguishing criterion: If catalysis depends on geometric complementarity, bond polarization, and confined transition state, it is a classical enzyme. If catalysis depends on stochastic generation and regulation of DRS via an ECS mechanism, it is a murzyme. Thus, no ambiguity arises as long as we anchor the classification on mechanistic causation rather than mere functional outcome. The advent of murzyme awareness transforms enzyme science from a catalog of molecular machines to a unifying mechanistic vision of the physics of life where energy flow, redox stochasticity, and molecular/systemic coherence coalesce to produce cognition (as an outcome of ET, the fundamental manifestation of electromagnetic force). The classical view was purely chemical (geometric and stoichiometric). In comparison, the murburn‐outlook is more physical (energetic, stochastic, and systemic) and thus amenable to further macroscopic conceptualization. It shifts the focus from “*What bond changes?*” to “*How is energy redistributed and coherence achieved?.*” Such an approach could afford: (i) mechanistic unification (e.g., a term like “stochastic redox‐field catalysis” could obviate nonphysical terms like “pumps and electron carriers”); (ii) energetic realism (thermodynamic plausibility and energy dissipation would be more important than the earlier unreal “vectorial precision”!); (iii) predictive framework (the name could reflect upon its mechanistic intermediates and environmental vulnerability, for example); and (iv) continuity with intelligence models (CMs at CEM level could scale up to cognitive field coherence at the organism level!).

In the murburn purview, enzymes and cellular components are no more rigid molecules independently working on discrete reactions via fixed mechanistic steps/cycles. The protagonists are probabilistic moderators of charge, spin, and energy; thus operating as local dissipative systems. Although the depiction in Figure 4 shows four different boxed areas, the reality is a continuum of murzymes, with various classes having different levels of determinism (or stochasticity). For the reader′s benefit, Table 12 summarizes the salient differences between the classical enzymes and murzymes.

**Table 12:** Salient distinctions between classical enzymes and murzymes (although murzyme is the superset including classical enzyme!).

	

The probability map in Figure 4 underscores that living cells employ both strategies continuously/simultaneously: classical enzymes ensure chemical specificity with controlled covalent transformation and murzymes sustain energetic redox coherence through stochastic field coupling. Together they create a unified biochemical continuum linking deterministic structure‐based catalysis with probabilistic field‐based regulation; the energetic foundation of BI, and also founded/reflected in PCHEMS activities.

The evolutionary and mechanistic trajectory can be visualized as a diagonal flow from murzymes → hybrid systems → classical enzymes, representing increasing spatial localization and decreasing stochasticity (Figure 5). Conversely, in highly integrated modern systems (e.g., mitochondria, chloroplasts, and brain), both classes coexist and interact synergistically, with murzymes providing energetic coherence and classical enzymes providing chemical precision. It is apparent that the standard free energy changes of most metabolic reactions fall at very low values (close to equilibrium; and thus is reversible in physiology with the change of substrate molecules′ concentrations). The vast major biopolymers′ connecting ester‐bond hydrolysis is exergonic because it increases entropy, forms products with stronger bonds (O–H and *C* = *O*) than the ones it breaks, and the abundant water is both a reactant and solvent. For example‐ glycosidic (−5 to −15 kJ/mol), peptidases (−10 to −20 kJ/mol), lipases (−15 to −30 kJ/mol), phosphodiesterases (−20 to −40 kJ/mol), and ATPases (−30 to −50 kJ/mol). Therefore, proto‐anabolic reactions could have employed murburn chemistry for synthetic purposes (which continues even today, for ATP synthesis!), owing to its simplicity and energetic viability. When ECS‐DRS mechanisms operate transiently, they alter charge distribution, change polarity and pH (a micro‐demixing), and can oxidize/cross‐link amphiphilic molecules, all to generate supramolecular associations; thus enhancing coacervation (another prominent aspect neglected in modern biology because the latter extensively employes dilute solution chemistry computations). Coacervates are essentially self‐organized condenstates of reactive and energetic coherence, sustained by murburn. Since 1950s till date, convincing experiments have provided evidence for the role of radical and DRS chemistry supporting the formation of amphiphilic condensates [43, 44], photo/redox‐driven coacervation [45–50] and protocell type functionalization from solution phases in primordial times, to give the CEM in living beings [6]. Therefore, origin‐of‐life and synthetic biology research gain a prebiotic energy paradigm explaining condensation and coacervation without ATP or membranes.

Murzyme chemistry ushers in a total revamp in bioenergetics and redox enzymology. It helps reinterpret enzyme mechanisms, explaining anomalies in important systems like P450s, cyclooxygenase, Complex V or ATP synthase, and other mitochondrial complexes through stochastic energy coupling rather than mechanical or proton‐pump models. In biochemistry education and modeling, it bridges deterministic kinetics with open‐system thermodynamics, grounding life processes in physical reality. In the field of synthetic chemistry, murzymic catalysis inspires mild, green oxidation, hydroxylation, and coupling reactions using DRS fluxes instead of harsh reagents. It suggests redox‐field‐mediated polymerization, electrocatalysis, and photochemical systems operating under ambient conditions. In pharmacology, it reframes reactive oxygen species as useful and regulatory elixirs rather than purely destructive agents, guiding new insights into metabolic modulators; giving research reorientations in diverse medical‐health science fields. Systems biology can adopt this view to model metabolism as interconnected redox‐field domains instead of linear pathways. Overall, murzyme awareness integrates energy, structure, and function across biology and chemistry; linking stochastic field coherence with enzymatic action.

D. Murburn concept‐based approach offers a novel perspective on functional mechanisms employing proteins, challenging and complementing/supplementing established paradigms in cellular biology. From a physicochemical perspective, in heme proteins, the porphyrin pi‐electron cloud conjugated central heme Fe atom could be seen as an “electron‐sensitive” protagonist that would be subject to perturbations by varying environmental ambiances. The most common oxidation states of Fe is 2^+^ (II) or 3^+^ (III), although higher states have also been characterized. In presence of oxygen, peroxides or reductants (like NADH/NADPH) or electron‐active suitable chemicals, the heme Fe system is known to manifest the generation of DRS like superoxide, singlet oxygen, peroxide and hydroxyl radical. Quite simply, the Fe(III)‐O_2_^∗^^-^ species is electronically equivalent to Fe(II)‐O_2_; therefore, it is merely a matter of statistical distribution as to whether oxygen (singlet or triplet) or superoxide dissociates from the heme center. Peroxide could spontaneously or catalytically dismutate into peroxide, and their auto/catalyzed reactions could give hydroxyl radical in milieu. Therefore, wishing away DRS with nonphysiological significance is unwarranted, both theoretically and observationally; and deeming it only pathophysiological significance also falls short of explaining reality. Particularly, when we see that a given heme‐enzyme could catalyze the conversions of diverse substrates and show non‐Michaelis kinetics and nonintegral/variable stoichiometry! As seen from the fifth system (hemoglobin in erythrocytes) explored here, the classical approaches cannot explain the tetrameric structure (with two alpha and two beta dimers) structure of Hb and the classical approach also fails to address the systemic bioenergetics, whereas murburn approach solves both [51]! DROS have been typically seen as disruptors of life and recently, deep‐learning framework was reported for identification of ROS producing enzymes in microorganisms [52]. This study employs fundamental biochemical information and algorithmic procedures, including AI‐ML–based techniques to classify proteins (as availed in detail with mechanistic information or merely as PDB file introductory attributes) into murzymes and classical enzymes. From the first and second modules of this work, the features of DRS‐involvement and nonintegral (and variable) stoichiometry and presence of heme were found to be crucial for the protein system′s classification into murzymes.

Currently, major drug metabolism researchers and leading scientists [26, 53] continue to disregard the dozens of anti‐Michaelis–Menten arguments for xenobiotic metabolism that we presented [39]. We believe that such an outlook may disadvantage the general population, academic interests, and pharmaceutical industry. Hopefully, the first two parts of this work would bring some reorientation in the community and usher in the realization that these heme‐protein systems generate, utilize, modulate, and stabilize DRS! In the last part of the work, by extracting and vectorizing relevant textual features using a transformers‐based embedding model, we achieved precise classification outcomes. The integration of data preprocessing and balancing techniques significantly enhanced the model′s performance, achieving an accuracy of 84.3% for the SVM model post‐class balancing. This highlights the critical importance of addressing data imbalance in biological datasets to improve the robustness of machine learning models. Our research not only supports murburn concept but also demonstrates the efficacy of algorithmic methods for overcoming strictures offered by enforced consensus and advancing the global understanding of rapid cellular biochemical processes. By developing a user‐friendly platform with an integrated vector database, we have facilitated efficient access and analysis of murburn molecules (as PDB files). Our work stands out due to its comprehensive approach to analyzing murburn systems, combining feature extraction, embedding generation, and machine‐learning classification in one framework. By integrating these elements, we provide a robust tool for researchers to explore and understand murzymes better. This research not only bridges the gap between classical and logical cellular biology but also holds potential for enhancing drug design and therapeutic strategies by deepening our understanding of protein functions. The user‐friendly interface design ensures that both novice and experienced researchers can navigate the platform with ease. Although the SVM model demonstrates promising results, our ongoing efforts further explore the use of Convolutional/Graphic Neural Networks (CNNs/GNNs) for murzyme classification, using the structural details of the proteins (as available in the PDB file coordinates and not just its textual annotation) and substrate molecules. This research is poised to provide valuable insights into the field of mechanistic enzymology (maverick modulations, both activations and inhibitions!), protein‐function analysis and contribute to the development of novel pharmacodynamics/pharmacokinetic approaches; for example‐ how an enzyme like cyclooxygenase could be inhibited by molecules with diverse topologies and reactive groups is better explained by the murburn model [42].

### 3.5. Websites and Public Repositories

Details of murburn concept can be availed from http://www.satyamjayatu.com and lectures (with slides) on the topic can be accessed at: https://www.youtube.com/@satyamjayatu5613 . Some other trivia and the parsing exercises on murburn/murzyme done in this work can be freely accessed/executed online at: http://103.10.27.12:8000. All programs/codes used in this study have been deposited at GitHub public repository: https://github.com/Murburn-Labs/Murburn-Snippets

**Nomenclature**
ATP: Adenosine triphosphate
BROS: Bound Reactive Oxygen Species
CAT: Catalase
CCP: Cytochrome c peroxidase
CEM: Chemico‐electromagnetic matrix
CM: Coherence modulator
COX: Cyclooxygenase
CPO: Chloroperoxidase
CPR: Cytochrome P450 reductase
CR1: Covalent reconfigurator (type 1)
CR2: Covalent reconfigurator (type 2)
CYP: Cytochrome P450
DF: Diffusion facilitator
DNA: Deoxyribonucleic acid.
DRM: Direct redox modulator
DR(O)S: Diffusible Reactive (Oxygen) Species
DRS: Diffusible Reactive Species
DRNS: Diffusible Reactive Nitrogen Species
ECS: Effective charge separation
ET: Electron transfer
ETC: Energy transduction catalyst (or Electron transport chain, context‐dependent)
FA: Fatty acid
GTFM: Group transfer and field modulator
Hb: Hemoglobin
HRP: Horseradish peroxidase
KIE: Kinetic isotope effect
LLM: Large language model
Mb: Myoglobin
ML: Machine learning
MM: Michaelis–Menten
MPO: Myeloperoxidase
mRNA: Messenger RNA
NAD(P)H: Nicotinamide adenine dinucleotide (phosphate) reduced form
NRPS: Nonribosomal peptide synthetase
OS‐ET: Outer sphere electron transfer
PCHEMS: Powering, Coherence, Homeostasis, Electro‐mechanical activities, Sensing‐response
PDB: Protein Data Bank
Pi: Inorganic phosphate
RBC: Red blood cell
RCSB: Research Collaboratory for Structural Bioinformatics
RH: Substrate (organic molecule)
RNA: Ribonucleic acid
ROH: Alcohol product
ROS: Reactive oxygen species
RS: Reaction site
SCE: Simple chemical engine
SR: Structural regulator
SVM: Support vector machine
TKM: Thermodynamic‐kinetic‐mechanistic
TS: Transition state

## Consent

The authors have nothing to report.

## Conflicts of Interest

The authors declare no conflicts of interest.

## Author Contributions

K.M.M. conceived the work and wrote the first draft of the paper and all others helped in writing and proofing of the manuscript. V.R. executed the first part of this work (algorithmic preference based on Ockham′s razor and internal consistency) and guided the third part (LLM‐ and SVM‐based parsing of enzymes/systems conducted by A.S. and P.O.R), U.P. helped out in the second part of decision‐tree based classification work, K.P.S. mooted the necessity of algorithmic foundations of murburn mechanism and provided key references/inputs/corrections.

## Funding

No funding was received for this manuscript.

## Supporting information


**Supporting Information** Additional supporting information can be found online in the Supporting Information section. The details of proteins and codes have been given as supporting information.

## Data Availability

The data that support the findings of this study are available in the supporting information of this article.
