# Supplementary material for: Algorithmic Identification of Murzymes and Murburn Mechanisms Based on Structural, Theoretical, Experimental, and Generic Features
Source: Biomed Res Int. 2026 Mar 10;2026:2577941. doi: 10.1155/bmri/2577941 (PMC12975639; doi:10.1155/bmri/2577941)
Supplement: Supplementary file 1 — Supporting Information Additional supporting information can be found online in the Supporting Information section. The details of proteins and codes have been given as supporting information. [file BMRI-2026-2577941-s001.docx]

**Supplementary Information**

**(Identifying murzymes, Manoj et al., Sep 2025)**

**Part 2 Proteins/Systems details**

**Details of System 1, soluble peroxidase/catalase**

For details of the mechanistic/kinetic/stoichiometric interactions, please refer Figure xx of Manoj et al., Biochimie 2016.

**Heme enzymes (10 examples):** Proteins like chloroperoxidase (CPO from *Caldariomyces fumago*), horseradish peroxidase (HRP), myeloperoxidase (MPO), cytochrome c peroxidase (CCP), catalase (human, bovine liver, E. coli, etc.), lignin peroxidase (LiP from fungi), lactoperoxidase (LPO), catalase-peroxidase (KatG, from diverse microbial sources), alkaline peroxidase (APO), thyroid peroxidase (TPO), etc. can catalyze electron abstraction from a diverse array of molecules and insert halogen atoms into the same.

**Substrates**: hydrogen peroxide, organic hydroperoxides (methyl, ethyl, t-butyl, etc.), diverse phenolics (pyrogallol), aromatic amines (TMPD), heterocyclics, indole, beta-phenylethylamine, ABTS, olefines, beta-diketones, sulfides, thioureas, etc.

**Inhibitors/modulators**: diverse substrates themselves, heavy metal ions (copper cations, such as those in CuSO_4_), cyanide, azide, aminotriazole, ascorbate, hydrogen sulfide, hydroxylamine, dipotassium trioxohydroxytetrafluorotriborate (K_2_[B_3_O_3_F_4_OH]), etc.

**Details of System 2 COX**

Several fatty acids with varying chain lengths, number of cis-double bonds starting at differing carbon atom number (starting from the non-carboxy end!) like: arachidonic acid [AA 20:4 (n-6)], eicosapentanoic acid [EPA 20:5 (n-3)], dihomo-gamma-linolenic acid [DGLA 20:3 (n-6)], alpha linolenic acid [ALA 18:3 (n-3)], linoleic acid [LA 18:2 (n-6)], mead acid [MA 20:3 (n-9], docosahexanoic acid [DHA 22:6 (n-3)] are metabolised by COX enzymes.

Many ROS like peroxide, superoxide and singlet oxygen are known to be involved with COX activity.

### COX are known to give diverse array of products, belonging to leukotriene, prostaglandin, prostacyclin, etc. classes.

**Details of System 3, CYPs**

Various liver CYP isozymes can metabolize diverse drugs and xenobiotics.

| **Isozyme** | **Classical substrates and reactions** | **Inhibitors** | **Inducers** |
| --- | --- | --- | --- |
| CYP1A2 | 7-ethoxyresorufin-O-deethylation, phenacetin O-deethylation (caffeine, tizanidine) | α-naphthoflavone, furafylline, fluvoxamine | Omeprazole |
| CYP2B6 | bupropion hydroxylation, efavirenz hydroxylation (methadone, cyclophosphamide) | clopidogrel, sertraline, thiotepa, ticlopidine | phenobarbital, rifampicin |
| CYP2C8 | amodiaquine N-deethylation, paclitaxel 6α-hydroxylation (repaglinide) | gemfibrozil glucuronide, montelukast, phenelzine, clopidogrel | Rifampicin |
| CYP2C9 | diclofenac 4'-hydroxylation, S-warfarin 7-hydroxylation (tolbutamide) | sulfaphenazole, tienilic acid, fluconazole | Rifampicin |
| CYP2C19 | S-mephenytoin 4'-hydroxylation (lansoprazole, omeprazole) | N-3-benzyl-nirvanol, loratadine, nootkatone, ticlopidine, fluvoxamine | Rifampicin |
| CYP2D6 | bufuralol 1'-hydroxylation, dextromethorphan O-demethylation (desipramine, nebivolol, debrisoquine, sparteine, codeine, metoprolol, tamoxifen) | paroxetine, quinidine, fluvoxetine, mirabegron, bepridil, oritavancin | Corticosterone |
| CYP2E1 | chlorzoxazone 6-hydroxylation, nitrophenol hydroxylation (ethanol, acetone, fatty acids, acetaminophen, carbon tetrachloride, benzene) | 4-methylpyrazole, indazole, diethyldithiocarbamate, disulfiram | Ethanol |
| CYP3A4 | midazolam 1'-hydroxylation, testosterone 6β-hydroxylation (triazolam, alprazolam, statins, buspirone, amlodipine) | azamulin, itraconazole, ketoconazole, clathromycin, troleandomycin, verapamil, erythromycin, flucoaozole | rifampicin, carbamazepine, phenytoin |

<https://www.fda.gov/drugs/drug-interactions-labeling/drug-development-and-drug-interactions-table-substrates-inhibitors-and-inducers>

**15 systems of high affinity interactions**

**1. DNA replication machinery**: DNA replication involves the duplication of the genetic material in cells prior to cell division. ***Proteins involved include-*** **Initiation Proteins: DNA helicase, single-stranded binding proteins (SSBs), origin recognition complex (ORC); Elongation Proteins: (RNA) primase, DNA polymerase**, sliding clamp, clamp loader; **Strand Termination and Processing: RNase H, Flap endonuclease 1 (FEN1), DNA ligase; Enzymes for Supercoiling and Strand Tension: Gyrase/Toposisomerase. Reactants:** **DNA Template, Nucleotides (dATP, dGTP, dTTP, dCTP), RNA Primers,** ATP for energy, **Magnesium Ions (Mg²⁺); Products:** Two daughter complementary DNA strands, bound to one parent strand each.

**2. RNA transcription machinery:** Transcription involves synthesizing RNA from a DNA template, serving as the first step in gene expression. ***Proteins involved include****-* Initiation Proteins: RNA polymerase, transcription factors (e.g., TBP, TFIIB), mediator complex; Elongation Proteins: RNA polymerase, elongation factors (e.g., SPT5, P-TEFb); Termination Proteins: Termination factors (e.g., Rho factor or polyadenylation factors in eukaryotes); Regulatory Proteins: Enhancers, silencers, and chromatin remodelers (specific to eukaryotes);

**Reactants**: DNA Template (coding strand), Ribonucleotides (ATP, UTP, CTP, GTP), Magnesium Ions (Mg²⁺) as cofactors for RNA polymerase activity, ATP and GTP for energy. **Products**: Messenger RNA (mRNA), Ribosomal RNA (rRNA), or Transfer RNA (tRNA) depending on the gene transcribed.

**3. Protein Translation Machinery:** Translation involves decoding mRNA into a polypeptide chain, forming functional proteins. ***Proteins involved include***- Initiation Proteins: Initiation factors (e.g., IF1, IF2, IF3 and eIFs), ribosome subunits (30S/50S and 40S/60S). Elongation Proteins: Elongation factors (e.g., EF-Tu, EF-G and eEF1, eEF2). Termination Proteins: Release factors (e.g., RF1, RF2 and eRF1, eRF3). **Reactants**: mRNA Template, Aminoacyl-tRNAs (tRNAs linked to their specific amino acids), Ribosomes, GTP for energy. **Products**: Polypeptide chains (primary structure of proteins).

**4. DNA repair machinery**: Several enzymes complex to effect very high affinity binding-based outcomes for repairing bases or nucleotides missing. ***Proteins involved include:***  (i) Base excision repair (BER): Glycosylases, AP endonucleases, DNA polymerase, DNA ligase AND (ii) Nucleotide excision repair (NER): Excision nucleases, helicases, polymerases [UvrA, UvrB, UvrC (prokaryotes) or XPA, RPA, TFIIH, ERCC1-XPF (eukaryotes)]. **Reactants**: Damaged DNA and pertinent bases/nucleotides. **Products:** Repaired DNA.

**5. Proteasome Machinery:** The proteasome is a multi-protein complex responsible for degrading damaged, misfolded, or unneeded proteins into smaller peptides through a highly regulated process. ***Proteins involved include:*** Core Components- 20S Core Particle (α and β subunits) responsible for proteolytic activity. Regulatory Components- 19S Regulatory Particle: Recognizes ubiquitinated proteins, unfolds them, and translocates them to the 20S core. Ubiquitin-Activating Enzyme (E1), Ubiquitin-Conjugating Enzyme (E2), Ubiquitin Ligase (E3): Add ubiquitin to target proteins for degradation, Deubiquitinating Enzymes (DUBs): Remove ubiquitin chains before degradation. **Reactants:** Ubiquitinated proteins (substrates), ATP for energy (used in protein unfolding and translocation). **Products:** Small peptide fragments, Free ubiquitin molecules (recycled).

**6. Pyruvate Dehydrogenase Complex (PDC)**: It conversion of pyruvate to acetyl-CoA, linking glycolysis and the TCA cycle. Proximity prevents toxic accumulation of intermediates (e.g., acetaldehyde) and ensures synchronized catalytic activity. ***Proteins include:*** Pyruvate dehydrogenase (E1), Dihydrolipoamide acetyltransferase (E2), Dihydrolipoamide dehydrogenase (E3). **Reactants**: Pyruvate, Coenzyme A (CoA), NAD⁺; **Products:** Acetyl-CoA, NADH, CO₂

**7. Fatty acid synthase (FAS)**: A multi-functional enzyme complex containing acyl transferase, ketoacyl synthase, enoyl reductase, dehydratase, malonyl transferase, and thioesterase peptides assembled together, carries out the sequential reactions necessary to make a long-chain fatty acid like palmitate.

### **8.** **Spliceosome Complex:** Removes introns and splices exons together in pre-mRNA by sequential assembly, ensuring precise exon joining. **Proteins Involved:** snRNPs (U1, U2, U4/U6, U5) and splicing factors. **Reactants:** Pre-mRNA. **Products:** Mature mRNA.

**9. Non-Ribosomal Peptide Synthetases (NRPSs):** Biosynthesis of complex peptides such as antibiotics (e.g., vancomycin) and siderophores is brought about by covalently tethering substrates to carrier domains, processing them stepwise, and releasing the products upon completion. **Proteins Involved:** Modular enzymes containing domains like adenylation, thiolation, condensation, epimerization, and thioesterase. **Reactants:** Amino acids, ATP, cofactors. **Products:** Non-ribosomal peptides.

**10. Polyketide Synthases (PKSs):**  Biosynthesis of polyketides (e.g., erythromycin, tetracycline) is achieved by sequential assembly of carbon units, tethered to acyl carrier proteins. **Proteins Involved:** Modular enzymes containing acyltransferase, ketosynthase, ketoreductase, dehydratase, enoyl reductase, and thioesterase domains. **Reactants:** Malonyl-CoA, Acetyl-CoA, NADPH. **Products:** Polyketides.

## **11. Serine Protease**

**Function:** Cleaves peptide bonds in proteins using a **catalytic triad** (Ser-His-Asp).
**Examples:** Trypsin, Chymotrypsin, Elastase, Thrombin.

## **12. Amylase**

**Function:** Hydrolyzes starch into simpler sugars (maltose, glucose).
**Examples:** α-Amylase (salivary, pancreatic), β-Amylase (plants, bacteria).

## **13. Lipase**

**Function:** Hydrolyzes triglycerides into fatty acids and glycerol.
**Examples:** Pancreatic lipase, Hormone-sensitive lipase, Gastric lipase.

## **14. Ligase**

**Function:** Catalyzes the joining of two molecules using ATP or other energy sources.
**Examples:** DNA ligase, RNA ligase, Acetyl-CoA synthetase.

## **15. Aldolase**

**Function:** Catalyzes aldol condensation or cleavage of sugar molecules.
**Examples:** Aldolase A (glycolysis), Aldolase B (fructose metabolism).

**Structural details**

**Enzyme System Heme? Flavin? Fe-S Clusters?**

DNA Replication ❌ ❌ ❌

RNA Transcription ❌ ❌ ❌

Protein Translation ❌ ❌ ❌

DNA Repair ❌ ❌ ✅

Proteasome Machinery ❌ ❌ ❌

PDHC (Pyruvate Dehydrogenase Complex) ❌ ✅ ✅

FAS (Fatty Acid Synthase) ❌ (✅ in Bacteria) ❌

Spliceosome ❌ ❌ ❌

NRPS (Non-Ribosomal Peptide Synthesis) ❌ ✅ ✅

PKS (Polyketide Synthase) ✅ ✅ ✅

Serine Protease ❌ ❌ ❌

Amylase ❌ ❌ ❌

Lipase ❌ ❌ ❌

Ligase ❌ ❌ ❌

Aldolase ❌ ❌ ❌

**Data for murzymes**

| **Enzyme System** | **Heme?** | **Flavin?** | **FeS?** | **Constr. Access?** | **Subst > Site?** | **Redox?** | **Exergonic?** | **O₂ Need?** | **DRS?** | **Reversible?** | **Substrate Selectivity** | **Product Specificity** | **Modulator Diversity** | **Non-Integral Stoich?** | **Variable Stoich?** | **Unusual Kinetics (KM<Kd; KIE)** | **kcat > Diffusion?** | **Atypical Substrate Dep.?** | **Bulk Phase Dep.?** | **Temp Dep.?** |
| --- | --- | --- | --- | --- | --- | --- | --- | --- | --- | --- | --- | --- | --- | --- | --- | --- | --- | --- | --- | --- |
| **CPO** (Chloroperoxidase) | Yes | No | No | Partial | Yes | Yes | Yes | Yes/No | Yes | Partial | Low | Moderate | High | Yes | Yes | Yes | Yes | Yes | High | Atypical |
| **HRP** (Horseradish Peroxidase) | Yes | No | No | Partial | Yes | Yes | Yes | Yes/No | Yes | Partial | Low | Moderate | High | Yes | Yes | Yes | Yes | Yes | High | Atypical |
| **Cat** (Catalase) | Yes | No | No | Partial | Yes | Yes | Yes | Yes/No | Yes | Partial | Low | Moderate | Moderate | Yes | Yes | Yes | Yes | Yes | High | Atypical |
| **LiP** (Lignin Peroxidase) | Yes | No | No | Yes | Yes | Yes | Yes | Yes/No | Yes | Partial | Low | Moderate | High | Yes | Yes | Yes | Yes | Yes | High | Atypical |
| **LPO** (Lactoperoxidase) | Yes | No | No | Partial | Yes | Yes | Yes | Yes/No | Yes | Partial | Low | Moderate | High | Yes | Yes | Yes | Yes | Yes | High | Atypical |
| **COX1** (Cyclooxygenase-1) | Yes | No | No | Partial | Yes | Yes | Yes | Yes | Yes | Partial | Moderate | Moderate | High | Yes | Yes | Yes | Yes | Yes | High | Atypical |
| **CYP1A2** | Yes | No | No | Yes | Yes | Yes | Yes | Yes | Yes | Partial | Moderate | Moderate | High | Yes | Yes | Yes | Yes | Yes | High | Atypical |
| **CYP2B6** | Yes | No | No | Yes | Yes | Yes | Yes | Yes | Yes | Partial | Moderate | Moderate | High | Yes | Yes | Yes | Yes | Yes | High | Atypical |
| **CYP2C8** | Yes | No | No | Yes | Yes | Yes | Yes | Yes | Yes | Partial | Moderate | Moderate | High | Yes | Yes | Yes | Yes | Yes | High | Atypical |
| **Peroxisome** (as assembly) | Mixed | Mixed | No | Partial | Yes | Yes | Yes | Yes | Yes | Partial | Moderate | Moderate | High | Yes | Yes | Yes | Yes | Yes | High | Atypical |
| **MPO** (Myeloperoxidase) | Yes | No | No | Partial | Yes | Yes | Yes | Yes | Yes | Partial | Moderate | Moderate | High | Yes | Yes | Yes | Yes | Yes | High | Atypical |
| **CcP** (Cytochrome c Peroxidase) | Yes | No | No | Partial | Yes | Yes | Yes | Yes/No | Yes | Partial | Moderate | Moderate | High | Yes | Yes | Yes | Yes | Yes | High | Atypical |
| **APO** (Ascorbate peroxidase) | Yes | Sometimes | No | Partial | Yes | Yes | Yes | Yes/No | Yes | Partial | Moderate | Moderate | High | Yes | Yes | Yes | Yes | Yes | High | Atypical |
| **TPO** (Thyroid Peroxidase) | Yes | No | No | Partial | Yes | Yes | Yes | Yes/No | Yes | Partial | Moderate | Moderate | High | Yes | Yes | Yes | Yes | Yes | High | Atypical |
| **KatG** (Catalase-Peroxidase) | Yes | No | No | Partial | Yes | Yes | Yes | Yes/No | Yes | Partial | Moderate | Moderate | High | Yes | Yes | Yes | Yes | Yes | High | Atypical |
| **COX2** (Cyclooxygenase-2) | Yes | No | No | Partial | Yes | Yes | Yes | Yes | Yes | Partial | Moderate | Moderate | High | Yes | Yes | Yes | Yes | Yes | High | Atypical |
| **CYP2C19** | Yes | No | No | Yes | Yes | Yes | Yes | Yes | Yes | Partial | Moderate | Moderate | High | Yes | Yes | Yes | Yes | Yes | High | Atypical |
| **CYP2D6** | Yes | No | No | Yes | Yes | Yes | Yes | Yes | Yes | Partial | Moderate | Moderate | High | Yes | Yes | Yes | Yes | Yes | High | Atypical |
| **CYP2E1** | Yes | No | No | Yes | Yes | Yes | Yes | Yes | Yes | Partial | Moderate | Moderate | High | Yes | Yes | Yes | Yes | Yes | High | Atypical |
| **CYP3A4** | Yes | No | No | Yes | Yes | Yes | Yes | Yes | Yes | Partial | Moderate | Moderate | High | Yes | Yes | Yes | Yes | Yes | High | Atypical |
| **CYP2C9** | Yes | No | No | Yes | Yes | Yes | Yes | Yes | Yes | Partial | Moderate | Moderate | High | Yes | Yes | Yes | Yes | Yes | High | Atypical |
| **Mitochon** (Mitochondria assembly) | Yes | Yes | Yes | Partial | Yes | Yes | Yes | Yes | Yes | Partial | Moderate | Moderate | High | Yes | Yes | Yes | Yes | Yes | High | Atypical |

**Data for classical enzymes**

| **Enzyme System** | **Heme?** | **Flavin?** | **FeS?** | **Constr. Access?** | **Subst > Site?** | **Redox?** | **Exergonic?** | **O₂ Need?** | **DRS?** | **Reversible?** | **Substrate Selectivity** | **Product Specificity** | **Modulator Diversity** | **Non-Integral Stoich?** | **Variable Stoich?** | **Unusual Kinetics (KM<Kd; KIE)** | **kcat > Diffusion?** | **Atypical Substrate Dep.?** | **Bulk Phase Dep.?** | **Temp Dep.?** |
| --- | --- | --- | --- | --- | --- | --- | --- | --- | --- | --- | --- | --- | --- | --- | --- | --- | --- | --- | --- | --- |
| **DNA Replication** | No | No | No | Yes | Yes* | No | Yes | No | No | Yes | High | High | Low | No | No | No | No | No | Low | Typical |
| **RNA Transcription** | No | No | Yes | Yes | Yes* | Yes# | Yes | No | No | Yes | High | High | Medium | No | No | No | No | No | Low | Typical |
| **Protein Translation** | No | No | No | Yes | Yes* | No | Yes | No | No | Yes | High | High | Medium | No | No | No | No | No | Low | Typical |
| **DNA Repair** | No | No | Sometimes | Yes | Yes* | Sometimes | Yes | No | No | Sometimes | High | High | Medium | No | No | No | No | No | Low | Typical |
| **Proteasome Machinery** | No | No | No | Yes | Yes* | No | Yes | No | No | Yes | High | High | Medium | No | No | No | No | No | Low | Typical |
| **NRPS** (Non-ribosomal Peptide Synthetase) | No | No | No | Yes | Yes* | No | Yes | No | No | Yes | High | High | Medium | No | No | No | No | No | Low | Typical |
| **Amylase** | No | No | No | No | Yes* | No | Yes | No | No | Yes | High | High | Low | No | No | No | No | No | Low | Typical |
| **Lipase** | No | No | No | Yes | Yes* | No | Yes | No | No | Yes | High | High | Medium | No | No | No | No | No | Low | Typical |
| **Ligase** | No | No | No | Yes | Yes* | No | Yes | No | No | No | High | High | Low | No | No | No | No | No | Low | Typical |
| **Aldolase** | No | No | No | No | No | No | Yes | No | No | Yes | High | High | Low | No | No | No | No | No | Low | Typical |
| **PDHC** (Pyruvate Dehydrogenase Complex) | Yes | Yes | Yes | Yes | No | Yes | Yes | Yes | No | Partial | High | High | Medium | Yes | Yes | Sometimes | Yes | Yes | Medium | Atypical |
| **FAS** (Fatty Acid Synthase) | No | No | No | Yes | Yes* | No | Yes | No | No | Yes | High | High | Medium | No | No | No | No | No | Low | Typical |
| **Spliceosome** | No | No | No | Yes | Yes* | No | Yes | No | No | Yes | High | High | High | No | No | No | No | No | Low | Typical |
| **PKS** (Polyketide Synthase) | No | No | No | Yes | Yes* | No | Yes | No | No | Yes | High | High | Medium | No | No | No | No | No | Low | Typical |
| **Serine Protease** | No | No | No | Yes | Yes* | No | Yes | No | No | Yes | High | High | Low | No | No | No | Yes | No | Low | Typical |

**PART3: RECOGNITION OF MURZYMES FROM PDB FILES**

The third aspect of the work is delineating proteins into murzymes and non-murzymes from the pdb files grabbed from **the public repository of RCSB-PDB.** The pdb files are parsed to extract the fields of the pdb files most pertinent to the recognition of their potential to act in the functional roles of murzymes. This tool is envisaged as both assisitive to researchers in scraping potential murzymes from large repositories and a basis for further comprehensive analysis of proteins aimed at revealing the structural or other aspects that endow them with the functional capacity to act as murzymes. It is the future scope of the program is to include synthesis of murzymes which can carry out specific roles in a certain specialized biological context.

The flow of the recognition system can briefly be outlined as follows:

- Extraction of features from the pdb file (features are predoiminantly text based)
- Encoding of the text based features extracted using Large Language Models
- Classification the extracted features using various ML algorithms.

The detailed list of the pdb files used in training of the system is given below:

**MURZYMES**

**Respiratory proteins**

- **Cytochrome c oxidase (Complex IV)**:
  - PDB ID: 5B1A
  - Description: Cytochrome c oxidase is the terminal enzyme complex of the electron transport chain, catalyzing the reduction of oxygen to water.
- **Cytochrome c**:
  - PDB ID: 1HRC
  - Description: Cytochrome c is a small heme protein that shuttles electrons between Complex III and Complex IV in the mitochondrial respiratory chain.
- **Cytochrome b6f complex**:
  - PDB ID: 3S9V
  - Description: The cytochrome b6f complex mediates electron transfer between photosystem II and photosystem I in the electron transport chain of photosynthesis.
- **Cytochrome b5**:
  - PDB ID: 1CYO
  - Description: Cytochrome b5 is a heme-containing protein involved in various redox reactions, including electron transfer in fatty acid desaturation and drug metabolism.
- **Cytochrome c reductase (Complex III)**:
  - PDB ID: 1BGY
  - Description: Cytochrome c reductase, also known as Complex III, transfers electrons from ubiquinol to cytochrome c in the mitochondrial respiratory chain.
- **Cytochrome P450 reductase**:
  - PDB ID: 1AMO
  - Description: Cytochrome P450 reductase is a flavoprotein involved in electron transfer to cytochrome P450 enzymes, which play a role in drug metabolism and synthesis of steroid hormones.
- **NADH dehydrogenase (Complex I)**:
  - PDB ID: 7C3T
  - Description: NADH dehydrogenase, also known as Complex I, catalyzes the transfer of electrons from NADH to ubiquinone in the mitochondrial respiratory chain.
- **Cytochrome c peroxidase**:
  - PDB ID: 1EQY
  - Description: Cytochrome c peroxidase catalyzes the reduction of hydrogen peroxide using cytochrome c as an electron donor, protecting cells from oxidative damage.
- **Superoxide dismutase**:
  - PDB ID: 1VAR
  - Description: Superoxide dismutase catalyzes the conversion of superoxide radicals to oxygen and hydrogen peroxide, playing a role in antioxidant defense.
- **Ferredoxin**:
  - PDB ID: 3I6Q
  - Description: Ferredoxins are iron-sulfur proteins involved in electron transfer reactions in various metabolic pathways, including photosynthesis and nitrogen fixation.
- **Quinone oxidoreductase (NADPH2-dependent)**:
  - PDB ID: 1QRD
  - Description: Quinone oxidoreductase catalyzes the reduction of quinones using NADPH as a cofactor, playing a role in detoxification and redox regulation.
- **Flavodoxin**:
  - PDB ID: 1FSD
  - Description: Flavodoxins are flavin-containing proteins that function as electron carriers in various redox reactions, often replacing ferredoxins in anaerobic organisms.
- **Cytochrome P450**:
  - PDB ID: 3E6I
  - Description: Cytochrome P450 enzymes catalyze the oxidation of various organic substrates, including drugs, steroids, and fatty acids, in diverse metabolic pathways.
- **Cytochrome P450 2C9 (CYP2C9)**:
  - PDB ID: 1R9O
  - Description: Cytochrome P450 2C9 is a drug-metabolizing enzyme involved in the oxidation of various drugs and xenobiotics in the liver.
- **Cytochrome P450 3A4 (CYP3A4)**:
  - PDB ID: 1TQN
  - Description: Cytochrome P450 3A4 is the most abundant drug-metabolizing enzyme in the human liver, responsible for the metabolism of a wide range of drugs and xenobiotics.
- **Cytochrome P450 1A2 (CYP1A2)**:
  - PDB ID: 2HI4
  - Description: Cytochrome P450 1A2 is involved in the metabolism of endogenous compounds and xenobiotics, including caffeine, drugs, and environmental toxins.
- **Cytochrome P450 2D6 (CYP2D6)**:
  - PDB ID: 2F9Q
  - Description: Cytochrome P450 2D6 is a drug-metabolizing enzyme responsible for the metabolism of a wide range of drugs and xenobiotics in the liver.
- **Cytochrome P450 2E1 (CYP2E1)**:
  - PDB ID: 2F9Q
  - Description: Cytochrome P450 2E1 is involved in the metabolism of ethanol and various toxic compounds, playing a role in alcohol-induced liver damage and chemical carcinogenesis.
- **Cytochrome P450 2A6 (CYP2A6)**:
  - PDB ID: 5TLO
  - Description: Cytochrome P450 2A6 is involved in the metabolism of nicotine and various carcinogens found in tobacco smoke.
- **Cytochrome P450 2B6 (CYP2B6)**:
  - PDB ID: 4IQR
  - Description: Cytochrome P450 2B6 is involved in the metabolism of various drugs and environmental toxins, including anti-HIV medications and pesticides.
- **Cytochrome P450 2J2 (CYP2J2)**:
  - PDB ID: 3ZHE
  - Description: Cytochrome P450 2J2 is involved in the metabolism of arachidonic acid and other fatty acids, playing a role in cardiovascular homeostasis.
- **Cytochrome P450 2C19 (CYP2C19)**:
  - PDB ID: 2HP0
  - Description: Cytochrome P450 2C19 is involved in the metabolism of various drugs, including proton pump inhibitors and antidepressants.
- **Cytochrome P450 2D (CYP2D) family**:
  - PDB ID: 3QM8
  - Description: The Cytochrome P450 2D family includes enzymes involved in the metabolism of drugs and xenobiotics, with multiple isoforms.
- **Cytochrome P450 2F (CYP2F) family**:
  - PDB ID: 5ZP5
  - Description: The Cytochrome P450 2F family includes enzymes involved in the metabolism of various compounds, including drugs and environmental toxins.
- **Cytochrome P450 2S1 (CYP2S1)**:
  - PDB ID: 6CQ2
  - Description: Cytochrome P450 2S1 is involved in the metabolism of endogenous compounds and xenobiotics, with roles in carcinogenesis and drug metabolism.
- **Cytochrome P450 4F (CYP4F) family**:
  - PDB ID: 4UFG
  - Description: The Cytochrome P450 4F family includes enzymes involved in the metabolism of fatty acids and eicosanoids, with roles in inflammation and lipid homeostasis.
- **Cytochrome P450 27C1 (CYP27C1)**:
  - PDB ID: 5VHH
  - Description: Cytochrome P450 27C1 is involved in the metabolism of vitamin D3 and bile acids, playing a role in calcium homeostasis and cholesterol metabolism.
- **Cytochrome P450 11B2 (CYP11B2)**:
  - PDB ID: 1W0F
  - Description: Cytochrome P450 11B2 is involved in the biosynthesis of aldosterone, a hormone that regulates blood pressure and electrolyte balance.
- **Cytochrome P450 11A1 (CYP11A1)**:
  - PDB ID: 2NN2
  - Description: Cytochrome P450 11A1 is involved in the biosynthesis of steroid hormones, including cortisol and aldosterone.
- **Cytochrome P450 19A1 (CYP19A1)**:
  - PDB ID: 3S9D
  - Description: Cytochrome P450 19A1, also known as aromatase, is involved in the biosynthesis of estrogens from androgens.
- **Cytochrome P450 17A1 (CYP17A1)**:
  - PDB ID: 3RUK
  - Description: Cytochrome P450 17A1 is involved in the biosynthesis of steroid hormones, including androgens and glucocorticoids.
- **Cytochrome P450 27A1 (CYP27A1)**:
  - PDB ID: 5XMK
  - Description: Cytochrome P450 27A1 is involved in the metabolism of cholesterol and bile acids, playing a role in lipid homeostasis and bile acid synthesis.
- **Cytochrome P450 21A2 (CYP21A2)**:
  - PDB ID: 3QZV
  - Description: Cytochrome P450 21A2 is involved in the biosynthesis of aldosterone and cortisol, hormones that regulate electrolyte balance and stress response.
- **Cytochrome P450 51A1 (CYP51A1)**:
  - PDB ID: 1M0L
  - Description: Cytochrome P450 51A1 is involved in the biosynthesis of sterols, including cholesterol, in fungi and some protozoa.
- **Cytochrome P450 11A1 (CYP11A1)**:
  - PDB ID: 1SUO
  - Description: Cytochrome P450 11A1 is involved in the biosynthesis of steroid hormones, including cortisol and aldosterone.
- **Cytochrome P450 24A1 (CYP24A1)**:
  - PDB ID: 3K3R
  - Description: Cytochrome P450 24A1 is involved in the metabolism of vitamin D3 and other calcitriol derivatives, playing a role in calcium homeostasis and bone health.
- **Cytochrome P450 8B1 (CYP8B1)**:
  - PDB ID: 6QND
  - Description: Cytochrome P450 8B1 is involved in the biosynthesis of bile acids, regulating cholesterol metabolism and digestion.
- **Cytochrome P450 2W1 (CYP2W1)**:
  - PDB ID: 3ID6
  - Description: Cytochrome P450 2W1 is overexpressed in various cancers and is involved in the metabolism of xenobiotics and endogenous compounds.
- **Cytochrome P450 46A1 (CYP46A1)**:
  - PDB ID: 2HDI
  - Description: Cytochrome P450 46A1 is involved in the metabolism of cholesterol in the brain, playing a role in cholesterol homeostasis and neuroprotection.
- **Cytochrome P450 26A1 (CYP26A1)**:
  - PDB ID: 4R9N
  - Description: Cytochrome P450 26A1 is involved in the metabolism of retinoic acid, a signaling molecule important for embryonic development and cell differentiation.

**Xenobiotics and drug metabolizing proteins**

- **Cytochrome P450 3A4 (CYP3A4)**:
  - PDB ID: 1TQN
  - Description: Cytochrome P450 3A4 is the most abundant drug-metabolizing enzyme in the human liver, responsible for the metabolism of a wide range of drugs and xenobiotics.
- **Cytochrome P450 2D6 (CYP2D6)**:
  - PDB ID: 2F9Q
  - Description: Cytochrome P450 2D6 is involved in the metabolism of a wide range of drugs and xenobiotics, including antidepressants, antipsychotics, and opioids.
- **Cytochrome P450 2C9 (CYP2C9)**:
  - PDB ID: 1R9O
  - Description: Cytochrome P450 2C9 metabolizes various drugs, including nonsteroidal anti-inflammatory drugs (NSAIDs) and oral anticoagulants.
- **Cytochrome P450 1A2 (CYP1A2)**:
  - PDB ID: 2HI4
  - Description: Cytochrome P450 1A2 metabolizes caffeine, theophylline, and various environmental pollutants, including polycyclic aromatic hydrocarbons (PAHs).
- **Cytochrome P450 2E1 (CYP2E1)**:
  - PDB ID: 2F9Q
  - Description: Cytochrome P450 2E1 metabolizes ethanol, acetaminophen, and various industrial chemicals and environmental pollutants.
- **Sulfotransferase 1A1 (SULT1A1)**:
  - PDB ID: 1LS6
  - Description: SULT1A1 catalyzes the sulfation of drugs, hormones, and xenobiotics, increasing their water solubility for excretion.
- **Glutathione S-transferase P (GSTP1)**:
  - PDB ID: 1AQW
  - Description: GSTP1 catalyzes the conjugation of glutathione to electrophilic compounds, including drugs, toxins, and carcinogens, facilitating their elimination.
- **Cytochrome P450 2C19 (CYP2C19)**:
  - PDB ID: 2HP0
  - Description: Cytochrome P450 2C19 metabolizes various drugs, including proton pump inhibitors and antidepressants.
- **Cytochrome P450 2B6 (CYP2B6)**:
  - PDB ID: 4IQR
  - Description: Cytochrome P450 2B6 metabolizes various drugs and environmental toxins, including anti-HIV medications and pesticides.
  - including arylamines and heterocyclic amines.
- **Cytochrome P450 2J2 (CYP2J2)**:
  - PDB ID: 3ZHE
  - Description: Cytochrome P450 2J2 metabolizes arachidonic acid and epoxyeicosatrienoic acids, playing a role in cardiovascular homeostasis and inflammation.
  - exogenous compounds.
- **Cytochrome P450 2A6 (CYP2A6)**:
  - PDB ID: 5TLO
  - Description: Cytochrome P450 2A6 metabolizes nicotine and various carcinogens found in tobacco smoke.
- **Cytochrome P450 2W1 (CYP2W1)**:
  - PDB ID: 3ID6
  - Description: Cytochrome P450 2W1 is overexpressed in various cancers and metabolizes various drugs and xenobiotics.
- **Sulfotransferase 1A3 (SULT1A3)**:
  - PDB ID: 1LS6
  - Description: SULT1A3 catalyzes the sulfation of drugs and xenobiotics, contributing to their metabolism and elimination.
- **Cytochrome P450 2C8 (CYP2C8)**:
  - PDB ID: 1PQ2
  - Description: Cytochrome P450 2C8 metabolizes various drugs, including paclitaxel, rosiglitazone, and repaglinide.
- **Sulfotransferase 1A2 (SULT1A2)**:
  - PDB ID: 1LS6
  - Description: SULT1A2 catalyzes the sulfation of drugs, hormones, and xenobiotics, facilitating their metabolism and elimination.
- **Cytochrome P450 2F1 (CYP2F1)**:
  - PDB ID: 5ZP5
  - Description: Cytochrome P450 2F1 metabolizes various drugs and xenobiotics, including environmental pollutants and chemical carcinogens.
  - facilitating their excretion from the body.
- **Cytochrome P450 2B4 (CYP2B4)**:
  - PDB ID: 2HI4
  - Description: Cytochrome P450 2B4 metabolizes various drugs and xenobiotics, including benzene, toluene, and acetaminophen.
- **Cytochrome P450 2S1 (CYP2S1)**:
  - PDB ID: 6CQ2
  - Description: Cytochrome P450 2S1 is overexpressed in various cancers and metabolizes arachidonic acid and fatty acids.
- **Sulfotransferase 1B1 (SULT1B1)**:
  - PDB ID: 1LS6
  - Description: SULT1B1 catalyzes the sulfation of drugs and xenobiotics, contributing to their metabolism and elimination.
- **Monoamine oxidase A (MAO-A)**:
  - PDB ID: 2BXS
  - Description: MAO-A metabolizes neurotransmitters, such as serotonin, norepinephrine, and dopamine, as well as dietary amines and xenobiotics.
- **Cytochrome P450 4A11 (CYP4A11)**:
  - PDB ID: 4UFG
  - Description: Cytochrome P450 4A11 metabolizes fatty acids and eicosanoids, playing a role in inflammation and lipid homeostasis.
- **Sulfotransferase 1A4 (SULT1A4)**:
  - PDB ID: 1LS6
  - Description: SULT1A4 catalyzes the sulfation of drugs and xenobiotics, contributing to their metabolism and elimination.
- **Cytochrome P450 2J (CYP2J) family**:
  - PDB ID: 3QM8
  - Description: The Cytochrome P450 2J family metabolizes arachidonic acid and fatty acids, producing epoxyeicosatrienoic acids (EETs) with roles in cardiovascular homeostasis.
- **Cytochrome P450 2C18 (CYP2C18)**:
  - PDB ID: 1PQ2
  - Description: Cytochrome P450 2C18 metabolizes various drugs, including omeprazole and lansoprazole.
- **Sulfotransferase 1C4 (SULT1C4)**:
  - PDB ID: 1LS6
  - Description: SULT1C4 catalyzes the sulfation of drugs and xenobiotics, contributing to their metabolism and elimination.
- **NAD(P)H dehydrogenase quinone 1 (NQO1)**:
  - PDB ID: 4F3S
  - Description: NQO1 metabolizes quinones and other electrophilic compounds, protecting cells from oxidative stress and xenobiotic toxicity.
- **Cytochrome P450 2C (CYP2C) family**:
  - PDB ID: 3QM8
  - Description: The Cytochrome P450 2C family metabolizes various drugs, including nonsteroidal anti-inflammatory drugs (NSAIDs), oral anticoagulants, and antiepileptic drugs.
  - elimination.
- **Sulfotransferase 1C2 (SULT1C2)**:
  - PDB ID: 1LS6
  - Description: SULT1C2 catalyzes the sulfation of drugs and xenobiotics, contributing to their metabolism and elimination.
- **Cytochrome P450 2C8 (CYP2C8)**:
  - PDB ID: 1PQ2
  - Description: Cytochrome P450 2C8 metabolizes various drugs, including paclitaxel, rosiglitazone, and repaglinide.

**Photosynthetic proteins**

Sure, here are 50 photosynthetic proteins along with their corresponding PDB IDs:

- **Photosystem I (PSI)**:
  - PDB ID: 7C01
  - Description: Photosystem I is a large protein complex involved in the light-dependent reactions of photosynthesis, catalyzing the transfer of electrons from plastocyanin to ferredoxin.
- **Photosystem II (PSII)**:
  - PDB ID: 3WU2
  - Description: Photosystem II is a protein complex that catalyzes the light-induced oxidation of water, producing oxygen and protons while reducing plastoquinone to plastoquinol.
- **Cytochrome b6f complex**:
  - PDB ID: 7J9N
  - Description: The cytochrome b6f complex is involved in electron transfer between photosystem II and photosystem I in the thylakoid membrane of chloroplasts.
- **ATP synthase (CF1CF0 complex)**:
  - PDB ID: 5XNL
  - Description: ATP synthase is a complex enzyme that synthesizes ATP during the light-independent reactions of photosynthesis, using the proton gradient generated by the electron transport chain.
- **Ferredoxin-NADP+ reductase (FNR)**:
  - PDB ID: 1QAG
  - Description: FNR is an enzyme that catalyzes the transfer of electrons from ferredoxin to NADP+, producing NADPH, which is used in the Calvin cycle for carbon fixation.
- **Plastocyanin**:
  - PDB ID: 3PCY
  - Description: Plastocyanin is a small copper-containing protein involved in electron transfer between photosystem II and photosystem I in the thylakoid membrane.
- **Phycobilisome**:
  - PDB ID: 4F4M
  - Description: Phycobilisomes are large protein complexes found in cyanobacteria and red algae that harvest light energy and transfer it to the reaction centers of photosystems.
- **Light-harvesting complex II (LHCII)**:
  - PDB ID: 3PL9
  - Description: LHCII is a major antenna complex in photosystem II that absorbs light energy and transfers it to the reaction center chlorophylls.
- **Phytochrome**:
  - PDB ID: 4OUR
  - Description: Phytochromes are photoreceptor proteins that regulate various aspects of plant growth and development in response to red and far-red light signals.
- **Ferredoxin**:
  - PDB ID: 1GGX
  - Description: Ferredoxin is a small iron-sulfur protein that serves as an electron carrier in photosynthetic electron transport chains, transferring electrons to various acceptor proteins.
- **ATP synthase subunit alpha**:
  - PDB ID: 7BN4
  - Description: The alpha subunit of ATP synthase forms part of the catalytic core of the enzyme and participates in ATP synthesis.
- **ATP synthase subunit beta**:
  - PDB ID: 6KJ6
  - Description: The beta subunit of ATP synthase binds ADP and inorganic phosphate, facilitating the synthesis of ATP from ADP and Pi.
- **Cytochrome f**:
  - PDB ID: 4GCU
  - Description: Cytochrome f is a heme-containing protein that transfers electrons from the cytochrome b6f complex to plastocyanin in the thylakoid membrane.
- **PsbO (Oxygen-evolving enhancer protein)**:
  - PDB ID: 2AXT
  - Description: PsbO is an extrinsic protein associated with photosystem II that plays a role in stabilizing the oxygen-evolving complex and regulating water oxidation.
- **PsbP (23 kDa extrinsic protein)**:
  - PDB ID: 4XK8
  - Description: PsbP is an extrinsic protein associated with photosystem II that regulates the binding and release of calcium and chloride ions in the oxygen-evolving complex.
- **PsbQ (17 kDa extrinsic protein)**:
  - PDB ID: 3WU2
  - Description: PsbQ is an extrinsic protein associated with photosystem II that stabilizes the oxygen-evolving complex and facilitates the assembly of the Mn4CaO5 cluster.
- **Cytochrome c6**:
  - PDB ID: 4V90
  - Description: Cytochrome c6 is a heme-containing protein that transfers electrons from the cytochrome b6f complex to photosystem I in cyanobacteria and algae.
- **Ferredoxin-NADP+ reductase-like protein**:
  - PDB ID: 1R5H
  - Description: Ferredoxin-NADP+ reductase-like proteins are structurally related to FNR and may function in electron transfer pathways in various photosynthetic organisms.
- **Phycobiliproteins**:
  - PDB ID: 3UOD
  - Description: Phycobiliproteins are water-soluble accessory pigments found in cyanobacteria and red algae that absorb light energy and transfer it to chlorophylls.
- **Cytochrome c6A**:
  - PDB ID: 4ZAQ
  - Description: Cytochrome c6A is a heme-containing protein that transfers electrons from the cytochrome b6f complex to photosystem I in certain cyanobacteria.
- **Cytochrome c550**:
  - PDB ID: 2CJW
  - Description: Cytochrome c550 is a heme-containing protein involved in electron transfer reactions between photosystem II and the cytochrome b6f complex.
- **Photosystem I reaction center subunit IV**:
  - PDB ID: 3L3Q
  - Description: Photosystem I reaction center subunit IV is one of the core subunits of photosystem I, involved in the binding and stabilization of chlorophyll molecules.
- **Photosystem II reaction center protein D1**:
  - PDB ID: 3BZ0
  - Description: Photosystem II reaction center protein D1 is a core subunit of photosystem II that contains the catalytic site for water oxidation and binds the Mn4CaO5 cluster.
- **Photosystem II reaction center protein D2**:
  - PDB ID: 2AXT
  - Description: Photosystem II reaction center protein D2 is a core subunit of photosystem II that forms part of the reaction center complex involved in electron transfer.
- **Photosystem II CP47 protein**:
  - PDB ID: 1S5L
  - Description: Photosystem II CP47 protein is a core subunit of photosystem II that binds chlorophyll and helps stabilize the oxygen-evolving complex.
- **Photosystem II CP43 protein**:
  - PDB ID: 3WU2
  - Description: Photosystem II CP43 protein is a core subunit of photosystem II that binds chlorophyll and helps regulate the binding and release of water molecules during the oxygen-evolving reaction.
- **Photosystem II 10 kDa polypeptide**:
  - PDB ID: 2AXT
  - Description: The 10 kDa polypeptide of photosystem II is an extrinsic protein associated with the oxygen-evolving complex that stabilizes the Mn4CaO5 cluster.
- **Cytochrome c6A precursor**:
  - PDB ID: 3QRJ
  - Description: The precursor form of cytochrome c6A is synthesized and processed into the mature protein in cyanobacteria.
- **Cytochrome c-552 precursor**:
  - PDB ID: 2PJA
  - Description: The precursor form of cytochrome c-552 is synthesized and processed into the mature protein in photosynthetic bacteria.
- **Cytochrome f precursor**:
  - PDB ID: 3H1D
  - Description: The precursor form of cytochrome f is synthesized and processed into the mature protein in cyanobacteria and plants.
- **Cytochrome c553 precursor**:
  - PDB ID: 1AG6
  - Description: The precursor form of cytochrome c553 is synthesized and processed into the mature protein in photosynthetic bacteria.
- **PSII reaction center protein D2 precursor**:
  - PDB ID: 2AXT
  - Description: The precursor form of PSII reaction center protein D2 is synthesized and processed into the mature protein in cyanobacteria and plants.
- **Rubisco large subunit precursor**:
  - PDB ID: 9RUB
  - Description: The precursor form of Rubisco large subunit is synthesized and processed into the mature protein in photosynthetic organisms.
- **PsbO precursor**:
  - PDB ID: 2AXT
  - Description: The precursor form of PsbO is synthesized and processed into the mature protein in cyanobacteria and plants.
- **PsbP precursor**:
  - PDB ID: 2B10
  - Description: The precursor form of PsbP is synthesized and processed into the mature protein in cyanobacteria and plants.

**Light-harvesting complex proteins**

1. **Light-harvesting complex II (LHCII) from Spinach**:
   - PDB ID: 3PL9
   - Description: LHCII is a major light-harvesting complex found in the chloroplasts of plants and green algae, playing a crucial role in capturing light energy for photosynthesis.
2. **Light-harvesting complex I (LHCI) from Chlamydomonas reinhardtii**:
   - PDB ID: 6PP9
   - Description: LHCI is another type of light-harvesting complex found in the photosystems of plants and green algae, which absorbs light and transfers energy to the reaction center.
3. **Light-harvesting complex II (LH2) from Rhodopseudomonas acidophila**:
   - PDB ID: 1MQQ
   - Description: LH2 is a light-harvesting complex found in purple bacteria, responsible for capturing light energy and transferring it to the reaction center for photosynthesis.
4. **Light-harvesting complex (LH3) from Rhodopseudomonas acidophila**:
   - PDB ID: 2B3R
   - Description: LH3 is another light-harvesting complex found in purple bacteria, which complements LH2 in capturing light energy for photosynthesis.
5. **Light-harvesting complex II (LH2) from Marichromatium purpuratum**:
   - PDB ID: 2LH2
   - Description: LH2 from Marichromatium purpuratum is another example of a light-harvesting complex found in purple bacteria.
6. **Fucoxanthin-chlorophyll a/c protein (FCP) from Phaeodactylum tricornutum**:
   - PDB ID: 6IAU
   - Description: FCP is a light-harvesting complex found in diatoms, containing fucoxanthin and chlorophyll pigments for light absorption.
7. **Light-harvesting complex II (LHCII) from Arabidopsis thaliana**:
   - PDB ID: 1RWT
   - Description: LHCII from Arabidopsis thaliana is a plant light-harvesting complex that plays a key role in capturing and transferring light energy during photosynthesis.
8. **Photosystem II light-harvesting complex (LHCII) from Pisum sativum**:
   - PDB ID: 5LZO
   - Description: LHCII associated with Photosystem II from Pisum sativum is involved in light capture and energy transfer in the photosynthetic apparatus.
9. **Lutein-binding protein (LBP) from Arabidopsis thaliana**:
   - PDB ID: 3TH5
   - Description: LBP is a light-harvesting protein found in plants, which binds lutein molecules for light absorption and energy transfer.
10. **Light-harvesting complex I (LHI) from Thermosynechococcus elongatus**:
    - PDB ID: 5XNL
    - Description: LHI from Thermosynechococcus elongatus is a cyanobacterial light-harvesting complex involved in capturing light energy and transferring it to the photosynthetic reaction center.

**Non-specific flavoproteins**

- **Glucose oxidase (GOX)**:
  - - PDB ID: 1CF3
    - Description: Glucose oxidase catalyzes the oxidation of glucose to gluconic acid and hydrogen peroxide, using flavin adenine dinucleotide (FAD) as a cofactor.
- **Lactate oxidase**:
  - - PDB ID: 3W9N
    - Description: Lactate oxidase catalyzes the oxidation of lactate to pyruvate, producing hydrogen peroxide as a byproduct, and utilizes FAD as a cofactor.
- **Monoamine oxidase (MAO)**:
  - - PDB ID: 2BXS
    - Description: Monoamine oxidase catalyzes the oxidation of monoamine neurotransmitters, such as serotonin, norepinephrine, and dopamine, using flavin adenine dinucleotide (FAD) as a cofactor.
- **D-amino acid oxidase (DAO)**:
  - - PDB ID: 1DAA
    - Description: D-amino acid oxidase catalyzes the oxidation of D-amino acids to produce the corresponding alpha-keto acids and hydrogen peroxide, utilizing flavin adenine dinucleotide (FAD) as a cofactor.
- **Choline oxidase**:
  - - PDB ID: 1GPE
    - Description: Choline oxidase catalyzes the oxidation of choline to glycine betaine, producing hydrogen peroxide as a byproduct, and utilizes flavin adenine dinucleotide (FAD) as a cofactor.
- **Sarcosine oxidase**:
  - - PDB ID: 1B33
    - Description: Sarcosine oxidase catalyzes the oxidation of sarcosine to glycine, producing hydrogen peroxide as a byproduct, and utilizes flavin adenine dinucleotide (FAD) as a cofactor.
- **Pyranose oxidase**:
  - - PDB ID: 1PIH
    - Description: Pyranose oxidase catalyzes the oxidation of various carbohydrates, such as glucose and galactose, to the corresponding lactones, using flavin adenine dinucleotide (FAD) as a cofactor.
- **Dihydropyrimidine dehydrogenase (DPD)**:
  - - PDB ID: 1NU9
    - Description: Dihydropyrimidine dehydrogenase catalyzes the oxidation of dihydropyrimidines to the corresponding pyrimidines, utilizing flavin adenine dinucleotide (FAD) as a cofactor.
- **Vanillyl-alcohol oxidase (VAO)**:
  - - PDB ID: 1AMU
    - Description: Vanillyl-alcohol oxidase catalyzes the oxidation of vanillyl alcohol to vanillin, utilizing flavin adenine dinucleotide (FAD) as a cofactor.
- **D-amino acid oxidase (DAO)**:
  - - PDB ID: 4EXD
    - Description: D-amino acid oxidase catalyzes the oxidation of D-amino acids to produce the corresponding alpha-keto acids and hydrogen peroxide, utilizing nflavin adenine dinucleotide (FAD) as a cofactor.

**Non-specific iron-sulfur proteins:**

1. **Ferredoxin**:
   - PDB ID: 1W5C
   - Description: Ferredoxins are small iron-sulfur proteins that serve as electron carriers in various redox reactions, including photosynthesis and metabolism.
2. **High potential iron-sulfur protein (HiPIP)**:
   - PDB ID: 1HSA
   - Description: HiPIPs are small iron-sulfur proteins found in certain photosynthetic bacteria, where they participate in electron transfer reactions in the photosynthetic electron transport chain.
3. **Hydrogenase**:
   - PDB ID: 1HFE
   - Description: Hydrogenases are enzymes that catalyze the reversible conversion of molecular hydrogen (H2) to protons and electrons, utilizing iron-sulfur clusters as cofactors.
4. **NADH dehydrogenase (Complex I)**:
   - PDB ID: 6GCI
   - Description: NADH dehydrogenase, also known as Complex I of the electron transport chain, contains iron-sulfur clusters that facilitate electron transfer from NADH to ubiquinone.
5. **Succinate dehydrogenase (Complex II)**:
   - PDB ID: 1ZOY
   - Description: Succinate dehydrogenase is an enzyme complex involved in both the citric acid cycle and the electron transport chain, containing iron-sulfur clusters for electron transfer.
6. **Xanthine oxidase**:
   - PDB ID: 1FIQ
   - Description: Xanthine oxidase is an enzyme involved in purine metabolism, containing iron-sulfur clusters that participate in electron transfer reactions during the conversion of hypoxanthine to xanthine and xanthine to uric acid.
7. **Fumarate reductase**:
   - PDB ID: 1QLE
   - Description: Fumarate reductase is an enzyme involved in anaerobic respiration, containing iron-sulfur clusters that catalyze the reduction of fumarate to succinate.
8. **Isopropylmalate dehydrogenase**:
9. **Nitrate reductase**:
   - PDB ID: 1FYT
   - Description: Nitrate reductase is an enzyme involved in the reduction of nitrate to nitrite, containing iron-sulfur clusters that participate in electron transfer reactions.

**Peroxidases**

1. **Cytochrome c peroxidase**:
   - PDB ID: 1CPO
   - Description: Cytochrome c peroxidase is a heme-containing enzyme that catalyzes the reduction of hydrogen peroxide by cytochrome c, using a heme prosthetic group.
2. **Horseradish peroxidase**:
   - PDB ID: 1ATJ
   - Description: Horseradish peroxidase is a plant enzyme commonly used in molecular biology for detection and labeling assays, utilizing a heme prosthetic group.
3. **Manganese peroxidase**:
   - PDB ID: 2CZA
   - Description: Manganese peroxidase is an enzyme that catalyzes the oxidation of various organic compounds using hydrogen peroxide and manganese ions as cofactors.
4. **Lignin peroxidase**:
   - PDB ID: 1QML
   - Description: Lignin peroxidase is an enzyme involved in the degradation of lignin, catalyzing the oxidation of lignin-derived compounds using hydrogen peroxide and a heme prosthetic group.
5. **Thyroid peroxidase**:
   - PDB ID: 1YVB
   - Description: Thyroid peroxidase is an enzyme involved in the synthesis of thyroid hormones, catalyzing the iodination of tyrosine residues in thyroglobulin.
6. **Myeloperoxidase**:
   - PDB ID: 4N9X
   - Description: Myeloperoxidase is an enzyme found in neutrophil granules, where it catalyzes the production of hypochlorous acid and other reactive intermediates involved in microbial killing.
7. **Catalase**:
   - PDB ID: 1QQW
   - Description: Catalase is an enzyme that catalyzes the decomposition of hydrogen peroxide into water and oxygen, protecting cells from oxidative damage.
8. **Prostaglandin-H2 D-isomerase**:
   - PDB ID: 1DIY
   - Description: Prostaglandin-H2 D-isomerase, also known as prostaglandin-endoperoxide synthase, is an enzyme involved in prostaglandin biosynthesis, catalyzing the conversion of prostaglandin H2 to prostaglandin D2.
9. **Arabidopsis thaliana peroxidase 5**:
   - PDB ID: 1W84
   - Description: Arabidopsis thaliana peroxidase 5 is a peroxidase enzyme found in plants, involved in various physiological processes including defense against pathogens.
10. **Peroxiredoxin**:
    - PDB ID: 1QQW
    - Description: Peroxiredoxins are a family of antioxidant enzymes that catalyze the reduction of hydrogen peroxide, organic hydroperoxides, and peroxynitrite.
11. **Glutathione peroxidase**:
    - PDB ID: 1GP1
    - Description: Glutathione peroxidase is an antioxidant enzyme that catalyzes the reduction of hydrogen peroxide and organic hydroperoxides using glutathione as a cofactor.
12. **Chloroperoxidase**:
    - PDB ID: 2CPP
    - Description: Chloroperoxidase is a heme-containing enzyme found in fungi and bacteria, catalyzing the oxidation of halides and other substrates using hydrogen peroxide.
13. **Lactoperoxidase**:
    - PDB ID: 1LPG
    - Description: Lactoperoxidase is an enzyme found in milk and other secretions, where it catalyzes the oxidation of various substrates using hydrogen peroxide.
14. **Ascorbate peroxidase**:
    - PDB ID: 1APX
    - Description: Ascorbate peroxidase is an enzyme involved in the detoxification of hydrogen peroxide in plant cells, utilizing ascorbate as a reducing agent.
15. **Eosinophil peroxidase**:
    - PDB ID: 6H6C
    - Description: Eosinophil peroxidase is an enzyme found in eosinophil granules, where it catalyzes the production of hypobromous acid and other reactive intermediates involved in immune defense.
16. **Peroxiredoxin 1**:
    - PDB ID: 1QMV
    - Description: Peroxiredoxin 1 is an antioxidant enzyme that catalyzes the reduction of hydrogen peroxide and organic hydroperoxides, protecting cells from oxidative damage.
17. **Peroxiredoxin 2**:
    - PDB ID: 1QMV
    - Description: Peroxiredoxin 2 is another member of the peroxiredoxin family involved in antioxidant defense mechanisms.
18. **Peroxiredoxin 3**:
    - PDB ID: 1QMV
    - Description: Peroxiredoxin 3 is a mitochondrial peroxiredoxin involved in antioxidant defense and redox signaling.

19. **Peroxiredoxin 5**:

- - PDB ID: 1QMV
  - Description: Peroxiredoxin 5 is another member of the peroxiredoxin family localized in mitochondria and involved in antioxidant defense.

20. **Peroxiredoxin 6**:

- - PDB ID: 1QMV
  - Description: Peroxiredoxin 6 is a cytosolic enzyme that plays a role in protecting cells from oxidative stress.

**Hexaligated heme proteins**

Here are some PDB IDs of hexaligated heme proteins:

1. **Cytochrome c (Horse heart)**:
   - PDB ID: 1HRC
   - Description: Cytochrome c is a small heme-containing protein involved in electron transfer in the mitochondrial respiratory chain.
2. **Myoglobin (Equus caballus)**:
   - PDB ID: 1MBO
   - Description: Myoglobin is an oxygen-binding protein found in muscle tissues, facilitating oxygen storage and diffusion.
3. **Cytochrome c-551 (Pseudomonas aeruginosa)**:
   - PDB ID: 1DO7
   - Description: Cytochrome c-551 is a bacterial cytochrome involved in electron transfer reactions in the respiratory chain.
4. **Cytochrome b562 (Escherichia coli)**:
   - PDB ID: 1CYO
   - Description: Cytochrome b562 is a bacterial cytochrome involved in electron transfer reactions, containing a heme cofactor.
5. **Cytochrome b5 (Canis lupus familiaris)**:
   - PDB ID: 2CDJ
   - Description: Cytochrome b5 is a heme-containing protein involved in electron transfer reactions and lipid metabolism.
6. **Hemoglobin (Homo sapiens)**:
   - PDB ID: 2DN1
   - Description: Hemoglobin is a tetrameric protein found in red blood cells, responsible for oxygen transport from the lungs to tissues.
7. **Nitrite reductase (Escherichia coli)**:
   - PDB ID: 3NIR
   - Description: Nitrite reductase is an enzyme involved in the denitrification process, containing a heme cofactor.
8. **Catalase HPII (Escherichia coli)**:
   - PDB ID: 1QQW
   - Description: Catalase HPII is an enzyme that catalyzes the decomposition of hydrogen peroxide, utilizing a heme cofactor.
9. **Cytochrome c peroxidase (Saccharomyces cerevisiae)**:
   - PDB ID: 1CPO
   - Description: Cytochrome c peroxidase is an enzyme involved in hydrogen peroxide detoxification, containing a heme cofactor.
10. **Nitric oxide synthase (Bos taurus)**:
    - PDB ID: 1NOD
    - Description: Nitric oxide synthase is an enzyme that catalyzes the production of nitric oxide, containing a heme cofactor.

**Photoreceptive proteins**

1. **Rhodopsin**:
   - PDB ID: 1U19
   - Description: Rhodopsin is a light-sensitive receptor protein found in the retina of the eye, playing a crucial role in vision by initiating the phototransduction cascade.
2. **Bacteriorhodopsin**:
   - PDB ID: 1C3W
   - Description: Bacteriorhodopsin is a light-driven proton pump found in the plasma membrane of halophilic archaea, serving as an energy source for ATP synthesis.
3. **Channelrhodopsin-2 (ChR2)**:
   - PDB ID: 6EIK
   - Description: Channelrhodopsin-2 is a light-gated ion channel derived from green algae, used as a tool in optogenetics to control neuronal activity with light stimulation.
4. **Melanopsin**:
   - PDB ID: 4PHU
   - Description: Melanopsin is a photopigment found in retinal ganglion cells, involved in non-image-forming functions such as circadian rhythm regulation and pupillary light reflex.
5. **Cryptochrome-1 (CRY1)**:
   - PDB ID: 4JQI
   - Description: Cryptochrome-1 is a blue light photoreceptor involved in the regulation of circadian rhythms and light-dependent processes in plants and animals.
6. **Phototropin-1 (PHOT1)**:
   - PDB ID: 3OC4
   - Description: Phototropin-1 is a blue light photoreceptor involved in phototropism, chloroplast movement, and stomatal opening in plants.
7. **Ultraviolet (UV) photoreceptor UVR8**:
   - PDB ID: 4DNM
   - Description: UVR8 is a UV-B photoreceptor found in plants, regulating various UV-B-induced responses such as photomorphogenesis and stress acclimation.
8. **Photoactive yellow protein (PYP)**:
   - PDB ID: 1PYP
   - Description: PYP is a small blue-light photoreceptor found in certain bacteria, involved in phototaxis and photoregulation of gene expression.
9. **Neurospora circadian clock protein FREQUENCY (FRQ)**:
   - PDB ID: 4P6X
   - Description: FREQUENCY is a component of the Neurospora circadian clock, responding to light signals and regulating circadian rhythms.
10. **Opsin-3 (OPN3)**:
    - PDB ID: 6HUP
    - Description: Opsin-3 is a member of the opsin family of G-protein-coupled receptors, found in various tissues including the skin, eyes, and brain, and involved in light-sensing functions.

**Redox proteins with metal cofactors showing diverse substrate specificity**

1. **Cytochrome P450 1A2 (Human)**:
   - PDB ID: 2HI4
2. **Superoxide dismutase (SOD1, Human)**:
   - PDB ID: 3N07
3. **Cytochrome c oxidase (Bovine)**:
   - PDB ID: 5B1A
4. **Nitrate reductase (Arabidopsis thaliana)**:
   - PDB ID: 1JWA
5. **Copper-containing nitrite reductase (Pseudomonas aeruginosa)**:
   - PDB ID: 1Q1I
6. **Cytochrome c peroxidase (Saccharomyces cerevisiae)**:
   - PDB ID: 3CPP
7. **Manganese-dependent peroxidase (Phanerochaete chrysosporium)**:
   - PDB ID: 1DPU
8. **Alcohol dehydrogenase (Horse liver)**:
   - PDB ID: 4WCH
9. **Glutathione peroxidase (Human)**:
   - PDB ID: 1GP1
10. **Vanadium-dependent bromoperoxidase (Corallina officinalis)**:
    - PDB ID: 1BPO
11. **Iron-sulfur cluster assembly enzyme (Escherichia coli)**:
    - PDB ID: 4AM6
12. **Xanthine oxidase (Bovine)**:
    - PDB ID: 3HMX
13. **Cytochrome P450 3A4 (Human)**:
    - PDB ID: 2V0M
14. **Ribonucleotide reductase (Escherichia coli)**:
    - PDB ID: 1XIO
15. **Iron-regulated surface determinant protein A (Staphylococcus aureus)**:
    - PDB ID: 4NT8
16. **Chlorite dismutase (Pseudomonas chloritidismutans)**:
    - PDB ID: 2C2A
17. **Cytochrome c nitrite reductase (Desulfovibrio desulfuricans)**:
    - PDB ID: 1DYO
18. **Nickel-dependent hydrogenase (Desulfovibrio fructosovorans)**:
    - PDB ID: 1L0P
19. **Hydroxylamine oxidoreductase (Nitrosomonas europaea)**:
    - PDB ID: 1FM9
20. **Sulfite reductase (Escherichia coli)**:
    - PDB ID: 2VZ4
21. **Ferredoxin (Spinach)**:
    - PDB ID: 1F3N
22. **Cytochrome b5 (Human)**:
    - PDB ID: 1GAV
23. **NADH dehydrogenase (Escherichia coli)**:
    - PDB ID: 4HEA
24. **Catalase (Bacillus stearothermophilus)**:
    - PDB ID: 3NUV
25. **Hemoglobin (Human)**:
    - PDB ID: 1A3N
26. **Cytochrome P450cam (Pseudomonas putida)**:
    - PDB ID: 1J93
27. **Nitrogenase (Azotobacter vinelandii)**:
    - PDB ID: 3U7Q
28. **Sulfate reductase (Desulfovibrio vulgaris)**:
    - PDB ID: 2WVZ
29. **Rubredoxin (Desulfovibrio vulgaris)**:
    - PDB ID: 1URD
30. **Ferrochelatase (Saccharomyces cerevisiae)**:
    - PDB ID: 3R8H
31. **S-Adenosylmethionine synthetase (Escherichia coli)**:
    - PDB ID: 1AM7
32. **Cobalamin-dependent methionine synthase (Escherichia coli)**:
    - PDB ID: 1BKW
33. **Riboflavin synthase (Thermotoga maritima)**:
    - PDB ID: 3C5J
34. **NADH:ubiquinone oxidoreductase (Bos taurus)**:
    - PDB ID: 6IYT
35. **Methionine synthase (Human)**:
    - PDB ID: 2XFK
36. **Ferrochelatase (Saccharomyces cerevisiae)**:
    - PDB ID: 3R8H
37. **Rieske iron-sulfur protein (Thermus thermophilus)**:
    - PDB ID: 1RSP
38. **Cytochrome c (Horse heart)**:
    - PDB ID: 2YCC
39. **Cytochrome P450 2C9 (Human)**:
    - PDB ID: 1R9O
40. **Fumarate reductase (Escherichia coli)**:
    - PDB ID: 1KQG

**NON-MURZYMES**

**Isomerases**

1. **Glucose Isomerase**:
   - PDB ID: 1GIO
   - Description: Glucose isomerase catalyzes the reversible isomerization of glucose to fructose, a key step in the industrial production of high-fructose corn syrup and other sweeteners.
2. **Triosephosphate Isomerase**:
   - PDB ID: 1YPI
   - Description: Triosephosphate isomerase catalyzes the reversible isomerization of dihydroxyacetone phosphate (DHAP) to glyceraldehyde-3-phosphate (G3P) in the glycolytic pathway.
3. **Phosphoglucose Isomerase**:
   - PDB ID: 1PHI
   - Description: Phosphoglucose isomerase (also known as phosphoglucomutase) catalyzes the reversible isomerization of glucose-6-phosphate to glucose-1-phosphate, a key step in glycogen metabolism.
4. **Glucose-6-phosphate Isomerase**:
   - PDB ID: 2QFI
   - Description: Glucose-6-phosphate isomerase catalyzes the reversible isomerization of glucose-6-phosphate to fructose-6-phosphate, an important step in glycolysis and gluconeogenesis.
5. **Fumarate Hydratase (Fumarase)**:
   - PDB ID: 1QMF
   - Description: Fumarate hydratase catalyzes the reversible hydration of fumarate to malate in the tricarboxylic acid (TCA) cycle, also known as the citric acid cycle.
6. **Phosphogluconate Isomerase**:
   - PDB ID: 1PGI
   - Description: Phosphogluconate isomerase catalyzes the reversible isomerization of 6-phosphogluconate to 3-phosphoglycerate in the pentose phosphate pathway.
7. **Methylmalonyl-CoA Epimerase**:
   - PDB ID: 3LLQ
   - Description: Methylmalonyl-CoA epimerase catalyzes the reversible epimerization of (S)-methylmalonyl-CoA to (R)-methylmalonyl-CoA in the metabolism of propionyl-CoA.
8. **Mannose-6-phosphate Isomerase**:
   - PDB ID: 4DHB
   - Description: Mannose-6-phosphate isomerase catalyzes the reversible isomerization of mannose-6-phosphate to fructose-6-phosphate in carbohydrate metabolism.
9. **Cis-trans Isomerase**:
   - PDB ID: 1CJY
   - Description: Cis-trans isomerase (also known as peptidyl-prolyl cis-trans isomerase) catalyzes the cis-trans isomerization of peptide bonds, facilitating protein folding and conformational changes.
10. **Glutamine Synthetase**:

- PDB ID: 3VTA
- Description: Glutamine synthetase catalyzes the reversible amidation of glutamate to form glutamine, utilizing ammonia and ATP as substrates.

1. **Enolase**:

- PDB ID: 1ONE
- Description: Enolase catalyzes the reversible dehydration of 2-phospho-D-glycerate to phosphoenolpyruvate (PEP) in glycolysis.

1. **Mannose Isomerase**:

- PDB ID: 5F55
- Description: Mannose isomerase catalyzes the reversible isomerization of mannose to fructose, an important step in carbohydrate metabolism.

1. **Inositol-3-phosphate Synthase**:

- PDB ID: 3JUC
- Description: Inositol-3-phosphate synthase catalyzes the reversible conversion of glucose-6-phosphate to inositol-3-phosphate, a precursor in the biosynthesis of inositol phospholipids.

1. **Glutamate Racemase**:

- PDB ID: 2VCT
- Description: Glutamate racemase catalyzes the reversible racemization of L-glutamate to D-glutamate, a rare amino acid found in bacterial cell walls.

1. **Catalase**:

- PDB ID: 1DGF
- Description: Catalase catalyzes the decomposition of hydrogen peroxide (H2O2) into water and oxygen, protecting cells from oxidative damage.

1. **Proline Isomerase**:

- PDB ID: 1BR9
- Description: Proline isomerase (also known as cyclophilin) catalyzes the cis-trans isomerization of proline-containing peptide bonds, facilitating protein folding and assembly.

1. **Ribose-5-phosphate Isomerase**:

- PDB ID: 1R5P
- Description: Ribose-5-phosphate isomerase catalyzes the reversible isomerization of ribose-5-phosphate to ribulose-5-phosphate in the pentose phosphate pathway.

1. **Aconitate Hydratase (Aconitase)**:

- PDB ID: 1AIT
- Description: Aconitate hydratase catalyzes the reversible hydration of citrate to isocitrate in the tricarboxylic acid (TCA) cycle.

1. **Phosphoribosylanthranilate Isomerase**:

- PDB ID: 1RPN
- Description: Phosphoribosylanthranilate isomerase catalyzes the reversible isomerization of 1-(2-carboxyphenylamino)-1-deoxyribulose-5-phosphate to 1-(2-carboxyphenylamino)-1-deoxyribulose-5-phosphate in the tryptophan biosynthetic pathway.

Certainly! Here are 20 more examples of isomerases with their corresponding PDB IDs:

1. **Fucose-1-Phosphate Aldolase**:

- PDB ID: 2Q8I
- Description: Fucose-1-phosphate aldolase catalyzes the reversible cleavage of fucose-1-phosphate to produce glyceraldehyde-3-phosphate and dihydroxyacetone phosphate.

1. **Inositol 1,4,5-trisphosphate 3-kinase**:

- PDB ID: 1I01
- Description: Inositol 1,4,5-trisphosphate 3-kinase catalyzes the reversible phosphorylation of inositol 1,4,5-trisphosphate (IP3) to produce inositol 1,3,4,5-tetrakisphosphate (IP4).

1. **UDP-N-acetylglucosamine 2-epimerase**:

- PDB ID: 4RYF
- Description: UDP-N-acetylglucosamine 2-epimerase catalyzes the reversible epimerization of UDP-N-acetylglucosamine (UDP-GlcNAc) to UDP-N-acetylmannosamine (UDP-ManNAc).

1. **Alpha-L-Rhamnosidase**:

- PDB ID: 4MMT
- Description: Alpha-L-rhamnosidase catalyzes the reversible hydrolysis of alpha-L-rhamnosides to produce L-rhamnose and the corresponding alcohol.

1. **Adenylate Cyclase**:

- PDB ID: 1CJU
- Description: Adenylate cyclase catalyzes the reversible conversion of ATP to cyclic AMP (cAMP) and pyrophosphate.

1. **Xylose Isomerase**:

- PDB ID: 1XYI
- Description: Xylose isomerase catalyzes the reversible isomerization of D-xylose to D-xylulose, an important step in pentose sugar metabolism.

1. **Phosphoribosylaminoimidazolecarboxamide Formyltransferase**:

- PDB ID: 1CC1
- Description: Phosphoribosylaminoimidazolecarboxamide formyltransferase catalyzes the reversible formylation of phosphoribosylaminoimidazolecarboxamide (AICAR) to produce formyl-AICAR.

1. **Glutamate Racemase**:

- PDB ID: 2CWN
- Description: Glutamate racemase catalyzes the reversible racemization of L-glutamate to D-glutamate, an important process in bacterial cell wall biosynthesis.

1. **Phosphoribosylaminoimidazolecarboxamide Isomerase**:

- PDB ID: 3GFW
- Description: Phosphoribosylaminoimidazolecarboxamide isomerase catalyzes the reversible conversion of phosphoribosylaminoimidazolecarboxamide (AICAR) to phosphoribosylaminoimidazolesuccinocarboxamide (SAICAR).

1. **3-Deoxy-7-phosphoheptulonate Synthase**:

- PDB ID: 1KZ5
- Description: 3-Deoxy-7-phosphoheptulonate synthase catalyzes the reversible condensation of phosphoenolpyruvate (PEP) and D-erythrose-4-phosphate to form 3-deoxy-D-arabino-heptulosonate-7-phosphate (DAHP).

1. **Fructose-bisphosphate Aldolase**:

- PDB ID: 4ALD
- Description: Fructose-bisphosphate aldolase catalyzes the reversible cleavage of fructose 1,6-bisphosphate to glyceraldehyde 3-phosphate and dihydroxyacetone phosphate in glycolysis.

1. **Cis-aconitate Decarboxylase**:

- PDB ID: 5V3B
- Description: Cis-aconitate decarboxylase catalyzes the reversible decarboxylation of cis-aconitate to produce cis-itaconate, a key step in itaconate biosynthesis.

1. **4-Hydroxy-2-ketovalerate Aldolase**:

- PDB ID: 1OHK
- Description: 4-Hydroxy-2-ketovalerate aldolase catalyzes the reversible cleavage of 4-hydroxy-2-ketovalerate to pyruvate and acetaldehyde, an important step in the degradation of branched-chain amino acids.

1. **Phosphoribosylformylglycinamidine Synthase**:

- PDB ID: 1A34
- Description: Phosphoribosylformylglycinamidine synthase catalyzes the reversible conversion of formylglycinamide ribonucleotide (FGAR) to formylglycinamidine ribonucleotide (FGAM) in purine biosynthesis.

1. **Xylose Isomerase**:

- PDB ID: 3HQ1
- Description: Xylose isomerase catalyzes the reversible isomerization of D-xylose to D-xylulose, an important step in xylose metabolism.

**Lyases**

Sure! Here are 40 examples of lyases with their corresponding PDB IDs:

1. **Carbonic Anhydrase**:
   - PDB ID: 2CBA
   - Description: Carbonic anhydrase catalyzes the reversible hydration of carbon dioxide to bicarbonate ion and protons.
2. **Fumarate Lyase**:
   - PDB ID: 3PFL
   - Description: Fumarate lyase catalyzes the reversible cleavage of fumarate to produce acetyl-CoA and succinate.
3. **Citrate (pro-3S)-lyase**:
   - PDB ID: 1BW9
   - Description: Citrate (pro-3S)-lyase catalyzes the reversible cleavage of citrate to produce acetate and oxaloacetate.
4. **Adenylate Cyclase**:
   - PDB ID: 3NYN
   - Description: Adenylate cyclase catalyzes the conversion of ATP to cyclic AMP and pyrophosphate.
5. **Chalcone Synthase**:
   - PDB ID: 1CGK
   - Description: Chalcone synthase catalyzes the reversible condensation of three molecules of malonyl-CoA and one molecule of 4-coumaroyl-CoA to form naringenin chalcone.
6. **Farnesyl Diphosphate Synthase**:
   - PDB ID: 1RQ0
   - Description: Farnesyl diphosphate synthase catalyzes the formation of farnesyl diphosphate from isopentenyl diphosphate and dimethylallyl diphosphate.
7. **Oxalate CoA-transferase**:
   - PDB ID: 2NWO
   - Description: Oxalate CoA-transferase catalyzes the reversible transfer of CoA from succinyl-CoA to oxalate, producing succinate and oxalyl-CoA.
8. **Lanosterol Synthase**:
   - PDB ID: 3A68
   - Description: Lanosterol synthase catalyzes the cyclization of 2,3-oxidosqualene to form lanosterol, a precursor in cholesterol biosynthesis.
9. **2,3-diketo-L-gulonate 1,2-lyase**:

- PDB ID: 2OQJ
- Description: 2,3-diketo-L-gulonate 1,2-lyase catalyzes the reversible cleavage of 2,3-diketo-L-gulonate to form 5-dehydro-4-deoxy-D-gluconate.

1. **Homocitrate Synthase**:

- PDB ID: 4OBG
- Description: Homocitrate synthase catalyzes the condensation of acetyl-CoA and alpha-ketoglutarate to form homocitrate.

1. **Arginine Decarboxylase**:

- PDB ID: 2PYF
- Description: Arginine decarboxylase catalyzes the irreversible decarboxylation of arginine to agmatine.

1. **Farnesyl Diphosphate Synthase**:

- PDB ID: 1RQ2
- Description: Farnesyl diphosphate synthase catalyzes the formation of farnesyl diphosphate from isopentenyl diphosphate and dimethylallyl diphosphate.

1. **Homocitrate Synthase**:

- PDB ID: 4OBC
- Description: Homocitrate synthase catalyzes the condensation of acetyl-CoA and alpha-ketoglutarate to form homocitrate.

1. **Alkylglycerone-phosphate Synthase**:

- PDB ID: 2GBN
- Description: Alkylglycerone-phosphate synthase catalyzes the condensation of dihydroxyacetone phosphate and fatty acyl-CoA to form alkylglycerone phosphate.

1. **Adenosylmethionine Decarboxylase**:

- PDB ID: 1KTW
- Description: Adenosylmethionine decarboxylase catalyzes the irreversible decarboxylation of S-adenosylmethionine (SAM) to form S-adenosylmethioninamine (dcAdoMet).

1. **L-ribulose-5-phosphate 4-epimerase**:

- PDB ID: 1U1X
- Description: L-ribulose-5-phosphate 4-epimerase catalyzes the reversible epimerization of L-ribulose-5-phosphate to D-xylulose-5-phosphate.

1. **ATP-citrate Synthase**:

- PDB ID: 2ZK9
- Description: ATP-citrate synthase catalyzes the condensation of acetyl-CoA and oxaloacetate to form citrate, utilizing ATP as a cofactor.

1. **Methionine-gamma-lyase**:

- PDB ID: 3NFY
- Description: Methionine-gamma-lyase catalyzes the cleavage of L-methionine to produce methanethiol, alpha-ketobutyrate, and ammonia.

1. **UDP-galactose 4-epimerase**:

- PDB ID: 1NPH
- Description: UDP-galactose 4-epimerase catalyzes the reversible epimerization of UDP-galactose to UDP-glucose.

1. **Thymidylate Synthase**:

- PDB ID: 2DTM
- Description: Thymidylate synthase catalyzes the conversion of deoxyuridine monophosphate (dUMP) to deoxythymidine monophosphate (dTMP) by methylating dUMP using 5,10-methylenetetrahydrofolate (mTHF) as a cofactor.

1. **Glutamate Synthase**:

- PDB ID: 3GLS
- Description: Glutamate synthase catalyzes the reversible conversion of glutamine and 2-oxoglutarate to glutamate and glutamate semialdehyde.

1. **UDP-glucose 4-epimerase**:

- PDB ID: 1RJ7
- Description: UDP-glucose 4-epimerase catalyzes the reversible epimerization of UDP-glucose to UDP-galactose.

1. **Hydroxymethylbilane Synthase**:

- PDB ID: 4J8A
- Description: Hydroxymethylbilane synthase catalyzes the reversible condensation of four molecules of porphobilinogen to form hydroxymethylbilane, a precursor in heme biosynthesis.

1. **6-Phospho-beta-galactosidase**:

- PDB ID: 2BT5
- Description: 6-Phospho-beta-galactosidase catalyzes the reversible hydrolysis of 6-phospho-beta-galactosides to produce D-galactose 6-phosphate and orthophosphate.

1. **Pyridoxal-5'-phosphate synthase**:

- PDB ID: 1Z57
- Description: Pyridoxal-5'-phosphate synthase catalyzes the reversible condensation of ribose 5-phosphate and glyceraldehyde 3-phosphate to form pyridoxal 5'-phosphate, a cofactor in various enzymatic reactions.

1. **Fumarate Hydratase**:

- PDB ID: 1KE2
- Description: Fumarate hydratase catalyzes the reversible hydration of fumarate to form L-malate.

1. **Aspartate Ammonia-lyase**:

- PDB ID: 4BBH
- Description: Aspartate ammonia-lyase catalyzes the reversible deamination of L-aspartate to form fumarate and ammonia.

1. **Cystathionine beta-lyase**:

- PDB ID: 1KB2
- Description: Cystathionine beta-lyase catalyzes the cleavage of cystathionine to produce homocysteine and cysteine.

1. **Hydroxyethylthiazole Kinase**:

- PDB ID: 2A1Q
- Description: Hydroxyethylthiazole kinase catalyzes the reversible phosphorylation of hydroxyethylthiazole to form hydroxyethylthiazole phosphate, an important step in thiamine biosynthesis.

1. **Glutathione-dependent formaldehyde-activating enzyme**:

- PDB ID: 4O7F
- Description: Glutathione-dependent formaldehyde-activating enzyme catalyzes the reversible condensation of formaldehyde with glutathione to form S-hydroxymethylglutathione.

1. **Chloramphenicol Acetyltransferase**:

- PDB ID: 1XDN
- Description: Chloramphenicol acetyltransferase catalyzes the reversible acetylation of chloramphenicol, conferring resistance to this antibiotic in bacteria.

**Carbohydrases**

1. **Alpha-Amylase**:
   - PDB ID: 1SMD
   - Description: Alpha-amylase catalyzes the hydrolysis of alpha-1,4-glycosidic linkages in starch and glycogen, producing shorter oligosaccharides.
2. **Beta-Galactosidase**:
   - PDB ID: 1DP0
   - Description: Beta-galactosidase catalyzes the hydrolysis of beta-1,4-glycosidic linkages in lactose, releasing galactose and glucose.
3. **Beta-Glucosidase**:
   - PDB ID: 1EXG
   - Description: Beta-glucosidase catalyzes the hydrolysis of beta-1,4-glycosidic linkages in cellulose and other beta-glucans, releasing glucose.
4. **Cellulase**:
   - PDB ID: 1CEN
   - Description: Cellulase is a complex of enzymes that hydrolyze cellulose into smaller oligosaccharides and glucose units.
5. **Chitinase**:
   - PDB ID: 1CTN
   - Description: Chitinase catalyzes the hydrolysis of beta-1,4-glycosidic linkages in chitin, a structural polysaccharide found in the exoskeletons of arthropods and fungi.
6. **Lactase**:
   - PDB ID: 1LAC
   - Description: Lactase catalyzes the hydrolysis of lactose into galactose and glucose, facilitating lactose digestion in mammals.
7. **Amyloglucosidase**:
   - PDB ID: 1AGS
   - Description: Amyloglucosidase (glucoamylase) catalyzes the hydrolysis of alpha-1,4-glycosidic linkages in starch and glycogen, releasing glucose.
8. **Alpha-Glucosidase**:
   - PDB ID: 1A4G
   - Description: Alpha-glucosidase catalyzes the hydrolysis of alpha-1,4-glycosidic linkages in oligosaccharides and maltose, releasing glucose.
9. **Maltase**:
   - PDB ID: 1MLT
   - Description: Maltase catalyzes the hydrolysis of maltose into two molecules of glucose.
10. **Sucrase**:

- PDB ID: 4E8K
- Description: Sucrase catalyzes the hydrolysis of sucrose into glucose and fructose.

1. **Alpha-Galactosidase**:

- PDB ID: 2HHS
- Description: Alpha-galactosidase catalyzes the hydrolysis of alpha-1,6-galactosidic linkages in complex oligosaccharides, releasing galactose.

1. **Cellulase**:

- PDB ID: 1GZT
- Description: Cellulase is a complex of enzymes that hydrolyze cellulose into smaller oligosaccharides and glucose units.

1. **Glycoside Hydrolase**:

- PDB ID: 3TOP
- Description: Glycoside hydrolase catalyzes the hydrolysis of glycosidic bonds in various glycosides, releasing monosaccharides or oligosaccharides.

1. **Alpha-L-Arabinofuranosidase**:

- PDB ID: 2VUE
- Description: Alpha-L-arabinofuranosidase catalyzes the hydrolysis of alpha-1,2,3, or 5-arabinofuranosidic linkages in arabinoxylans, releasing arabinose.

1. **Xylanase**:

- PDB ID: 4W6J
- Description: Xylanase catalyzes the hydrolysis of beta-1,4-glycosidic linkages in xylan, releasing xylose.

1. **Beta-Xylosidase**:

- PDB ID: 1XYN
- Description: Beta-xylosidase catalyzes the hydrolysis of beta-1,4-glycosidic linkages in xylooligosaccharides, releasing xylose.

1. **Glycogen Phosphorylase**:

- PDB ID: 1GPB
- Description: Glycogen phosphorylase catalyzes the phosphorolytic cleavage of glycogen to release glucose-1-phosphate.

1. **Alpha-1,6-Glucosidase**:

- PDB ID: 1GZE
- Description: Alpha-1,6-glucosidase catalyzes the hydrolysis of alpha-1,6-glycosidic linkages in glycogen and amylopectin.

1. **Glucan 1,4-alpha-Maltohydrolase**:

- PDB ID: 3NRY
- Description: Glucan 1,4-alpha-maltohydrolase catalyzes the hydrolysis of alpha-1,4-glycosidic linkages in maltodextrins and glycogen.

1. **Levanase**:

- PDB ID: 3NV5
- Description: Levanase catalyzes the hydrolysis of beta-2,6-fructosidic linkages in levan, releasing fructose.

1. **Inulinase**:

- PDB ID: 1ZD2
- Description: Inulinase catalyzes the hydrolysis of beta-2,1-fructosidic linkages in inulin, releasing fructose.

1. **Glycosyltransferase**:

- PDB ID: 2O3Y
- Description: Glycosyltransferase catalyzes the transfer of a sugar moiety from a donor molecule to an acceptor molecule, forming glycosidic bonds.

1. **Mannanase**:

- PDB ID: 2EU3
- Description: Mannanase catalyzes the hydrolysis of beta-1,4-glycosidic linkages in mannan, releasing mannose.

1. **Pectinesterase**:

- PDB ID: 2J5C
- Description: Pectinesterase catalyzes the de-esterification of pectin, releasing methanol and producing pectate.

1. **Streptavidin**:

- PDB ID: 1MK5
- Description: Streptavidin is a protein that binds biotin with high affinity, commonly used in biochemical assays and biotechnology applications.

1. **Chitin Deacetylase**:

- PDB ID: 1ESY
- Description: Chitin deacetylase catalyzes the deacetylation of chitin, converting N-acetylglucosamine residues to glucosamine residues.

1. **Xyloglucanase**:

- PDB ID: 1QF9
- Description: Xyloglucanase catalyzes the hydrolysis of xyloglucans, releasing xylose and other oligosaccharides.

1. **Galactanase**:

- PDB ID: 2CMY
- Description: Galactanase catalyzes the hydrolysis of beta-1,4-glycosidic linkages in galactans, releasing galactose.

1. **Arabinanase**:

- PDB ID: 3W9O
- Description: Arabinanase catalyzes the hydrolysis of alpha-1,5-arabinofuranosidic linkages in arabinans, releasing arabinose.

1. **Alpha-Mannosidase**:

- PDB ID: 1OBB
- Description: Alpha-mannosidase catalyzes the hydrolysis of alpha-1,2,3,6-mannosidic linkages in oligosaccharides, releasing mannose.

1. **Glycogen Phosphorylase**:

- PDB ID: 1GPB
- Description: Glycogen phosphorylase catalyzes the phosphorolytic cleavage of glycogen to release glucose-1-phosphate.

1. **Alpha-1,6-Glucosidase**:

- PDB ID: 1GZE
- Description: Alpha-1,6-glucosidase catalyzes the hydrolysis of alpha-1,6-glycosidic linkages in glycogen and amylopectin.

1. **Glucan 1,4-alpha-Maltohydrolase**:

- PDB ID: 3NRY
- Description: Glucan 1,4-alpha-maltohydrolase catalyzes the hydrolysis of alpha-1,4-glycosidic linkages in maltodextrins and glycogen.

1. **Levanase**:

- PDB ID: 3NV5
- Description: Levanase catalyzes the hydrolysis of beta-2,6-fructosidic linkages in levan, releasing fructose.

1. **Inulinase**:

- PDB ID: 1ZD2
- Description: Inulinase catalyzes the hydrolysis of beta-2,1-fructosidic linkages in inulin, releasing fructose.

1. **Glycosyltransferase**:

- PDB ID: 2O3Y
- Description: Glycosyltransferase catalyzes the transfer of a sugar moiety from a donor molecule to an acceptor molecule, forming glycosidic bonds.

1. **Mannanase**:

- PDB ID: 2EU3
- Description: Mannanase catalyzes the hydrolysis of beta-1,4-glycosidic linkages in mannan, releasing mannose.

1. **Chitin Deacetylase**:

- PDB ID: 1ESY
- Description: Chitin deacetylase catalyzes the deacetylation of chitin, converting N-acetylglucosamine residues to glucosamine residues.

**Lipases**

Sure! Here are 40 examples of lipases with their corresponding PDB IDs:

1. **Pancreatic Lipase**:
   - PDB ID: 1LPB
   - Description: Pancreatic lipase catalyzes the hydrolysis of triglycerides into fatty acids and glycerol.
2. **Lipase A**:
   - PDB ID: 1TGL
   - Description: Lipase A from Candida antarctica is an enzyme used in various biotechnological applications.
3. **Lipase B**:
   - PDB ID: 1LBS
   - Description: Lipase B from Candida antarctica is another isoform of the lipase enzyme.
4. **Lipase C**:
   - PDB ID: 1I6V
   - Description: Lipase C from Candida rugosa is used in various industrial processes.
5. **Human Gastric Lipase**:
   - PDB ID: 1LPA
   - Description: Human gastric lipase is an enzyme involved in the digestion of dietary fats.
6. **Human Hepatic Lipase**:
   - PDB ID: 2TRH
   - Description: Human hepatic lipase is an enzyme involved in lipid metabolism and high-density lipoprotein (HDL) metabolism.
7. **Phospholipase A2**:
   - PDB ID: 1POC
   - Description: Phospholipase A2 catalyzes the hydrolysis of phospholipids at the sn-2 position, releasing fatty acids and lysophospholipids.
8. **Lipoprotein Lipase**:
   - PDB ID: 1D0Y
   - Description: Lipoprotein lipase is an enzyme that hydrolyzes triglycerides in lipoproteins, releasing fatty acids for cellular uptake.
9. **Triacylglycerol Lipase**:
   - PDB ID: 1UJ2
   - Description: Triacylglycerol lipase catalyzes the hydrolysis of triacylglycerols into fatty acids and glycerol.
10. **Lecithin-Cholesterol Acyltransferase**:

- PDB ID: 1LCB
- Description: Lecithin-cholesterol acyltransferase catalyzes the transfer of fatty acids from phosphatidylcholine to cholesterol, forming cholesterol esters.

1. **Human Lysosomal Acid Lipase**:

- PDB ID: 4GXZ
- Description: Human lysosomal acid lipase is an enzyme involved in the breakdown of cholesterol esters and triglycerides within lysosomes.

1. **Human Endothelial Lipase**:

- PDB ID: 2N7C
- Description: Human endothelial lipase is an enzyme involved in lipid metabolism and lipoprotein metabolism.

1. **GDSL Lipase**:

- PDB ID: 3QGG
- Description: GDSL lipase is a versatile enzyme with various substrate specificities and biotechnological applications.

1. **Candida antarctica Lipase B Mutant**:

- PDB ID: 3CXE
- Description: Mutant form of Candida antarctica lipase B with altered substrate specificity.

1. **Acylglycerol Kinase**:

- PDB ID: 5NFG
- Description: Acylglycerol kinase is an enzyme involved in the phosphorylation of monoacylglycerols and diacylglycerols.

1. **Cytosolic Phospholipase A2**:

- PDB ID: 1CJY
- Description: Cytosolic phospholipase A2 catalyzes the release of arachidonic acid from membrane phospholipids.

1. **GDSL Lipase**:

- PDB ID: 3QGG
- Description: GDSL lipase is a versatile enzyme with various substrate specificities and biotechnological applications.

1. **Escherichia coli Lipase**:

- PDB ID: 1LPY
- Description: Lipase from Escherichia coli, involved in lipid metabolism and bacterial survival.

1. **Pseudomonas aeruginosa Lipase**:

- PDB ID: 1DTW
- Description: Lipase from Pseudomonas aeruginosa, involved in bacterial lipid metabolism and virulence.

1. **Rhizopus niveus Lipase**:

- PDB ID: 1RNL
- Description: Lipase from Rhizopus niveus, a fungus used in various biotechnological applications.

1. **Thermomyces lanuginosus Lipase**:

- PDB ID: 1TGN
- Description: Lipase from Thermomyces lanuginosus, commonly used in industrial processes.

1. **Candida antarctica Lipase A Mutant**:

- PDB ID: 1GQP
- Description: Mutant form of Candida antarctica lipase A with altered substrate specificity.

1. **Mucor miehei Lipase**:

- PDB ID: 1UO4
- Description: Lipase from Mucor miehei, commonly used in food and pharmaceutical industries.

1. **Penicillium camembertii Lipase**:

- PDB ID: 1BKH
- Description: Lipase from Penicillium camembertii, involved in cheese ripening processes.

1. **Rhizopus oryzae Lipase**:

- PDB ID: 1ROL
- Description: Lipase from Rhizopus oryzae, used in various industrial applications.

1. **Geotrichum candidum Lipase**:

- PDB ID: 1JN2
- Description: Lipase from Geotrichum candidum, involved in cheese ripening processes.

1. **Aspergillus niger Lipase**:

- PDB ID: 1HYP
- Description: Lipase from Aspergillus niger, used in various biotechnological applications.

1. **Burkholderia cepacia Lipase**:

- PDB ID: 1BD6
- Description: Lipase from Burkholderia cepacia, involved in lipid metabolism and bioremediation.

1. **Candida rugosa Lipase**:

- PDB ID: 1TCA
- Description: Lipase from Candida rugosa, widely used in industrial processes.

1. **Rhizopus niveus Lipase**:

- PDB ID: 1LBA
- Description: Lipase from Rhizopus niveus, a fungus used in various biotechnological applications.

1. **Staphylococcus aureus Lipase**:

- PDB ID: 1FX8
- Description: Lipase from Staphylococcus aureus, involved in bacterial lipid metabolism and virulence.

1. **Yarrowia lipolytica Lipase**:

- PDB ID: 1LPK
- Description: Lipase from Yarrowia lipolytica, used in various industrial processes.

1. **Candida antarctica Lipase B Mutant**:

- PDB ID: 3CXE
- Description: Mutant form of Candida antarctica lipase B with altered substrate specificity.

1. **Streptomyces exfoliatus Lipase**:

- PDB ID: 1LEW
- Description: Lipase from Streptomyces exfoliatus, involved in lipid metabolism and biotechnological applications.

1. **Aspergillus niger Lipase**:

- PDB ID: 1KMU
- Description: Lipase from Aspergillus niger, used in various biotechnological applications.

1. **Geotrichum candidum Lipase**:

- PDB ID: 1JN2
- Description: Lipase from Geotrichum candidum, involved in cheese ripening processes.

1. **Mucor javanicus Lipase**:

- PDB ID: 1GJ7
- Description: Lipase from Mucor javanicus, used in various industrial processes.

1. **Pseudomonas fluorescens Lipase**:

- PDB ID: 1TGN
- Description: Lipase from Pseudomonas fluorescens, involved in lipid metabolism and bioremediation.

1. **Candida antarctica Lipase A Mutant**:

- PDB ID: 1GQP
- Description: Mutant form of Candida antarctica lipase A with altered substrate specificity.

1. **Rhizopus niveus Lipase**:

- PDB ID: 1LBA
- Description: Lipase from Rhizopus niveus, a fungus used in various biotechnological applications.

**Non-metallic proteases**

Certainly! Here are 40 examples of non-metalloproteases with their corresponding PDB IDs:

1. **Papain**:
   - PDB ID: 1PPN
   - Description: Papain is a cysteine protease found in papaya latex, commonly used in biochemical research.
2. **Cathepsin B**:
   - PDB ID: 1GMY
   - Description: Cathepsin B is a lysosomal cysteine protease involved in various cellular processes, including protein degradation.
3. **Chymotrypsin**:
   - PDB ID: 2CHA
   - Description: Chymotrypsin is a serine protease that cleaves peptide bonds at the carboxyl side of aromatic amino acids.
4. **Trypsin**:
   - PDB ID: 1SPH
   - Description: Trypsin is a serine protease that cleaves peptide bonds at the carboxyl side of lysine and arginine residues.
5. **Thrombin**:
   - PDB ID: 1HCG
   - Description: Thrombin is a serine protease involved in blood clotting, converting fibrinogen into fibrin.
6. **Subtilisin**:
   - PDB ID: 1SBT
   - Description: Subtilisin is a serine protease produced by bacteria, commonly used in industrial applications.
7. **Pepsin A**:
   - PDB ID: 1PSO
   - Description: Pepsin A is an aspartic protease found in the stomach, involved in the digestion of dietary proteins.
8. **Renin**:
   - PDB ID: 1ROP
   - Description: Renin is an aspartic protease involved in the regulation of blood pressure through the renin-angiotensin system.
9. **Caspase-3**:
   - PDB ID: 1CPP
   - Description: Caspase-3 is a cysteine protease involved in apoptosis, cleaving various cellular substrates during programmed cell death.
10. **Caspase-7**:
    - PDB ID: 1F1J
    - Description: Caspase-7 is another cysteine protease involved in apoptosis, with similar functions to caspase-3.
11. **Caspase-8**:
    - PDB ID: 2YJ5
    - Description: Caspase-8 is a cysteine protease involved in apoptosis and immune signaling pathways.
12. **Granzyme B**:
    - PDB ID: 1GRZ
    - Description: Granzyme B is a serine protease released by cytotoxic T cells and natural killer cells to induce apoptosis in target cells.
13. **Legumain**:
    - PDB ID: 2E3A
    - Description: Legumain is an asparagine endopeptidase involved in antigen processing and lysosomal protein degradation.
14. **Calpain-1**:
    - PDB ID: 1KFU
    - Description: Calpain-1 is a calcium-dependent cysteine protease involved in various cellular processes, including cytoskeletal remodeling.
15. **Calpain-2**:
    - PDB ID: 1KFX
    - Description: Calpain-2 is another isoform of calcium-dependent cysteine protease, with similar functions to calpain-1.
16. **Cysteine Protease ATG4B**:
    - PDB ID: 3O0T
    - Description: ATG4B is a cysteine protease involved in autophagy, cleaving the C-terminal residues of microtubule-associated protein 1A/1B-light chain 3 (LC3).
17. **Glutamate Carboxypeptidase II**:
    - PDB ID: 4N5F
    - Description: Glutamate carboxypeptidase II, also known as prostate-specific membrane antigen (PSMA), is a zinc metalloprotease involved in glutamate metabolism.
18. **Gingipain R**:
    - PDB ID: 1JSI
    - Description: Gingipain R is a cysteine protease produced by the periodontal pathogen Porphyromonas gingivalis, involved in tissue degradation and evasion of host immune responses.
19. **Gingipain K**:
    - PDB ID: 2B5X
    - Description: Gingipain K is another cysteine protease produced by Porphyromonas gingivalis, with similar functions to gingipain R.
20. **Granzyme A**:
    - PDB ID: 2VAD
    - Description: Granzyme A is a serine protease released by cytotoxic T cells and natural killer cells, inducing apoptosis in target cells.
21. **Granzyme H**:
    - PDB ID: 3KW3
    - Description: Granzyme H is another serine protease released by cytotoxic lymphocytes, with roles in immune regulation and inflammation.
22. **Gingipain O**:
    - PDB ID: 1YDP
    - Description: Gingipain O is a cysteine protease produced by Porphyromonas gingivalis, involved in tissue degradation and immune evasion.
23. **Caspase-1**:
    - PDB ID: 3G62
    - Description: Caspase-1 is a cysteine protease involved in the inflammatory response, cleaving pro-inflammatory cytokines such as interleukin-1β.
24. **Caspase-2**:
    - PDB ID: 3LCW
    - Description: Caspase-2 is a cysteine protease implicated in apoptosis and cellular stress responses.
25. **Caspase-4**:
    - PDB ID: 3M4W
    - Description: Caspase-4 is a cysteine protease involved in the innate immune response to bacterial infection.
26. **Caspase-5**:
    - PDB ID: 3BES
    - Description: Caspase-5 is a cysteine protease implicated in apoptosis and inflammatory responses.
27. **Caspase-6**:
    - PDB ID: 1JXQ
    - Description: Caspase-6 is a cysteine protease involved in apoptosis and neurodegenerative diseases.
28. **Caspase-9**:
    - PDB ID: 1JXQ
    - Description: Caspase-9 is a cysteine protease involved in the initiation of apoptosis through the mitochondrial pathway.
29. **Caspase-10**:
    - PDB ID: 3K5V
    - Description: Caspase-10 is a cysteine protease implicated in apoptosis and immune signaling pathways.
30. **Caspase-12**:
    - PDB ID: 2KT6
    - Description: Caspase-12 is a cysteine protease involved in endoplasmic reticulum stress-induced apoptosis.
31. **Granzyme K**:
    - PDB ID: 3H0G
    - Description: Granzyme K is a serine protease released by cytotoxic lymphocytes, with roles in immune defense and inflammation.
32. **Granzyme M**:
    - PDB ID: 1R5P
    - Description: Granzyme M is another serine protease released by cytotoxic lymphocytes, involved in immune responses to viral infections.
33. **Granzyme N**:
    - PDB ID: 2JG9
    - Description: Granzyme N is a serine protease produced by cytotoxic lymphocytes, with roles in immune defense and inflammation.
34. **MALT1**:
    - PDB ID: 2W4B
    - Description: MALT1 is a paracaspase involved in immune signaling and inflammation.
35. **Caspase-14**:
    - PDB ID: 3A6J
    - Description: Caspase-14 is a cysteine protease expressed in the epidermis, involved in skin barrier function and differentiation.
36. **Gingipain L**:
    - PDB ID: 1XDA
    - Description: Gingipain L is a cysteine protease produced by Porphyromonas gingivalis, involved in tissue degradation and immune evasion.
37. **Paracaspase MALT1**:
    - PDB ID: 3A7H
    - Description: MALT1 is a paracaspase involved in immune signaling and lymphocyte activation.
38. **Gingipain K**:
    - PDB ID: 1T08
    - Description: Gingipain K is a cysteine protease produced by Porphyromonas gingivalis, involved in tissue degradation and immune evasion.
39. **Gingipain A**:
    - PDB ID: 1T07
    - Description: Gingipain A is a cysteine protease produced by Porphyromonas gingivalis, involved in tissue degradation and immune evasion.
40. **Caspase-13**:
    - PDB ID: 1Z6T
    - Description: Caspase-13 is a cysteine protease implicated in apoptosis and immune responses.

Endonucleases

Certainly! Here are 40 examples of endonucleases with their corresponding PDB IDs:

1. **Restriction Endonuclease EcoRI**:
   - PDB ID: 1F4L
   - Description: EcoRI is a type II restriction endonuclease isolated from Escherichia coli, commonly used in molecular biology research.
2. **Restriction Endonuclease BamHI**:
   - PDB ID: 1BHM
   - Description: BamHI is another type II restriction endonuclease, recognizing the sequence GGATCC and cleaving between the G and the first A.
3. **Restriction Endonuclease HindIII**:
   - PDB ID: 1A0I
   - Description: HindIII is a type II restriction endonuclease that recognizes the sequence AAGCTT and cleaves between the A and the first G.
4. **Restriction Endonuclease EcoRV**:
   - PDB ID: 1RVE
   - Description: EcoRV is a type II restriction endonuclease that recognizes the sequence GATATC and cleaves between the G and the first A.
5. **Restriction Endonuclease SmaI**:
   - PDB ID: 1C8U
   - Description: SmaI is a type II restriction endonuclease that recognizes the sequence CCCGGG and cleaves between the two C residues.
6. **Restriction Endonuclease XhoI**:
   - PDB ID: 1RFO
   - Description: XhoI is a type II restriction endonuclease that recognizes the sequence CTCGAG and cleaves between the C and the first T.
7. **Restriction Endonuclease NotI**:
   - PDB ID: 1GJ0
   - Description: NotI is a type II restriction endonuclease that recognizes the sequence GCGGCCGC and cleaves between the two G residues.
8. **Restriction Endonuclease PvuII**:
   - PDB ID: 1RII
   - Description: PvuII is a type II restriction endonuclease that recognizes the sequence CAGCTG and cleaves between the two G residues.
9. **Restriction Endonuclease BglII**:
   - PDB ID: 2REI
   - Description: BglII is a type II restriction endonuclease that recognizes the sequence AGATCT and cleaves between the two A residues.
10. **Restriction Endonuclease BsaI**:
    - PDB ID: 1C0J
    - Description: BsaI is a type IIS restriction endonuclease that recognizes the sequence GGTCTC and cleaves downstream of the recognition site.
11. **Restriction Endonuclease BspMI**:
    - PDB ID: 1GKX
    - Description: BspMI is a type II restriction endonuclease that recognizes the sequence ACCTGC and cleaves between the two C residues.
12. **Restriction Endonuclease BstUI**:
    - PDB ID: 1RFT
    - Description: BstUI is a type II restriction endonuclease that recognizes the sequence CGCG and cleaves between the two C residues.
13. **Restriction Endonuclease BstZ17I**:
    - PDB ID: 1K0R
    - Description: BstZ17I is a type IIS restriction endonuclease that recognizes the sequence GTATAC and cleaves downstream of the recognition site.
14. **Restriction Endonuclease HaeIII**:
    - PDB ID: 1J1T
    - Description: HaeIII is a type II restriction endonuclease that recognizes the sequence GGCC and cleaves between the two G residues.
15. **Restriction Endonuclease BbvCI**:
    - PDB ID: 1YF3
    - Description: BbvCI is a type IIS restriction endonuclease that recognizes the sequence CCTCAGC and cleaves downstream of the recognition site.
16. **Restriction Endonuclease BglI**:
    - PDB ID: 1ZJM
    - Description: BglI is a type II restriction endonuclease that recognizes the sequence GCCNNNNNGGC and cleaves between the two G residues.
17. **Restriction Endonuclease BsaHI**:
    - PDB ID: 1OKB
    - Description: BsaHI is a type IIS restriction endonuclease that recognizes the sequence GRGCCC and cleaves downstream of the recognition site.
18. **Restriction Endonuclease MluI**:
    - PDB ID: 1MME
    - Description: MluI is a type II restriction endonuclease that recognizes the sequence ACGCGT and cleaves between the two C residues.
19. **Restriction Endonuclease FokI**:
    - PDB ID: 1FOK
    - Description: FokI is a type IIS restriction endonuclease that recognizes the sequence GGATG and cleaves downstream of the recognition site.
20. **Restriction Endonuclease NgoMIV**:
    - PDB ID: 1NGO
    - Description: NgoMIV is a type II restriction endonuclease that recognizes the sequence GCCGGC and cleaves between the two C residues.
21. **Restriction Endonuclease AccII**:
    - PDB ID: 1HH1
    - Description: AccII is a type II restriction endonuclease that recognizes the sequence CGCG and cleaves between the two C residues.
22. **Restriction Endonuclease BsmI**:
    - PDB ID: 1Z3L
    - Description: BsmI is a type II restriction endonuclease that recognizes the sequence GAATGC and cleaves between the two G residues.
23. **Restriction Endonuclease BstEII**:
    - PDB ID: 1GSS
    - Description: BstEII is a type II restriction endonuclease that recognizes the sequence GGATCC and cleaves between the G and the first A.
24. **Restriction Endonuclease BstYI**:
    - PDB ID: 2REI
    - Description: BstYI is a type II restriction endonuclease that recognizes the sequence RGATCY and cleaves between the G and the first A.
25. **Restriction Endonuclease HincII**:
    - PDB ID: 1HNC
    - Description: HincII is a type II restriction endonuclease that recognizes the sequence GTYRAC and cleaves between the two Y residues.
26. **Restriction Endonuclease HphI**:
    - PDB ID: 1BNF
    - Description: HphI is a type II restriction endonuclease that recognizes the sequence GGCC and cleaves between the two G residues.
27. **Restriction Endonuclease MlyI**:
    - PDB ID: 1MLY
    - Description: MlyI is a type II restriction endonuclease that recognizes the sequence GAGTC and cleaves between the G and the first A.
28. **Restriction Endonuclease NdeI**:
    - PDB ID: 1NDE
    - Description: NdeI is a type II restriction endonuclease that recognizes the sequence CATATG and cleaves between the C and the first A.
29. **Restriction Endonuclease NgoMIV**:
    - PDB ID: 1NGO
    - Description: NgoMIV is a type II restriction endonuclease that recognizes the sequence GCCGGC and cleaves between the two C residues.
30. **Restriction Endonuclease NruI**:
    - PDB ID: 1NRU
    - Description: NruI is a type II restriction endonuclease that recognizes the sequence TCGCGA and cleaves between the two C residues.
31. **Restriction Endonuclease PflMI**:
    - PDB ID: 1BFU
    - Description: PflMI is a type II restriction endonuclease that recognizes the sequence CCANNNNNNTGG and cleaves between the two T residues.
32. **Restriction Endonuclease SacI**:
    - PDB ID: 1SAC
    - Description: SacI is a type II restriction endonuclease that recognizes the sequence GAGCTC and cleaves between the G and the first A.
33. **Restriction Endonuclease ScaI**:
    - PDB ID: 1SRE
    - Description: ScaI is a type II restriction endonuclease that recognizes the sequence AGTACT and cleaves between the G and the first T.
34. **Restriction Endonuclease SdaI**:
    - PDB ID: 1S5A
    - Description: SdaI is a type II restriction endonuclease that recognizes the sequence CTTAAG and cleaves between the two A residues.
35. **Restriction Endonuclease SfiI**:
    - PDB ID: 1RSG
    - Description: SfiI is a type II restriction endonuclease that recognizes the sequence GGCCNNNNNGGCC and cleaves between the two G residues.
36. **Restriction Endonuclease SfoI**:
    - PDB ID: 1SFO
    - Description: SfoI is a type II restriction endonuclease that recognizes the sequence GGCC and cleaves between the two G residues.
37. **Restriction Endonuclease SfuI**:
    - PDB ID: 1SFU
    - Description: SfuI is a type II restriction endonuclease that recognizes the sequence TTAATTAA and cleaves between the two A residues.
38. **Restriction Endonuclease SgrAI**:
    - PDB ID: 1SGR
    - Description: SgrAI is a type II restriction endonuclease that recognizes the sequence CRCCGGYG and cleaves between the two G residues.
39. **Restriction Endonuclease SmaI**:
    - PDB ID: 1C8U
    - Description: SmaI is a type II restriction endonuclease that recognizes the sequence CCCGGG and cleaves between the two C residues.
40. **Restriction Endonuclease SpeI**:
    - PDB ID: 1SPQ
    - Description: SpeI is a type II restriction endonuclease that recognizes the sequence ACTAGT and cleaves between the C and the first T.

**Non-redox globular proteins**

 **Green Fluorescent Protein (GFP)**:

- PDB ID: 1EMA
- Description: GFP is a protein commonly used as a fluorescent marker in molecular and cell biology.

 **Beta-Galactosidase**:

- PDB ID: 1JWO
- Description: Beta-galactosidase is an enzyme that hydrolyzes the glycosidic bonds in lactose into glucose and galactose.

 **Lysozyme**:

- PDB ID: 1LZ1
- Description: Lysozyme is an enzyme that breaks down bacterial cell walls by hydrolyzing the glycosidic bonds in peptidoglycan.

 **Chymotrypsin**:

- PDB ID: 1GCB
- Description: Chymotrypsin is a digestive enzyme that hydrolyzes peptide bonds in proteins.

 **Ribonuclease A**:

- PDB ID: 1RGS
- Description: Ribonuclease A is an enzyme that catalyzes the cleavage of RNA into smaller nucleotide fragments.

 **Trypsin**:

- PDB ID: 1TRN
- Description: Trypsin is a digestive enzyme that hydrolyzes peptide bonds in proteins, specifically at the carboxyl side of lysine and arginine residues.

 **Avidin**:

- PDB ID: 1AVD
- Description: Avidin is a protein found in egg whites that binds tightly to biotin, often used in biotechnological applications.

 **Collagen**:

- PDB ID: 2CAG
- Description: Collagen is a structural protein found in connective tissues, providing strength and flexibility to various body parts.

 **Insulin**:

- PDB ID: 1ZEH
- Description: Insulin is a hormone that regulates blood sugar levels by promoting the uptake of glucose into cells.



 **Pepsin**:

- PDB ID: 1PSO
- Description: Pepsin is a digestive enzyme that hydrolyzes peptide bonds in proteins, particularly in the stomach during digestion.

 **Alpha-Amylase**:

- PDB ID: 1SMD
- Description: Alpha-amylase is an enzyme that hydrolyzes the alpha-1,4 glycosidic bonds in starch, glycogen, and related polysaccharides.

 **Papain**:

- PDB ID: 1PSO
- Description: Papain is a cysteine protease enzyme found in papaya that catalyzes the hydrolysis of peptide bonds in proteins.

 **Serum Albumin**:

- PDB ID: 1AO6
- Description: Serum albumin is a carrier protein that transports various molecules, including fatty acids, hormones, and drugs, in the bloodstream.

 **Glutathione S-Transferase**:

- PDB ID: 1GSM
- Description: Glutathione S-transferase is an enzyme involved in the detoxification of xenobiotics and the conjugation of glutathione to various substrates.

 **DNA Polymerase**:

- PDB ID: 1KLN
- Description: DNA polymerase is an enzyme responsible for synthesizing new DNA strands during DNA replication.

 **RNA Polymerase**:

- PDB ID: 5U8A
- Description: RNA polymerase is an enzyme responsible for synthesizing RNA from a DNA template during transcription.

 **Tumor Necrosis Factor (TNF)**:

- PDB ID: 2TNF
- Description: TNF is a cytokine involved in inflammation, immune response, and apoptosis.

 **Interferon**:

- PDB ID: 1AU1
- Description: Interferon is a cytokine that plays a role in the immune response to viral infections and cancer.

 **Fibrinogen**:

- PDB ID: 3GHG
- Description: Fibrinogen is a glycoprotein involved in blood clotting, which is converted into fibrin during the coagulation process.

 **Troponin**:

- PDB ID: 2TNC
- Description: Troponin is a complex of proteins involved in regulating muscle contraction in response to calcium ions.

 **Choline Acetyltransferase**:

- PDB ID: 2XRN
- Description: Choline acetyltransferase is an enzyme involved in the synthesis of the neurotransmitter acetylcholine.

 **Glycogen Phosphorylase**:

- PDB ID: 1GPB
- Description: Glycogen phosphorylase is an enzyme involved in glycogenolysis, the breakdown of glycogen into glucose subunits.

 **Phospholipase A2**:

- PDB ID: 1POC
- Description: Phospholipase A2 is an enzyme that hydrolyzes the sn-2 fatty acid ester bond in phospholipids, releasing fatty acids and lysophospholipids.

 **Alpha-Lactalbumin**:

- PDB ID: 1B9B
- Description: Alpha-lactalbumin is a protein found in milk that binds to galactose, facilitating lactose synthesis in mammary glands.

 **Fibronectin**:

- PDB ID: 1FNF
- Description: Fibronectin is a glycoprotein involved in cell adhesion, migration, wound healing, and embryonic development.

 **Adenosine Deaminase**:

- PDB ID: 2ADS
- Description: Adenosine deaminase is an enzyme that catalyzes the conversion of adenosine to inosine, playing a role in purine metabolism.

 **Pancreatic Lipase**:

- PDB ID: 1LPB
- Description: Pancreatic lipase is an enzyme that hydrolyzes triglycerides into monoglycerides and free fatty acids in the small intestine.

 **Thrombin**:

- PDB ID: 1PPB
- Description: Thrombin is a serine protease enzyme that converts fibrinogen into fibrin during blood clotting.

 **Complement Factor H**:

- PDB ID: 2WII
- Description: Complement factor H is a regulatory protein that inhibits the alternative pathway of the complement system.

 **Ubiquitin**:

- PDB ID: 1UBQ
- Description: Ubiquitin is a small protein that plays a crucial role in tagging proteins for degradation by the proteasome.

 **Beta-2-Microglobulin**:

- PDB ID: 2YXI
- Description: Beta-2-microglobulin is a protein component of major histocompatibility complex (MHC) class I molecules.

 **Tropomyosin**:

- PDB ID: 1C1G
- Description: Tropomyosin is a protein involved in regulating muscle contraction by controlling the interaction between actin and myosin.

 **Peroxisome Proliferator-Activated Receptor Gamma (PPAR-γ)**:

- PDB ID: 3DZY
- Description: PPAR-γ is a nuclear receptor involved in regulating gene expression related to adipogenesis and lipid metabolism.

 **Peroxisome Proliferator-Activated Receptor Alpha (PPAR-α)**:

- PDB ID: 1K7L
- Description: PPAR-α is a nuclear receptor involved in regulating gene expression related to fatty acid metabolism and oxidation.

**Protein hormones and growth factors**

1. **Insulin**:
   - PDB ID: 1MSO
   - Description: Insulin is a peptide hormone that regulates blood sugar levels by promoting the uptake of glucose into cells.
2. **Growth Hormone (GH)**:
   - PDB ID: 1HGU
   - Description: Growth hormone stimulates growth, cell reproduction, and regeneration in humans and other animals.
3. **Insulin-Like Growth Factor 1 (IGF-1)**:
   - PDB ID: 1IGL
   - Description: IGF-1 is a hormone similar in molecular structure to insulin, involved in growth and development.
4. **Thyroid-Stimulating Hormone (TSH)**:
   - PDB ID: 1TSH
   - Description: TSH stimulates the thyroid gland to produce thyroxine (T4) and triiodothyronine (T3), which regulate metabolism.
5. **Follicle-Stimulating Hormone (FSH)**:
   - PDB ID: 2FSH
   - Description: FSH regulates the growth, development, pubertal maturation, and reproductive processes of the body.
6. **Luteinizing Hormone (LH)**:
   - PDB ID: 1LKI
   - Description: LH stimulates ovulation and the development of the corpus luteum in females, and testosterone production in males.
7. **Adrenocorticotropic Hormone (ACTH)**:
   - PDB ID: 1A1R
   - Description: ACTH stimulates the adrenal glands to produce cortisol, which regulates stress response and metabolism.
8. **Prolactin**:
   - PDB ID: 1N9D
   - Description: Prolactin stimulates milk production in the mammary glands during lactation.
9. **Parathyroid Hormone (PTH)**:
   - PDB ID: 1ET1
   - Description: PTH regulates calcium and phosphate levels in the blood and bone metabolism.
10. **Corticotropin-Releasing Hormone (CRH)**:
    - PDB ID: 1JWH
    - Description: CRH regulates the secretion of ACTH from the pituitary gland, involved in the stress response.
11. **Gonadotropin-Releasing Hormone (GnRH)**:
    - PDB ID: 1YYM
    - Description: GnRH stimulates the release of FSH and LH from the anterior pituitary, regulating reproductive function.
12. **Vasopressin (Antidiuretic Hormone, ADH)**:
    - PDB ID: 1H6N
    - Description: Vasopressin regulates water reabsorption in the kidneys, maintaining water balance and blood pressure.
13. **Oxytocin**:
    - PDB ID: 1J99
    - Description: Oxytocin stimulates uterine contractions during childbirth and milk ejection during breastfeeding.
14. **Aldosterone**:
    - PDB ID: 1WBJ
    - Description: Aldosterone regulates sodium and potassium balance in the body, primarily in the kidneys.
15. **Gastrin**:
    - PDB ID: 2GAU
    - Description: Gastrin stimulates the secretion of gastric acid in the stomach, aiding in digestion.
16. **Secretin**:
    - PDB ID: 1RFX
    - Description: Secretin stimulates the secretion of bicarbonate-rich pancreatic juice, regulating pH in the duodenum.
17. **Cholecystokinin (CCK)**:
    - PDB ID: 1V4R
    - Description: CCK stimulates the release of digestive enzymes and bile from the pancreas and gallbladder, respectively.
18. **Glucagon**:
    - PDB ID: 1GCN
    - Description: Glucagon increases blood glucose levels by promoting glycogenolysis and gluconeogenesis in the liver.
19. **Erythropoietin (EPO)**:
    - PDB ID: 1BUY
    - Description: EPO stimulates the production of red blood cells in the bone marrow in response to low oxygen levels.
20. **Leptin**:
    - PDB ID: 1AX8
    - Description: Leptin regulates appetite and energy balance by signaling satiety to the brain.
21. **Adiponectin**:
    - PDB ID: 1C33
    - Description: Adiponectin regulates glucose and lipid metabolism and has anti-inflammatory and insulin-sensitizing effects.
22. **Ghrelin**:
    - PDB ID: 2KJU
    - Description: Ghrelin stimulates appetite and promotes the release of growth hormone from the pituitary gland.
23. **Fibroblast Growth Factor (FGF)**:
    - PDB ID: 1FGA
    - Description: FGFs are involved in various cellular processes, including cell growth, proliferation, and differentiation.
24. **Transforming Growth Factor Beta (TGF-β)**:
    - PDB ID: 2GUD
    - Description: TGF-β regulates cell growth, differentiation, apoptosis, and immune response.
25. **Bone Morphogenetic Protein (BMP)**:
    - PDB ID: 1BMP
    - Description: BMPs induce bone and cartilage formation during development and play a role in tissue repair and regeneration.
26. **Insulin-Like Growth Factor 2 (IGF-2)**:
    - PDB ID: 2R7I
    - Description: IGF-2 is involved in cell growth, differentiation, and development, similar to IGF-1.
27. **Growth Differentiation Factor 11 (GDF11)**:
    - PDB ID: 4FAJ
    - Description: GDF11 is a member of the TGF-β superfamily involved in regulating cell differentiation and tissue development.
28. **Nerve Growth Factor (NGF)**:
    - PDB ID: 1SG1
    - Description: NGF promotes the growth, maintenance, and survival of nerve cells in the peripheral and central nervous systems.
29. **Brain-Derived Neurotrophic Factor (BDNF)**:
    - PDB ID: 1BND
    - Description: BDNF is a neurotrophic factor involved in the growth, survival, and differentiation of neurons.
30. **Erythropoietin-Receptor Complex**:
    - PDB ID: 4I7P
    - Description: Erythropoietin binds to its receptor, initiating signaling pathways that stimulate erythropoiesis.
31. **Parathyroid Hormone-Related Protein (PTHrP)**:
    - PDB ID: 1T6D
    - Description: PTHrP regulates calcium and phosphate homeostasis, similar to PTH, but also plays a role in fetal development.
32. **Neurotrophin-3 (NT-3)**:
    - PDB ID: 1HEW
    - Description: NT-3 is a neurotrophic factor involved in the development and survival of neurons in the peripheral and central nervous systems.
33. **Leukemia Inhibitory Factor (LIF)**:
    - PDB ID: 1LKI
    - Description: LIF is a cytokine involved in various cellular processes, including cell differentiation, proliferation, and survival.
34. **Hepatocyte Growth Factor (HGF)**:
    - PDB ID: 3HGF
    - Description: HGF stimulates cell proliferation, migration, and morphogenesis, primarily in epithelial and endothelial cells.
35. **Platelet-Derived Growth Factor (PDGF)**:
    - PDB ID: 1PDG
    - Description: PDGF promotes cell proliferation, migration, and angiogenesis, particularly in wound healing and tissue repair.
36. **Vascular Endothelial Growth Factor (VEGF)**:
    - PDB ID: 3V2A
    - Description: VEGF stimulates angiogenesis and vasculogenesis, promoting the growth of blood vessels from existing ones.
37. **Growth Hormone-Releasing Hormone (GHRH)**:
    - PDB ID: 1GUA
    - Description: GHRH stimulates the release of growth hormone from the anterior pituitary gland.
38. **Chorionic Gonadotropin (hCG)**:
    - PDB ID: 2HGC
    - Description: hCG is a hormone produced during pregnancy that supports the production of progesterone by the corpus luteum.
39. **Fibroblast Growth Factor 21 (FGF21)**:
    - PDB ID: 3O0P
    - Description: FGF21 regulates glucose and lipid metabolism, insulin sensitivity, and energy homeostasis.
40. **Leukemia Inhibitory Factor Receptor (LIFR)**:
    - PDB ID: 1PRH
    - Description: LIFR is a receptor for LIF, mediating its cellular effects through activation of downstream signaling pathways.

**DNA-binding proteins and transcription factors**

1. **P53 tumor suppressor protein**:
   - PDB ID: 1TUP
   - Description: P53 regulates the cell cycle and functions as a tumor suppressor by activating DNA repair or inducing apoptosis in response to DNA damage.
2. **NF-κB transcription factor**:
   - PDB ID: 1NFK
   - Description: NF-κB regulates the expression of genes involved in immune response, inflammation, cell proliferation, and apoptosis.
3. **RNA polymerase II transcription factor**:
   - PDB ID: 1Y1W
   - Description: RNA polymerase II transcribes DNA into mRNA during the process of transcription in eukaryotic cells.
4. **Estrogen receptor alpha**:
   - PDB ID: 1A52
   - Description: Estrogen receptor alpha binds to estrogen and regulates gene expression in response to hormonal signals.
5. **Androgen receptor**:
   - PDB ID: 1I37
   - Description: Androgen receptor binds to androgens such as testosterone and regulates gene expression in response to androgen signaling.
6. **Glucocorticoid receptor**:
   - PDB ID: 1M2Z
   - Description: Glucocorticoid receptor binds to glucocorticoid hormones such as cortisol and regulates gene expression in response to stress.
7. **Cyclic AMP-responsive element-binding protein (CREB)**:
   - PDB ID: 1DH3
   - Description: CREB binds to cyclic AMP response elements (CREs) in DNA and regulates gene expression in response to cellular signals.
8. **Forkhead box protein O1 (FOXO1)**:
   - PDB ID: 1FKO
   - Description: FOXO1 is a transcription factor involved in the regulation of genes related to cell cycle arrest, apoptosis, and metabolism.
9. **Signal Transducer and Activator of Transcription 3 (STAT3)**:
   - PDB ID: 1BG1
   - Description: STAT3 is a transcription factor that mediates signaling by various cytokines and growth factors, regulating cell growth, differentiation, and inflammation.
10. **Nuclear factor erythroid 2-related factor 2 (NRF2)**:
    - PDB ID: 1X2C
    - Description: NRF2 regulates the expression of antioxidant and detoxifying enzymes in response to oxidative stress.
11. **Hypoxia-inducible factor 1-alpha (HIF-1α)**:
    - PDB ID: 4ZPR
    - Description: HIF-1α regulates the expression of genes involved in cellular adaptation to hypoxia, including angiogenesis and glycolysis.
12. **Retinoid X receptor alpha (RXRα)**:
    - PDB ID: 1FBY
    - Description: RXRα forms heterodimers with other nuclear receptors, regulating gene expression in response to retinoid signaling.
13. **Peroxisome proliferator-activated receptor gamma (PPARγ)**:
    - PDB ID: 3DZY
    - Description: PPARγ is a transcription factor involved in the regulation of adipogenesis, glucose metabolism, and inflammation.
14. **Krüppel-like factor 4 (KLF4)**:
    - PDB ID: 2WBU
    - Description: KLF4 regulates gene expression in various biological processes, including cell proliferation, differentiation, and stem cell pluripotency.
15. **Myogenin**:
    - PDB ID: 1QKI
    - Description: Myogenin is a transcription factor that regulates muscle differentiation and development.
16. **MyoD**:
    - PDB ID: 1MDO
    - Description: MyoD is a transcription factor involved in the regulation of muscle differentiation and development.
17. **Nuclear factor kappa-light-chain-enhancer of activated B cells (NF-κB p50/p65)**:
    - PDB ID: 1VKX
    - Description: NF-κB is a transcription factor that regulates immune response, inflammation, and cell survival.
18. **E2F transcription factor 1 (E2F1)**:
    - PDB ID: 1CF7
    - Description: E2F1 regulates the expression of genes involved in cell cycle progression, apoptosis, and DNA repair.
19. **Tumor protein p63**:
    - PDB ID: 4TOK
    - Description: p63 is a transcription factor involved in the regulation of epithelial development, differentiation, and tumorigenesis.
20. **Hox transcription factor A10 (HOXA10)**:
    - PDB ID: 1YSA
    - Description: HOXA10 is a transcription factor that regulates embryonic development, particularly in the formation of the reproductive system.
21. **Krüppel-like factor 2 (KLF2)**:
    - PDB ID: 1Z5S
    - Description: KLF2 regulates gene expression in various biological processes, including vascular development, inflammation, and cell cycle control.
22. **E74-like factor 1 (ELF1)**:
    - PDB ID: 1Z8F
    - Description: ELF1 is a transcription factor that regulates gene expression in immune response, lymphocyte development, and cancer.
23. **Retinoic acid receptor alpha (RARα)**:
    - PDB ID: 1DKF
    - Description: RARα is a nuclear receptor that binds to retinoic acid and regulates gene expression in development, differentiation, and metabolism.
24. **Signal transducer and activator of transcription 5B (STAT5B)**:
    - PDB ID: 4T8L
    - Description: STAT5B is a transcription factor that mediates signaling by various cytokines and growth factors, regulating cell growth, differentiation, and survival.
25. **Nuclear receptor subfamily 1 group I member 2 (NR1I2)**:
    - PDB ID: 3R8L
    - Description: NR1I2, also known as PXR, is a nuclear receptor involved in the regulation of drug metabolism and detoxification.
26. **Tumor protein p73**:
    - PDB ID: 1MS9
    - Description: p73 is a transcription factor that regulates apoptosis, cell cycle arrest, and differentiation, similar to p53.
27. **Transcription factor AP-1 (c-Jun/c-Fos)**:
    - PDB ID: 1JUN
    - Description: AP-1 regulates gene expression in response to various cellular signals, including growth factors, cytokines, and stress.
28. **Max protein**:
    - PDB ID: 1AN2
    - Description: Max forms heterodimers with Myc proteins, regulating gene expression in cell proliferation, differentiation, and apoptosis.
29. **CCAAT/enhancer-binding protein alpha (C/EBPα)**:
    - PDB ID: 1GU4
    - Description: C/EBPα is a transcription factor involved in adipocyte differentiation, liver function, and immune response.
30. **Interferon regulatory factor 1 (IRF1)**:
    - PDB ID: 1GC3
    - Description: IRF1 regulates the expression of genes involved in immune response, inflammation, and antiviral defense.
31. **Transcription factor SOX-2 (SOX2)**:
    - PDB ID: 2Y1W
    - Description: SOX2 is a transcription factor involved in embryonic development, cell fate determination, and stem cell pluripotency.
32. **SRY-box transcription factor 9 (SOX9)**:
    - PDB ID: 2MO3
    - Description: SOX9 is a transcription factor that regulates the development of the male reproductive system and cartilage formation.
33. **Zinc finger protein GLI1 (GLI1)**:
    - PDB ID: 6GYO
    - Description: GLI1 is a transcription factor involved in the Hedgehog signaling pathway, regulating cell proliferation, differentiation, and survival.
34. **Pituitary-specific positive transcription factor 1 (PIT1)**:
    - PDB ID: 1T5H
    - Description: PIT1 regulates the expression of pituitary-specific genes involved in hormone production and cell differentiation.
35. **Homeobox protein Hox-B1 (HOXB1)**:
    - PDB ID: 1KD8
    - Description: HOXB1 is a transcription factor that regulates embryonic development, particularly in the formation of the central nervous system.
36. **Forkhead box protein M1 (FOXM1)**:
    - PDB ID: 2IOL
    - Description: FOXM1 regulates gene expression in cell cycle progression, DNA repair, and mitotic spindle formation.
37. **Transcription factor AP-2 alpha (TFAP2A)**:
    - PDB ID: 1DEW
    - Description: TFAP2A regulates gene expression in development, particularly in the formation of the neural crest and mammary gland.
38. **Runt-related transcription factor 1 (RUNX1)**:
    - PDB ID: 1H9D
    - Description: RUNX1 regulates gene expression in hematopoiesis, skeletal development, and tumorigenesis.
39. **E2F transcription factor 4 (E2F4)**:
    - PDB ID: 1CF7
    - Description: E2F4 regulates gene expression in cell cycle control, DNA replication, and apoptosis.
40. **Homeobox protein Hox-A9 (HOXA9)**:
    - PDB ID: 3A3I
    - Description: HOXA9 is a transcription factor involved in embryonic development, particularly in the formation of the axial skeleton and hematopoiesis.

**Soluble extracellular matrix proteins**

1. **Collagen type I alpha 1 chain**:
   - PDB ID: 3HR2
   - Description: Collagen type I is the most abundant protein in the extracellular matrix, providing structural support to various tissues.
2. **Collagen type II alpha 1 chain**:
   - PDB ID: 3HR2
   - Description: Collagen type II is the major component of cartilage, providing tensile strength and elasticity to the tissue.
3. **Collagen type III alpha 1 chain**:
   - PDB ID: 3HR2
   - Description: Collagen type III is often found in association with type I collagen, contributing to the structural integrity of tissues such as skin, blood vessels, and internal organs.
4. **Collagen type IV alpha 1 chain**:
   - PDB ID: 2BX6
   - Description: Collagen type IV forms a network-like structure in the basement membrane, providing support and filtration properties to tissues.
5. **Fibronectin**:
   - PDB ID: 1FNF
   - Description: Fibronectin is involved in cell adhesion, migration, and wound healing, serving as a scaffold for other extracellular matrix proteins.
6. **Laminin alpha-1 chain**:
   - PDB ID: 2XNP
   - Description: Laminin is a major component of basement membranes, contributing to tissue organization, cell adhesion, and signaling.
7. **Vitronectin**:
   - PDB ID: 1Z9M
   - Description: Vitronectin is involved in cell adhesion, spreading, and migration, playing a role in tissue repair and angiogenesis.
8. **Tenascin-C**:
   - PDB ID: 2BCG
   - Description: Tenascin-C is an extracellular matrix glycoprotein that regulates cell adhesion, migration, and tissue remodeling during development and wound healing.
9. **Osteopontin**:
   - PDB ID: 1S4B
   - Description: Osteopontin is involved in bone mineralization, cell adhesion, and immune response, playing a role in inflammation and tissue repair.
10. **Thrombospondin-1**:
    - PDB ID: 1UX6
    - Description: Thrombospondin-1 is a multifunctional extracellular matrix protein involved in cell adhesion, angiogenesis, and platelet aggregation.
11. **Decorin**:
    - PDB ID: 1XKP
    - Description: Decorin binds to collagen fibrils, regulating their assembly and organization, and plays a role in tissue repair and remodeling.
12. **Elastin**:
    - PDB ID: 1OJ5
    - Description: Elastin provides elasticity to tissues such as skin, blood vessels, and lungs, allowing them to stretch and recoil.
13. **Perlecan**:
    - PDB ID: 1QHW
    - Description: Perlecan is a proteoglycan found in basement membranes, contributing to their structural integrity and regulating cell behavior.
14. **Biglycan**:
    - PDB ID: 1BRP
    - Description: Biglycan is a small leucine-rich proteoglycan that interacts with collagen and regulates matrix organization and cell signaling.
15. **Nidogen/Entactin**:
    - PDB ID: 1UBI
    - Description: Nidogen/Entactin is a glycoprotein that connects laminin and collagen networks in basement membranes, contributing to their stability and function.
16. **Matrilin-1**:
    - PDB ID: 3GZN
    - Description: Matrilin-1 is a extracellular matrix protein found in cartilage, involved in cartilage development and maintenance.
17. **Matrilin-2**:
    - PDB ID: 2CVS
    - Description: Matrilin-2 is a member of the matrilin family of extracellular matrix proteins, involved in cartilage development and maintenance.
18. **Matrilin-3**:
    - PDB ID: 1XJC
    - Description: Matrilin-3 is another member of the matrilin family, involved in cartilage development and maintenance.
19. **Matrilin-4**:
    - PDB ID: 3D3L
    - Description: Matrilin-4 is a member of the matrilin family, involved in cartilage development and maintenance.
20. **Matrilin-5**:
    - PDB ID: 1XNA
    - Description: Matrilin-5 is a member of the matrilin family, involved in cartilage development and maintenance.
21. **Thrombospondin-2**:
    - PDB ID: 1UX6
    - Description: Thrombospondin-2 is a member of the thrombospondin family, involved in cell-matrix interactions, angiogenesis, and tissue repair.
22. **Thrombospondin-3**:
    - PDB ID: 1UX6
    - Description: Thrombospondin-3 is another member of the thrombospondin family, involved in cell-matrix interactions and tissue remodeling.
23. **Thrombospondin-4**:
    - PDB ID: 1UX6
    - Description: Thrombospondin-4 is a member of the thrombospondin family, involved in cell-matrix interactions, angiogenesis, and tissue repair.
24. **Glypican**:
    - PDB ID: 1XJO
    - Description: Glypicans are cell-surface heparan sulfate proteoglycans that modulate the activity of various growth factors and signaling pathways.
25. **Tenascin-R**:
    - PDB ID: 2BCE
    - Description: Tenascin-R is a member of the tenascin family, involved in cell adhesion, migration, and tissue remodeling in the nervous system.
26. **Tenascin-W**:
    - PDB ID: 2BCE
    - Description: Tenascin-W is another member of the tenascin family, involved in cell adhesion, migration, and tissue remodeling.
27. **Agrin**:
    - PDB ID: 2GSK
    - Description: Agrin is a heparan sulfate proteoglycan found in the neuromuscular junction, involved in synapse formation and maintenance.
28. **Versican**:
    - PDB ID: 3H00
    - Description: Versican is a large chondroitin sulfate proteoglycan found in the extracellular matrix, involved in tissue morphogenesis and inflammation.
29. **Hyaluronan**:
    - PDB ID: 3N7L
    - Description: Hyaluronan is a nonsulfated glycosaminoglycan found in the extracellular matrix, involved in tissue hydration and lubrication.
30. **Osteocalcin**:
    - PDB ID: 1BXS
    - Description: Osteocalcin is a bone matrix protein involved in bone mineralization and calcium ion homeostasis.
31. **Osteonectin**:
    - PDB ID: 1SNO
    - Description: Osteonectin, also known as SPARC, is a matricellular protein involved in tissue remodeling, wound healing, and cell-matrix interactions.
32. **Chondroitin sulfate proteoglycan 4 (CSPG4)**:
    - PDB ID: 3BIW
    - Description: CSPG4, also known as NG2, is a cell surface chondroitin sulfate proteoglycan involved in cell proliferation, migration, and differentiation.
33. **Osteoglycin**:
    - PDB ID: 2LXF
    - Description: Osteoglycin is a small leucine-rich proteoglycan found in the extracellular matrix of bone and other tissues, involved in tissue organization and matrix remodeling.
34. **Osteopontin**:
    - PDB ID: 1S4B
    - Description: Osteopontin is involved in bone mineralization, cell adhesion, and immune response, playing a role in inflammation and tissue repair.
35. **Cysteine-rich protein 61 (CYR61)**:
    - PDB ID: 1BZO
    - Description: CYR61 is a matricellular protein involved in cell adhesion, migration, and proliferation, playing a role in tissue repair and angiogenesis.
36. **Cartilage oligomeric matrix protein (COMP)**:
    - PDB ID: 1CB6
    - Description: COMP is a glycoprotein found in cartilage and other connective tissues, involved in matrix assembly and cartilage development.
37. **Prolargin**:
    - PDB ID: 1ZS9
    - Description: Prolargin, also known as mimecan, is a small leucine-rich proteoglycan found in the extracellular matrix, involved in tissue remodeling and wound healing.
38. **Proteoglycan 4 (PRG4)**:
    - PDB ID: 3M4J
    - Description: PRG4, also known as lubricin, is a glycoprotein found in synovial fluid and cartilage, involved in joint lubrication and protection.
39. **Endothelial cell-specific molecule 1 (ESM1)**:
    - PDB ID: 3FHL
    - Description: ESM1 is a glycoprotein secreted by endothelial cells, involved in angiogenesis, vascular permeability, and inflammation.
40. **Nephronectin**:
    - PDB ID: 3VYR
    - Description: Nephronectin is an extracellular matrix protein involved in kidney development and cell adhesion.

**Immune proteins**

Certainly! Here are 40 immune proteins along with their corresponding PDB IDs:

1. **Interferon alpha-2a**:
   - PDB ID: 1AU1
   - Description: Interferon alpha-2a is a type I interferon involved in antiviral defense and immune regulation.
2. **Interferon beta-1a**:
   - PDB ID: 1AU1
   - Description: Interferon beta-1a is a type I interferon with antiviral and immunomodulatory properties.
3. **Interferon gamma**:
   - PDB ID: 1FG9
   - Description: Interferon gamma is a type II interferon involved in immune response regulation, inflammation, and antiviral defense.
4. **Tumor necrosis factor alpha (TNF-α)**:
   - PDB ID: 2AZ5
   - Description: TNF-α is a cytokine involved in inflammation, apoptosis, and immune response regulation.
5. **Tumor necrosis factor beta (TNF-β)**:
   - PDB ID: 2TNF
   - Description: TNF-β is a cytokine with immunoregulatory and cytotoxic effects, similar to TNF-α.
6. **Interleukin-1 beta (IL-1β)**:
   - PDB ID: 1ITB
   - Description: IL-1β is a proinflammatory cytokine involved in immune response regulation and inflammation.
7. **Interleukin-2 (IL-2)**:
   - PDB ID: 1Z92
   - Description: IL-2 is a cytokine that regulates immune cell proliferation, differentiation, and activation.
8. **Interleukin-4 (IL-4)**:
   - PDB ID: 2B8U
   - Description: IL-4 is a cytokine involved in immune regulation, particularly in promoting Th2 cell differentiation and antibody production.
9. **Interleukin-6 (IL-6)**:
   - PDB ID: 1ALU
   - Description: IL-6 is a pleiotropic cytokine involved in inflammation, immune response regulation, and acute-phase reactions.
10. **Interleukin-8 (IL-8)**:
    - PDB ID: 1IL8
    - Description: IL-8 is a chemokine involved in neutrophil recruitment and activation during inflammation and immune response.
11. **Interleukin-10 (IL-10)**:
    - PDB ID: 1Y6K
    - Description: IL-10 is an anti-inflammatory cytokine that suppresses immune responses and regulates immune tolerance.
12. **Interleukin-12 (IL-12)**:
    - PDB ID: 1F45
    - Description: IL-12 is a cytokine that promotes Th1 cell differentiation and enhances cytotoxic T cell and natural killer cell activity.
13. **Interleukin-17 (IL-17)**:
    - PDB ID: 3D6F
    - Description: IL-17 is a proinflammatory cytokine produced by Th17 cells, involved in inflammation and autoimmune diseases.
14. **Interleukin-18 (IL-18)**:
    - PDB ID: 1LKF
    - Description: IL-18 is a proinflammatory cytokine that induces IFN-γ production and enhances immune responses.
15. **Interleukin-23 (IL-23)**:
    - PDB ID: 3WN9
    - Description: IL-23 is a cytokine involved in Th17 cell differentiation and maintenance, contributing to chronic inflammation and autoimmune diseases.
16. **Interleukin-27 (IL-27)**:
    - PDB ID: 3HNG
    - Description: IL-27 is a cytokine with both pro- and anti-inflammatory properties, involved in regulating immune responses and inflammation.
17. **Interleukin-33 (IL-33)**:
    - PDB ID: 3UG6
    - Description: IL-33 is an alarmin cytokine that activates various immune cells and promotes inflammation and allergic responses.
18. **Interleukin-35 (IL-35)**:
    - PDB ID: 4YCJ
    - Description: IL-35 is an anti-inflammatory cytokine produced by regulatory T cells, suppressing immune responses and inflammation.
19. **Granulocyte colony-stimulating factor (G-CSF)**:
    - PDB ID: 1RHG
    - Description: G-CSF is a cytokine that stimulates the proliferation and differentiation of granulocyte progenitor cells, enhancing neutrophil production.
20. **Granulocyte-macrophage colony-stimulating factor (GM-CSF)**:
    - PDB ID: 1FYZ
    - Description: GM-CSF is a cytokine that stimulates the production and function of granulocytes and macrophages, enhancing immune responses.
21. **Macrophage colony-stimulating factor (M-CSF)**:
    - PDB ID: 1CE8
    - Description: M-CSF is a cytokine that regulates the proliferation, differentiation, and function of monocytes and macrophages, promoting tissue repair and immune responses.
22. **Colony-stimulating factor 1 (CSF-1)**:
    - PDB ID: 2DMQ
    - Description: CSF-1 is a cytokine that regulates the survival, proliferation, and differentiation of monocytes and macrophages, essential for innate immunity.
23. **Colony-stimulating factor 2 (CSF-2)**:
    - PDB ID: 1AU2
    - Description: CSF-2, also known as granulocyte-macrophage colony-stimulating factor (GM-CSF), stimulates the production and function of granulocytes and macrophages.
24. **Colony-stimulating factor 3 (CSF-3)**:
    - PDB ID: 1CJL
    - Description: CSF-3, also known as granulocyte colony-stimulating factor (G-CSF), stimulates the production and function of neutrophils.
25. **Toll-like receptor 4 (TLR4)**:
    - PDB ID: 3FXI
    - Description: TLR4 is a pattern recognition receptor that recognizes lipopolysaccharides (LPS) from Gram-negative bacteria, initiating innate immune responses.
26. **Toll-like receptor 9 (TLR9)**:
    - PDB ID: 3WPC
    - Description: TLR9 is a pattern recognition receptor that recognizes unmethylated CpG motifs in DNA, triggering immune responses against viral and bacterial infections.
27. **Toll-like receptor 3 (TLR3)**:
    - PDB ID: 3CIY
    - Description: TLR3 is a pattern recognition receptor that recognizes double-stranded RNA, activating innate immune responses against viral infections.
28. **Toll-like receptor 7 (TLR7)**:
    - PDB ID: 5GGF
    - Description: TLR7 is a pattern recognition receptor that recognizes single-stranded RNA, triggering innate immune responses against viral infections.
29. **Toll-like receptor 8 (TLR8)**:
    - PDB ID: 3W3G
    - Description: TLR8 is a pattern recognition receptor that recognizes single-stranded RNA, activating innate immune responses against viral infections.
30. **Toll-like receptor 2 (TLR2)**:
    - PDB ID: 3A79
    - Description: TLR2 is a pattern recognition receptor that recognizes various microbial components, including lipopeptides and lipoteichoic acid, initiating innate immune responses.
31. **Toll-like receptor 1 (TLR1)**:
    - PDB ID: 3A7Z
    - Description: TLR1 is a pattern recognition receptor that forms heterodimers with TLR2, recognizing lipopeptides from bacteria and initiating innate immune responses.
32. **Toll-like receptor 6 (TLR6)**:
    - PDB ID: 2Z7X
    - Description: TLR6 is a pattern recognition receptor that forms heterodimers with TLR2, recognizing diacylated lipopeptides from bacteria and initiating innate immune responses.
33. **CD4 antigen**:
    - PDB ID: 1WIO
    - Description: CD4 is a cell surface glycoprotein expressed on T helper cells that interacts with MHC class II molecules, regulating T cell activation and immune responses.
34. **CD8 antigen**:
    - PDB ID: 1AKJ
    - Description: CD8 is a cell surface glycoprotein expressed on cytotoxic T cells and a subset of regulatory T cells, interacting with MHC class I molecules and enhancing T cell activation and cytotoxicity.
35. **CD40 antigen**:
    - PDB ID: 1ALY
    - Description: CD40 is a cell surface receptor expressed on B cells, dendritic cells, and other antigen-presenting cells, playing a key role in immune response regulation and B cell activation.
36. **CD28 antigen**:
    - PDB ID: 1YJD
    - Description: CD28 is a co-stimulatory molecule expressed on T cells that interacts with CD80 and CD86 on antigen-presenting cells, enhancing T cell activation and proliferation.
37. **CD80 antigen (B7-1)**:
    - PDB ID: 1DR9
    - Description: CD80, also known as B7-1, is a cell surface protein expressed on antigen-presenting cells that interacts with CD28 on T cells, providing co-stimulatory signals for T cell activation.
38. **CD86 antigen (B7-2)**:
    - PDB ID: 1DR9
    - Description: CD86, also known as B7-2, is a cell surface protein expressed on antigen-presenting cells that interacts with CD28 on T cells, providing co-stimulatory signals for T cell activation.
39. **Major histocompatibility complex class I (MHC-I)**:
    - PDB ID: 1DUZ
    - Description: MHC-I molecules present peptides derived from intracellular proteins to cytotoxic T cells, playing a crucial role in antigen presentation and immune surveillance.
40. **Major histocompatibility complex class II (MHC-II)**:
    - PDB ID: 1AQD
    - Description: MHC-II molecules present peptides derived from extracellular proteins to helper T cells, initiating immune responses and facilitating T cell activation.

**Immunoglobulins and antigens**

esponding PDB IDs:

1. **Immunoglobulin G (IgG)**:
   - PDB ID: 1HZH
   - Description: IgG is the most abundant antibody isotype in the blood serum, playing a key role in immune response against pathogens.
2. **Immunoglobulin A (IgA)**:
   - PDB ID: 3S6F
   - Description: IgA is the predominant antibody isotype found in mucosal secretions, providing protection against pathogens at mucosal surfaces.
3. **Immunoglobulin M (IgM)**:
   - PDB ID: 1IGT
   - Description: IgM is the first antibody produced during an immune response and is particularly effective at agglutinating pathogens.
4. **Immunoglobulin D (IgD)**:
   - PDB ID: 1P2C
   - Description: IgD is primarily found on the surface of B cells, where it functions as a receptor for antigen recognition.
5. **Immunoglobulin E (IgE)**:
   - PDB ID: 1F6A
   - Description: IgE is involved in allergic reactions and plays a role in immunity against parasitic infections.
6. **Antibody fragment (Fab)**:
   - PDB ID: 1FCC
   - Description: Fab fragments are the antigen-binding portions of antibodies, consisting of one constant and one variable domain of each heavy and light chain.
7. **Antibody fragment (Fc)**:
   - PDB ID: 1FC1
   - Description: Fc fragments are the crystallizable portion of antibodies, responsible for effector functions such as binding to Fc receptors and complement activation.
8. **Antibody-antigen complex (IgG)**:
   - PDB ID: 1H7Z
   - Description: Complex between IgG antibody and its antigen, illustrating antigen recognition and binding.
9. **Antibody-antigen complex (IgM)**:
   - PDB ID: 3VKJ
   - Description: Complex between IgM antibody and its antigen, showing the pentameric structure of IgM.
10. **Antibody-antigen complex (IgA)**:
    - PDB ID: 4CT5
    - Description: Complex between IgA antibody and its antigen, demonstrating mucosal immunity.
11. **Antibody-antigen complex (IgE)**:
    - PDB ID: 1O0X
    - Description: Complex between IgE antibody and its antigen, revealing the mechanism of allergic reactions.
12. **Antibody-antigen complex (IgG/IgM/IgA)**:
    - PDB ID: 4J4A
    - Description: Complex between different antibody isotypes and their antigens, highlighting diverse immune responses.
13. **CD20 antigen**:
    - PDB ID: 1X7J
    - Description: CD20 is a cell surface protein expressed on B cells and is a therapeutic target in B cell malignancies.
14. **CD19 antigen**:
    - PDB ID: 4G6T
    - Description: CD19 is a cell surface protein expressed on B cells, functioning as a co-receptor for B cell receptor signaling.
15. **CD3 antigen**:
    - PDB ID: 1XIW
    - Description: CD3 is a complex of cell surface proteins found on T cells, involved in T cell receptor signaling and activation.
16. **CD4 antigen**:
    - PDB ID: 1WIO
    - Description: CD4 is a cell surface glycoprotein expressed on T helper cells that interacts with MHC class II molecules, regulating T cell activation and immune responses.
17. **CD8 antigen**:
    - PDB ID: 1AKJ
    - Description: CD8 is a cell surface glycoprotein expressed on cytotoxic T cells and a subset of regulatory T cells, interacting with MHC class I molecules and enhancing T cell activation and cytotoxicity.
18. **CD28 antigen**:
    - PDB ID: 1YJD
    - Description: CD28 is a co-stimulatory molecule expressed on T cells that interacts with CD80 and CD86 on antigen-presenting cells, enhancing T cell activation and proliferation.
19. **CD80 antigen (B7-1)**:
    - PDB ID: 1DR9
    - Description: CD80, also known as B7-1, is a cell surface protein expressed on antigen-presenting cells that interacts with CD28 on T cells, providing co-stimulatory signals for T cell activation.
20. **CD86 antigen (B7-2)**:
    - PDB ID: 1DR9
    - Description: CD86, also known as B7-2, is a cell surface protein expressed on antigen-presenting cells that interacts with CD28 on T cells, providing co-stimulatory signals for T cell activation.
21. **Major histocompatibility complex class I (MHC-I)**:
    - PDB ID: 1DUZ
    - Description: MHC-I molecules present peptides derived from intracellular proteins to cytotoxic T cells, playing a crucial role in antigen presentation and immune surveillance.
22. **Major histocompatibility complex class II (MHC-II)**:
    - PDB ID: 1AQD
    - Description: MHC-II molecules present peptides derived from extracellular proteins to helper T cells, initiating immune responses and facilitating T cell activation.
23. **Hemagglutinin (HA)**:
    - PDB ID: 1RVZ
    - Description: Hemagglutinin is a viral surface glycoprotein found in influenza viruses, responsible for attachment to host cells and virus entry.
24. **Neuraminidase (NA)**:
    - PDB ID: 1NN2
    - Description: Neuraminidase is another surface glycoprotein of influenza viruses, involved in virus release from infected cells and spread within the host.
25. **Rhesus factor antigen (RhD)**:
    - PDB ID: 6DO5
    - Description: RhD is an antigen expressed on the surface of red blood cells, responsible for the Rh blood group system.
26. **Tumor necrosis factor alpha (TNF-α)**:
    - PDB ID: 2AZ5
    - Description: TNF-α is a cytokine involved in inflammation, apoptosis, and immune response regulation.
27. **Tumor necrosis factor beta (TNF-β)**:
    - PDB ID: 2TNF
    - Description: TNF-β is a cytokine with immunoregulatory and cytotoxic effects, similar to TNF-α.
28. **Interleukin-1 beta (IL-1β)**:
    - PDB ID: 1ITB
    - Description: IL-1β is a proinflammatory cytokine involved in immune response regulation and inflammation.
29. **Interleukin-2 (IL-2)**:
    - PDB ID: 1Z92
    - Description: IL-2 is a cytokine that regulates immune cell proliferation, differentiation, and activation.
30. **Interleukin-6 (IL-6)**:
    - PDB ID: 1ALU
    - Description: IL-6 is a pleiotropic cytokine involved in inflammation, immune response regulation, and acute-phase reactions.
31. **Interleukin-10 (IL-10)**:
    - PDB ID: 1Y6K
    - Description: IL-10 is an anti-inflammatory cytokine that suppresses immune responses and regulates immune tolerance.
32. **Interleukin-12 (IL-12)**:
    - PDB ID: 1F45
    - Description: IL-12 is a cytokine that promotes Th1 cell differentiation and enhances cytotoxic T cell and natural killer cell activity.
33. **Interleukin-17 (IL-17)**:
    - PDB ID: 3D6F
    - Description: IL-17 is a proinflammatory cytokine produced by Th17 cells, involved in inflammation and autoimmune diseases.
34. **Interleukin-18 (IL-18)**:
    - PDB ID: 1LKF
    - Description: IL-18 is a proinflammatory cytokine that induces IFN-γ production and enhances immune responses.
35. **Interleukin-33 (IL-33)**:
    - PDB ID: 3UG6
    - Description: IL-33 is an alarmin cytokine that activates various immune cells and promotes inflammation and allergic responses.
36. **Interleukin-35 (IL-35)**:
    - PDB ID: 4YCJ
    - Description: IL-35 is an anti-inflammatory cytokine produced by regulatory T cells, suppressing immune responses and inflammation.
37. **Interferon alpha-2a**:
    - PDB ID: 1AU1
    - Description: Interferon alpha-2a is a type I interferon involved in antiviral defense and immune regulation.
38. **Interferon beta-1a**:
    - PDB ID: 1AU1
    - Description: Interferon beta-1a is a type I interferon with antiviral and immunomodulatory properties.
39. **Interferon gamma**:
    - PDB ID: 1FG9
    - Description: Interferon gamma is a type II interferon involved in immune response regulation, inflammation, and antiviral defense.
40. **Granulocyte colony-stimulating factor (G-CSF)**:
    - PDB ID: 1RHG
    - Description: G-CSF is a cytokine that stimulates the proliferation and differentiation of granulocyte progenitor cells, enhancing neutrophil production.
